# Controlled Polymer Synthesis Toward Green Chemistry: Deep Insights into Atom Transfer Radical Polymerization in Biobased Substitutes for Polar Aprotic Solvents

**DOI:** 10.1021/acssuschemeng.3c07993

**Published:** 2024-02-21

**Authors:** Izabela Zaborniak, Małgorzata Klamut, Cicely M. Warne, Katarzyna Kisiel, Martyna Niemiec, Paweł Błoniarz, Alessandro Pellis, Krzysztof Matyjaszewski, Paweł Chmielarz

**Affiliations:** † Department of Physical Chemistry, Faculty of Chemistry, Rzeszow University of Technology, al. Powstańców Warszawy 6, Rzeszów 35-959, Poland; ‡ Department of Chemistry, Carnegie Mellon University, 4400 Fifth Ave., Pittsburgh, Pennsylvania 15213, United States; § Doctoral School of the Rzeszów University of Technology, al. Powstańców Warszawy 8, Rzeszów 35-959, Poland; ± Institute for Environmental Biotechnology, Department for Agrobiotechnology, University of Natural Resources and Life Sciences, Konrad Lorenz Strasse 20, Tulln an der Donau A-3430, Austria; ∥ ACIB GmbH, Konrad-Lorenz-Strasse 20, Tulln an der Donau 3430, Austria; ⊥ Department of Chemistry and Industrial Chemistry, University of Genova, Via Dodecaneso 31, Genova 16146, Italy

**Keywords:** Cyrene, Cygnet 0.0, low ppm atom transfer radical polymerization, well-defined polymer, branched polymers

## Abstract

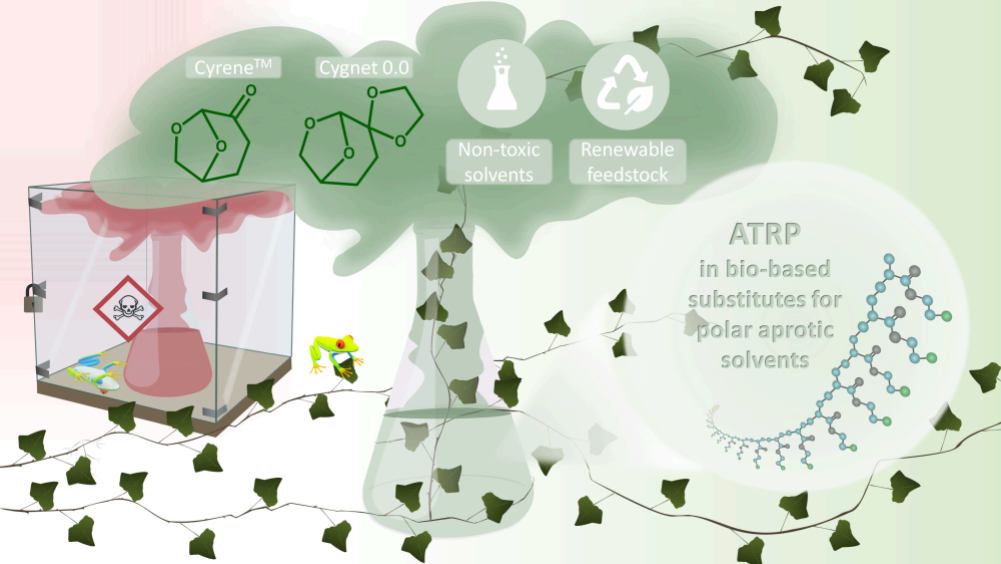

Cyrene (dihydrolevoglucosenone) and its derivative Cygnet 0.0, recognized as eco-friendly alternatives to polar aprotic solvents, were utilized in atom transfer radical polymerization (ATRP) of a wide range of both hydrophobic and hydrophilic (meth)acrylates. The detailed kinetics study and electrochemical experiments of the catalytic complex in these solvents reveal the opportunities and limitations of their use in controlled radical polymerization. Both solvents produce precisely controlled polymers using supplemental activator and reducing agent (SARA) ATRP. They offer an efficient reaction medium for crafting well-defined branched architectures from naturally derived cores such as riboflavin, β-cyclodextrin, and troxerutin, thereby significantly expanding the application scope of these solvents. Notably, Cygnet 0.0 significantly reduces side reactions between the solvent and the catalyst compared to Cyrene, allowing the catalyst complex to be used at a reduced concentration down to 75 ppm. The effective mass yield values achieved in Cyrene and Cygnet 0.0 underscore a substantial advantage of these solvents over DMF in generating processes that adhere to the principles of green chemistry. Furthermore, the copper residue in the final polymers was several hundred times lower than the permissible daily exposure to orally administered copper in pharmaceuticals. As a result, the resulting polymeric materials hold immense potential for various applications, including the pharmaceutical industry.

## Abstract 2

The use of biobased substitutes for harmful polar aprotic solvents as a polymerization environment represents a significant advance toward sustainable and green polymer chemistry.

## Introduction

The polymer industry significantly impacts our society as polymers have become pervasive, forming an inseparable part of our daily lives. Synthetic polymer and functional material applications include, e.g., food and medical packaging materials, medical devices that improve quality of life, new materials for biomedical applications, microelectronics, sustainable power generation and energy storage, functional coatings, and materials in the automotive and aerospace industries.^1−3^ Due to their versatile applications and large-scale production, there is growing attention not only on the final product quality but also on sustainable development and environmental considerations,^4,5^ within the framework of the principles of green chemistry^6^ and green engineering^7^ in chemical processes.

Numerous synthetic methods and processing techniques for polymers involve the use of organic solvents. These solvents represent a significant category within the chemical industry, with an annual market that reaches millions of tons, and most often, they show the characteristics of toxic substances.^8,9^ The utilization of harmful organic compounds in the synthesis of desired products stands as a significant barrier to the swift advancement of modern polymer chemistry, for example, based on fully controlled polymer materials. The existing techniques that have been developed and have the potential for scaling up often involve hazardous wastes, which might inherently lead to environmental and health concerns. Therefore, in accordance with green chemistry aspects, it is crucial to avoid the use of toxic solvents, known as volatile organic compounds (VOCs), as they contribute to waste generation during synthesis.^10^ Their extensive use in reaction mixtures and the separation of polymer products result in this issue.

Moving toward the elimination of toxic solvents in syntheses carried out using controlled polymerization techniques, including the atom transfer radical polymerization (ATRP) methods, the directions is the development of environmentally friendly replacement solvents that can eliminate toxicity from the reaction mixture without affecting other reaction parameters.^11−13^ Nevertheless, this task can be intricate, as solvents serve diverse roles in polymerization. They function to solubilize reagents such as monomers and polymers, maintain a homogeneous mixture, facilitate heat transfer, and influence activation rate constants, which is critical in controlling polymer chain growth.^14−17^ In certain instances, substituting a solvent may necessitate procedure adjustments to support the reaction’s advancement and guarantee optimal outcomes. This change might significantly alter certain process characteristics, leading to reactions that are notably faster.

Water stands as the most benign solvent in the ATRP of hydrophilic monomers.^18−23^ Although polymerization in an aqueous environment is environmentally friendly, it has limitations due to certain side reactions. These include disproportionation of Cu^I^ activator complexes, the dissociation of the halide from higher oxidation state complexes leading to the formation of inactive species that cannot undergo deactivation, and the hydrolysis of the initiator, causing the creation of dead chains.^18−20^ These occurrences suggest a loss of control over the molecular weight of the final polymers. An alternative method involves using alcohols, alcoholic beverages, or alcohol/water mixtures.^24−26^ However, while these solvents are less toxic, their presence in the reaction mixture results in a faster progression of the reaction, leading to various side reactions associated with increased activity of the catalyst complex. The high concentration of radicals prompts an uncontrolled rapid termination of polymer chains, resulting in final polymers characterized by a relatively broad molecular weight distribution (MWD, *M*_w_/*M*_n_). The polymerization of hydrophobic monomers also can be conducted in dispersed in aqueous environments like suspension,^27^ emulsion,^28^ miniemulsion,^29,30^ or microemulsion,^31,32^ instead of harmful organic solvents. However, the usability of the concept is limited to a narrow range of monomer types, and the syntheses are carried out in a high dilution of the organic monomer phase. Thus, it generates a large amount of waste.

These approaches cannot replace the commonly used solvents in ATRP like dimethylformamide (DMF),^33−36^ dimethyl sulfoxide (DMSO),^36−39^ anisole,^40^ tetrahydrofuran (THF),^36,41^*N*-methyl-2-pyrrolidone (NMP),^36,38^ toluene,^42^ or dimethyl acetate (DMAc),^41^ which are the most frequently used reaction environments for fully controlled polymerizations of both hydrophilic and hydrophobic monomers. Nevertheless, these solvents are also restricted by Registration, Evaluation, Authorization, and Restriction of Chemicals (REACH) regulation, and therefore, their application in industrial processes is significantly limited. The promising types of benign solvents for polymerization are ionic liquids,^43−45^ supercritical carbon dioxide,^46^ deep eutectic solvents,^44,47−49^ gas expanded solvents,^50^ and switchable solvents.^42^

Recently, there has been a growing interest in exploring nontoxic, bioderived alternatives that possess similar properties to the abovementioned chemicals reflected by the Hansen solubility parameters, aiming to achieve equally effective processes and controlled polymer structures. One such alternative proposed is 2-methyltetrahydrofuran (2-MeTHF), serving as a green substitute for solvents akin to THF.^51^ Additionally, a new type of aprotic dipolar solvent, dihydrolevoglucosenone (Cyrene), has garnered increasing attention. This solvent, derived from cellulose decomposition,^52^ has gained prominence due to its fully degradable and nonmutagenic nature. Circa Group has recently started small-scale production of Cyrene as a replacement for solvents like DCM, NMP, DMF, or DMAc.^53,54^ It is important to note that Cyrene demonstrates dipolarity akin to highly dipolar aprotic solvents.^55^ Previous studies have shown its utility in ATRP for Cu(0) wire-mediated RDRP, displaying an efficient ability to facilitate monomer polymerization while maintaining control over the polymer chain growth.^56^ Moreover, Cyrene is a versatile compound, capable of forming derivatives like cygnets, obtained through a reaction between Cyrene and ethylene glycol. Such derivatives hold promise as potential replacement solvents for dichloromethane (DCM),^57−59^ but to the best of our knowledge, Cyrene derivatives, i.e., cygnets, have not yet been used in polymerization using RDRP techniques.

**Scheme 1:**
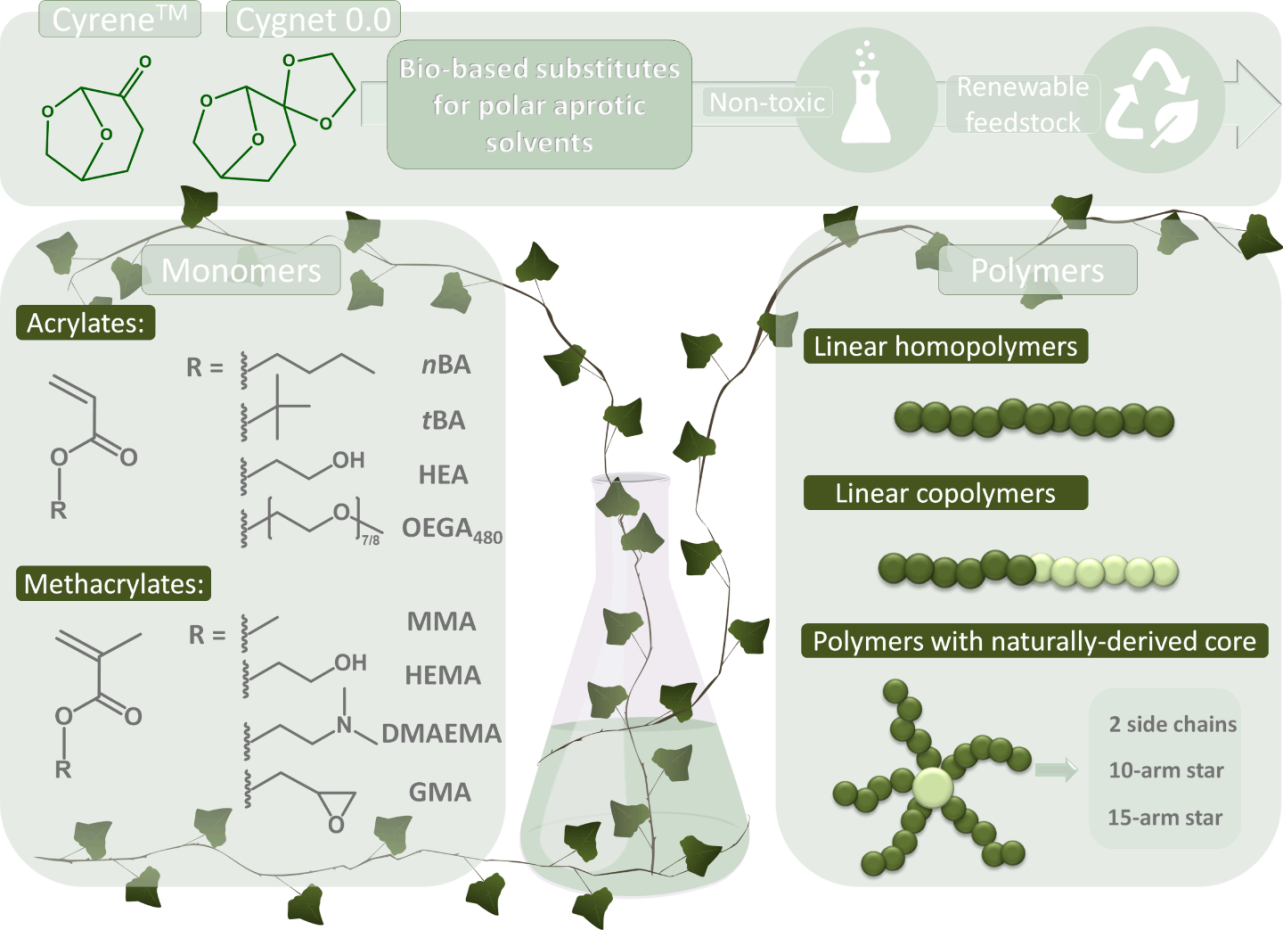
Scope of Applicability of Green Solvents Presented in the Work

This paper primarily focuses on further exploring the applicability of Cyrene as an environmentally friendly solvent for polymerizing various methacrylates and acrylates. This study aims to extend the utility of Cyrene in synthesizing polymers with diverse architectures using naturally derived cores. Additionally, the paper aims to present the opportunities and limitations of this solvent across various ATRP techniques that are controlled by both external stimuli and chemical compounds. Notably, we are excited to report the successful implementation of Cygnet 0.0 in ATRP for different monomers, leading to the preparation of macromolecules with a branched architecture (Scheme 1).

All of these reactions were conducted under different conditions to assess the versatility and efficacy of the two solvents. Furthermore, in quantifying the superiority of the utilized solvents over their toxic counterparts, green chemistry metrics were applied, primarily the effective mass yield (EMY). This metric measures the mass of the desired product concerning all environmentally unfriendly materials used. Another critical aspect for the practical application of the obtained polymers, especially in fields such as medicine, pharmacy, or diverse industrial uses, involves contamination with catalyst residues. Hence, the copper residue present in the final polymers was assessed by using atomic absorption spectroscopy (AAS). The utilization of nontoxic biobased substitutes for hazardous polar aprotic solvents as opposed to conventional hazardous organic solvents marks a stride forward in advancing the production scalability of polymers with various structures and topologies. The proposed concept is a response to the growing necessity of implementing the principles of green chemistry in chemical processes, namely, it ensures “less hazardous chemical synthesis” and “inherently safer chemistry for accident prevention” and meets the rule “safer solvents and auxiliaries” and “use of renewable feedstocks”.

## Experimental Section

A material section and experimental details, including reagent specification, analysis, and reaction procedures, are described in the Supporting Information.

## Results and Discussion

### Selection of the ATRP Technique and Examination of the Effect of the Ligand on Polymerization of *n*BA in Cyrene

Initially, the polymerization of *n*BA and *t*BA in *N*,*N*-dimethylformamide (DMF) was conducted to assess the comparative efficiency of using conventional harmful organic solvents versus their naturally derived, nontoxic counterparts, Cyrene and Cygnet 0.0, as the reaction medium for SARA ATRP. In these model polymerizations, we employed ethyl α-bromoisobutyrate (EBiB) as the ATRP initiator, copper wire as both a reducing agent and supplementary activator, and a catalytic complex consisting of copper(II) bromide, along with tris(2-pyridylmethyl)amine (TPMA) as the ligand, which is commonly used in polymerization of acrylates^34,60^ (Table S1). Polymerizations were carried out at [monomer]_0_/[EBiB]_0_/[Cu^II^Br_2_/TPMA]_0_ = 80–120/1/0.036 initial molar ratios. The polymerizations in DMF were characterized by a controlled process, as evidenced by a linear dependence of ln([M]_0_/[M]) on time and a linear increase in *M*_n_ with the monomer consumed. This linear growth continued until reaching approximately 80% monomer conversion after 2.5 h for *n*BA and 1 h for *t*BA polymerization, respectively (Figures S1, S2 and S3). In contrast, the polymerization of *n*BA in Cyrene under the molar ratios [*n*BA]_0_/[EBiB]_0_/[Cu^II^Br_2_/TPMA]_0_ = 80/1/0.036 lasted approximately 100 h to achieve a final monomer conversion of 84% (Table S2, entry 1, Figure S4). The accessible surface area of the initial copper wire source was expected to directly influence the reaction rate.^61^ As the polymer grows in the reaction mixture, it can potentially cover the reducing agent in the form of copper wire. This coverage reduces the active surface area of the copper wire and, consequently, lowers the reduction efficiency of the catalyst (comproportionation). To mitigate this effect, the synthesis was conducted by introducing copper wire in four portions at half-hour intervals (Table S2, entry 2). This treatment significantly reduced the polymerization time by half, resulting in a conversion rate exceeding 90% and yielding a polymer with a narrower molecular weight distribution (Figures S6 and S7b). However, the initiation efficiency was approximately 200%, indicating the contribution of chain transfer on other chemicals in the reaction mixture. Reducing the targeted degree of polymerization to 40 allowed us to achieve a monomer conversion close to 100%. Nonetheless, this approach extended the duration of the polymerization to several days (Table S2, entry 3, Figures S4 and S7c). Increasing the catalyst concentration did not accelerate the polymerization; instead, it exhibited phenomena similar to those observed in Cu-catalyzed ATRP. When comparing polymerizations carried out at catalyst concentrations of 600 and 300 ppm, we observed a slower propagation, and the final product had a broader molecular weight distribution at lower catalyst loadings (compare entries 1 and 4 in Table S2, Figures S5 and S7d).^62,63^

The use of Cyrene was also explored for other ATRP techniques to expedite polymerization while upholding the controlled synthesis (Table S2, entries 5–7). Another technique governed by chemical compounds, wherein catalyst reduction happens through electron transfer from the chemical reducing agent without auxiliary activation as seen in SARA ATRP, is ARGET ATRP. When ascorbic acid was employed as the reducing agent, it led to a monomer conversion of approximately 44% in just 2 h, after which the polymerization ceased (Table S2, entry 5, Figure S6). Despite achieving a high monomer conversion, only dimers and trimers with a molecular mass of around 400 g/mol were obtained (Figure S7e), resulting in an initiation efficiency of 600%. This suggests that in Cyrene, when ascorbic acid is present, chain transfer occurs with reagents such as the monomer or Cyrene or ascorbic acid, and termination of propagating radicals takes place shortly after adding 2 or 3 mers to the growing polymer chains. This phenomenon may be attributed to the potential formation of hemiketals/ketals through the reaction between the hydroxyl groups of ascorbic acid and the ketone group in the presence of strong acid such as hydrobromic acid formed during the reduction of copper(II) bromide by ascorbic acid (Scheme S1).^64^ Given the varying reactivity of hydroxyl groups, it is worth noting that ascorbic acid contains one secondary group, which is typically more reactive than the primary groups. This characteristic increases the likelihood of the observed phenomenon.

Among the various ATRP methods, ATRP controlled by an adjusted electrochemical potential (*E*_app_) or electric current (*I*_app_) stands out for its potential to eliminate the need for chemical reducing agents. This externally controlled technique allows for exceptionally precise control over polymer composition and a faster polymerization rate, depending on the chosen conditions.^33,65,66^ Consequently, we conducted the polymerization of *n*BA using electrochemically mediated ATRP (*e*ATRP) (Table S2, entry 7).

However, the use of *e*ATRP did not lead to an improvement in the polymerization rate (Figure S6a) when compared to SARA ATRP. Additionally, the final polymeric product exhibited a slightly higher dispersity (Figure S6b and Figure S7f). We also explored simplified electrochemically mediated ATRP (*se*ATRP, Table S2, entry 6), which yielded results similar to those obtained with *e*ATRP. It is worth noting that copper(II) bromide can potentially react with ketones to produce the corresponding α-bromo ketones, which precipitated in the reaction mixture.^67^ Hence, we hypothesize that Cyrene consumes the copper-based catalyst, which may explain the faster polymerization rate observed in SARA ATRP, where Cu wire serves as an additional source of copper. This phenomenon was checked by the cyclic voltammetry behavior of the copper catalyst in DMF and Cyrene (Figure S12). In DMF, a typical cyclic voltammetry response was observed for Cu^II^ species, characterized by a distinct peak couple, which confirmed the reversible nature of the reduction and oxidation processes of the copper-based complex.^68,69^ The separation in peak potentials (123 mV) indicated a quasi-reversible process. However, when the copper catalyst was used in Cyrene at the same concentration as in DMF, it exhibited a significantly smaller reduction current, and no associated anodic peak corresponding to the oxidation of reduced species was observed. These results indicate that Cyrene may lead to some side reactions with copper, resulting in a significantly lower concentration of copper-based catalyst in the reaction system compared to DMF at the same initial concentration. As a consequence, the polymerization process becomes less efficient in Cyrene.

**Figure 1:**
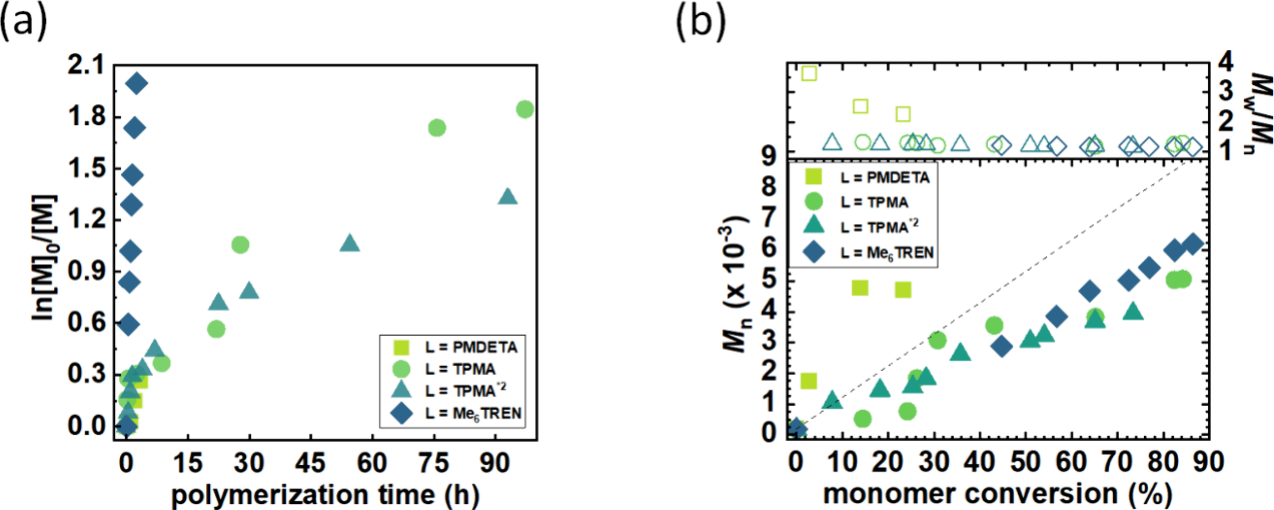
Effect of type of ligand in the catalytic complex on *n*BA polymerization in Cyrene. (a) First-order kinetics plots of monomer conversion vs polymerization time and (b) *M*_n_ and *M*_w_/*M*_n_ vs monomer conversion (Table 1, entries 1–4).

Considering the potential consumption of the copper(II) bromide-based catalyst by Cyrene, alternative ligands were employed to enhance the stability of copper(II) in its oxidized state. Typically, a higher ratio of β^II^/β^I^ (representing the stability constants of Cu^II^ and Cu^I^ complexes with the specific ligand, respectively), along with the equilibrium constant for atom transfer (*K*_ATRP_ = *k*_a_/*k*_d_, *k*_a_ representing the activation rate constant, *k*_d_ representing the deactivation rate constant), signifies the formation of more reducing copper(I) complexes. This, in turn, leads to enhanced ligand stabilization of the copper(II) oxidation state.^70^ To explore the potential for achieving better-controlled polymerization in Cyrene using different ligands, we conducted polymerization reactions with a copper-based catalyst containing various ligands: *N*,*N*,*N*′,*N*″,*N*″-pentamethyldiethylenetriamine (PMDETA), 1-(4-methoxy-3,5-dimethylpyridin-2-yl)-*N*-((4-methoxy-3,5-dimethylpyridin-2-yl)methyl)-*N*-(pyridin-2-ylmethyl)methanamine (TPMA^*2^), and tris[2-(dimethylamino)ethyl]amine (Me_6_TREN). The catalyst complexes stabilized by these ligands were characterized by their β^II^/β^I^ and *K*_ATRP_ values, which are as follows: Me_6_TREN> TPMA^*2^ > TPMA> PMDETA. The polymerization catalyzed by Cu^II^Br_2_/PMDETA (Table 1, entry 1) stopped after reaching approximately 25% conversion (Figure 1) of *n*BA and resulted in a polymer with a high dispersity (Figure S11a). On the other hand, the TPMA*2-containing catalytic complex (Table 1, entry 3) exhibited a behavior similar to that of the one containing TPMA. Interestingly, polymerization with Cu^II^Br_2_/Me_6_TREN (Table 1, entry 4) was significantly faster, approximately 40 times, and yielded a polymer with low dispersity (*M*_w_/*M*_n_ = 1.15, Figure S11c), proceeding similarly to the synthesis carried out in DMF. Moreover, it reduced the initiation efficiency closer to 100% compared to the other catalytic complexes. Compared to Cu^II^Br_2_/TPMA, the Cu^II^Br_2_/Me_6_TREN catalytic complex exhibited a higher cathodic current on the cyclic voltammogram at the same initial catalyst concentration (Figure S12 and S13). This suggests a more stable deactivator form of the copper catalyst when Me_6_TREN is used as a ligand. As a result, a higher concentration of the catalyst is present in the reaction mixture, leading to more efficient polymerizations.

**Table 1:** Polymerization of *n*BA in Cyrene by SARA ATRP Catalyzed by Various Types of Catalytic Complexes^a^

entry	DP_target_	[Cu/L]_0_ [ppm]	L	*t* [h]	conv^b^ [%]	*k*_p_^app^^b^	*M*_n,theo_^c^	*M*_n,app_^d^	*M*_w_/*M*_n_^d^	*I*_eff_^e^ [%]
1	80	300	PMDETA	3.50	23	0.061	2,600	4,700	2.26	55
2^f^	80	300	TPMA	97.20	65	0.022	8,800	5,100	1.28	174
3	80	300	TPMA^*2^	93.00	73	0.017	7,700	4,000	1.17	195
4	80	300	Me_6_TREN	2.50	86	0.896	9,100	6,200	1.15	146
5^g^	80	300	Me_6_TREN	1.50	83	1.157	8,700	6,700	1.13	130
6	80	200	Me_6_TREN	8.40	66	0.164	6,900	4,300	1.11	163
7	80	100	Me_6_TREN	6.00	33	0.060	3,600	2,400	1.23	150
8	200	300	Me_6_TREN	9.00	70	0.181	18,200	13,200	1.23	141
9	600	300	Me_6_TREN	2.25	46	0.310	35,700	24,900	1.38	143

a General reaction conditions: *T* = 50 °C; *V*_tot_ = 4 mL; argon atmosphere; [*n*BA]_0_ = 50% v/v; SARA ATRP with copper wire: *d* = 0.1 cm, *l* = 4 cm; *S*/*V* = 0.318 cm^–1^ for entries 1–4, 6–9; [*n*BA]_0_ = 3.46 M.

b Monomer conversion and apparent rate constant of propagation (*k*_p_^app^) were determined by NMR.

c *M*_n,theo_ = ([*n*BA]_0_/[initiator]_0_) × conversion × *M*_*n*BA_ + *M*_EBiB_.

d Apparent *M*_n_ and *M*_w_/*M*_n_ were determined by GPC.

e Initiation efficiency, *I*_eff_ = (*M*_n,theo_/*M*_n,app_) × 100%.

f Reaction results presented also in Table S2, entry 1.

g SARA ATRP with copper wire: *d* = 0.1 cm, *l* = 8 cm, 4 copper wires 2 cm long were introduced into the reaction mixture at appropriate time intervals: 0, 0.5, 1, 1.5 h; *S*/*V* = 0.632 cm^–1^.

Furthermore, introducing copper wire in four portions at half-hour intervals (Table 1, entry 5) slightly improved the polymerization rate and initiation efficiency (Figure S8), although not as significantly as when using a TPMA-containing catalytic complex. These results support our hypothesis that a ligand capable of better stabilizing the copper(II) oxidation state substantially reduced copper consumption by Cyrene. The phenomenon is not entirely avoided, as indicated by the results of polymerization experiments influenced by the catalyst. When the catalyst loading was reduced to as low as 200 ppm (Table 1, entry 6), polymerization was halted at 70% *n*BA conversion (Figure S9). Conversely, when using only 100 ppm catalyst (Table 1, entry 7), the polymerization proceeded significantly slower.

We also explored the ability to attain a range of molecular weights in Cyrene as a solvent. By aiming for DP_target_ values ranging from 80 to 600, we successfully produced polymers with molecular weights ranging from 6200 to 25,000 g mol^–1^, all with narrow dispersities (Table 1, entries 4, 8, and 9, Figure S10). When we targeted a lower DP (Table 1, entry 4), we observed a high conversion of *n*BA and a lower dispersity in the final product. As we moved toward higher DP_n_ values by reducing the initiator concentration (and consequently lowering the concentration of propagating chains), we experienced longer polymerization times, reduced yields, and broader molecular weight distributions (Table 1, entries 8 and 9, Figure S10, Figure S11g,h). The higher molar masses required extended reaction times, rendering the polymerization more susceptible to side reactions.

### Expanding the Scope of SARA ATRP in Cyrene

We employed the optimized reaction conditions, namely, the polymerization under the molar ratios [monomer]_0_/[initiator]_0_/[Cu^II^Br_2_/Me_6_TREN]_0_ = 80/1/0.024 to polymerize various hydrophobic monomers, many of which had not previously been polymerized in Cyrene.^56^ In addition to *n*BA, we explored the polymerization of *tert*-butyl acrylate (*t*BA), 2-hydroxyethyl acrylate (HEA), oligo(ethylene glycol) methyl ether acrylate (OEGA_480_), methyl methacrylate (MMA), 2-hydroxyethyl methacrylate (HEMA), poly(ethylene glycol) methyl ether methacrylate (OEGMA_300_), glycidyl methacrylate (GMA), and 2-(dimethylamino)ethyl methacrylate (DMAEMA) (Table S3, Figures S14–S16). The polymerization of *t*BA proceeded similarly to that of *n*BA, yielding a final polymer product with low dispersity (*M*_w_/*M*_n_ = 1.15) in a controlled manner. However, the synthesis of PHEA resulted in a 22% monomer conversion with *M*_w_/*M*_n_ = 1.36. The polymerization of OEGA_480_ required a significantly longer time but eventually achieved high monomer conversion and produced a polymer with low dispersity (*M*_w_/*M*_n_ = 1.23). In the case of all methacrylates, polymerization resulted in high conversions, reaching up to 89%, with good control over *M*_n_, and relatively low dispersities (Table S3, entries 5–8). Merely in the case of MMA polymerization (Table S3, entry 5), the molecular weight ceased to increase, reaching approximately 1700 g/mol (Figure S15). This occurred despite the monomer conversion continuing up to 79%, which led to a significant overestimation of the initiation efficiency. This phenomenon can be attributed to the generation of additional radicals, aside from those originating from the ATRP initiator, within the reaction mixture due to chain transfer reactions involving other components of the mixture. Interestingly, this phenomenon was observed exclusively during the polymerization of MMA in Cyrene, suggesting that MMA side chains might play a role in the generation of these additional radicals in the mixture. The structures of the final polymers were confirmed by ^1^H NMR analysis. All the spectra are presented in the Supporting Information in Section S10 (Spectroscopic Characterization of the Prepared Polymers).

### Chain Extension in Cyrene

**Figure 2:**
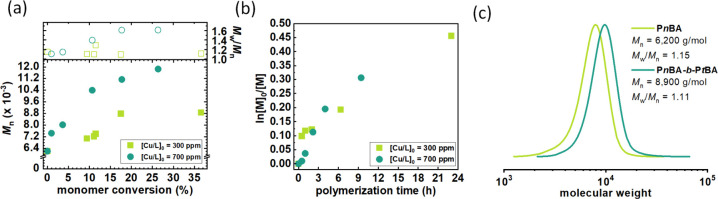
Chain extension experiments of P*n*BA by P*t*BA in Cyrene *via* SARA ATRP varying catalyst concentration. (a) First-order kinetics plots of monomer conversion vs polymerization time and (b) *M*_n_ and *M*_w_/*M*_n_ vs monomer conversion. (c) GPC traces of P*n*BA before and after chain extension by P*t*BA using 300 ppm (Table S4).

To verify the end-group fidelity of the obtained polymers, we proceeded *via* sequential *t*BA addition. Two chain extension experiments were performed varying the catalyst concentration from 300 to 700 ppm (Table S4, Figure 2, Figure S17). Increasing the catalyst loading, the polymerization rate was slightly increased, while the final polymer product characterized by higher dispersity was received. This is probably related to more side reactions of the Cyrene catalyst using a higher concentration of copper in the reaction system. The polymerization with 300 ppm provided the final block copolymer with retained low dispersity of the first polymer block. The structure of the final copolymer was confirmed by ^1^H NMR analysis (Figure S31).

### Polymer Architecture in Cyrene

Since Cyrene has not been previously explored as a polymerization medium for synthesizing polymers with diverse architectures, this study conducted the synthesis of branched polymers using naturally derived cores, namely, riboflavin (vitamin B_2_), troxerutin, and β-cyclodextrin (Table 2, Scheme S2).

**Table 2:** Syntheses of Branched P*n*BA in Cyrene as a Green, Fully Biodegradable Solvent *via* the SARA ATRP Technique^a^

entry	initiator	*t* [h]	conv^b^ [%]	*k*_p_^app^^b^	*M*_n,theo_^c^	*M*_n,app_^d^	*M*_w_**/***M*_n_^d^
1	RF-Br_2_	6.50	79	0.226	16,300	13,800	1.24
2	Trox-Br_10_	4.00	84	0.385	86,000	35,800	1.37
3	β-CD-Br_15_	4.50	83	0.385	127,600	29,300	1.54

a General reaction conditions: *T* = 50 °C; *V*_tot_ = 4 mL; argon atmosphere; [*n*BA]_0_ = 50% v/v; entry 1: [*n*BA]_0_ = 3.49 M, entry 1: [*n*BA]_0_ = 3.50 M, [RF-Br_2_] = 0.024 M calculated per 2 Br initiation sites, entry 2: [Trox-Br_10_] = 0.024 M calculated per 10 Br initiation sites, and entry 3: [*n*BA]_0_ = 3.47 M [β-CD-Br_15_] = 0.024 M calculated per 15 Br initiation sites; [*n*BA]_0_/[initiator]_0_/[Cu^II^Br_2_/Me_6_TREN]_0_ = 80 (per side chain/arm)/1/0.024 for all entries. SARA ATRP with copper wire: *d* = 0.1 cm, *l* = 4 cm.

b Monomer conversion and apparent rate constant of propagation (*k*_p_^app^) were determined by NMR.

c *M*_n,theo_ = ([*n*BA]_0_/[initiator]_0_) × conversion × *M*_Monomer_ + *M*_Initiator_.

d Apparent *M*_n_ and *M*_w_/*M*_n_ were determined by GPC.

**Figure 3:**
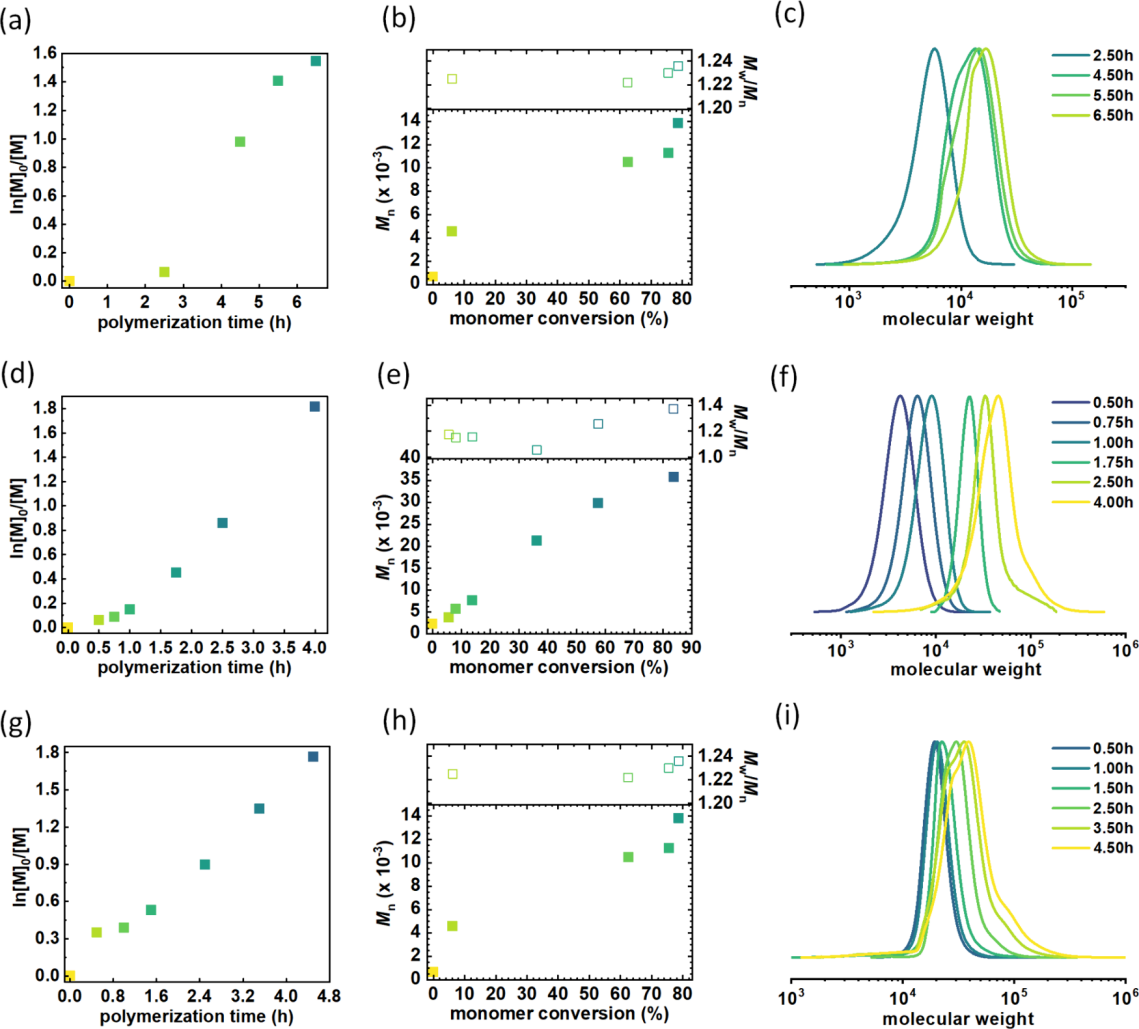
(a, d, g) First-order kinetics plots of monomer conversion vs polymerization time, (b, e, h) *M*_n_ and *M*_w_/*M*_n_ vs monomer conversion, and (c, f, i) GPC traces of polymers synthesized by polymerization of *n*BA *via* SARA ATRP in Cyrene from functionalized riboflavin (Table 2, entry 1), troxerutin (Table 2, entry 2), and β-cyclodextrin (Table 2, entry 3).

These polymerizations resulted in polymers with 2, 10, or 15 side chains/star arms, respectively. The syntheses were well-controlled. Specifically, linear pseudo-first-order kinetics plots indicated a constant radical flux within the system (Figure 3a,d,g). Additionally, the number-average molecular weight of the polymers showed a linear increase with monomer conversion (Figure 3b,e,h). Only a slight induction period was observed when using the riboflavin-derived initiator. This observation is likely associated with the unique character of the unmodified isoalloxazine ring in riboflavin. In addition to the SARA ATRP mechanism facilitated by the presence of a copper wire in the reaction system, the unmodified isoalloxazine ring of riboflavin can act as a reducing agent.^71^ Moreover, it can function as a photocatalyst in the metal-free ATRP technique.^72^ The isoalloxazine ring absorbs visible light across a broad range of wavelengths, allowing it to generate propagating radicals in low concentrations under the influence of visible light, both on the ATRP initiator and within its structure. Therefore, there may be several competing mechanisms catalyzing polymerization that, if acting simultaneously, could impede the initiation of polymerization at the initial synthesis stage. However, considering the rate constants of the mentioned competitive reactions,^71,72^ it becomes evident that SARA ATRP is the dominant mechanism in the tested system. Notably, the final polymer products exhibited relatively low dispersity, with an even *M*_w_/*M*_n_ value as low as 1.24 observed for the polymer with a riboflavin core.

### Kinetics Study of SARA ATRP in Cygnet 0.0

The other bioderived compound is Cygnet 0.0. It can be obtained from the reaction between Cyrene and ethylene glycol, and it is also a promising replacement for toxic polar aprotic solvents.^57,58^ Cygnet 0.0 has not previously been used as a reaction environment of ATRP. It was used as a solvent in two pharmaceutical syntheses, namely, Heck reaction and fluorination. In the case of a fluorination reaction, Cygnet 0.0 showed results similar to those of DMF and superior to those of NMP and acetonitrile. In Heck reaction, it was comparable to NMP and DMSO.^57^ Therefore, we decided to explore Cygnet 0.0 as a solvent for polymerization of various monomers based on previously conducted syntheses in Cyrene.

**Figure 4:**
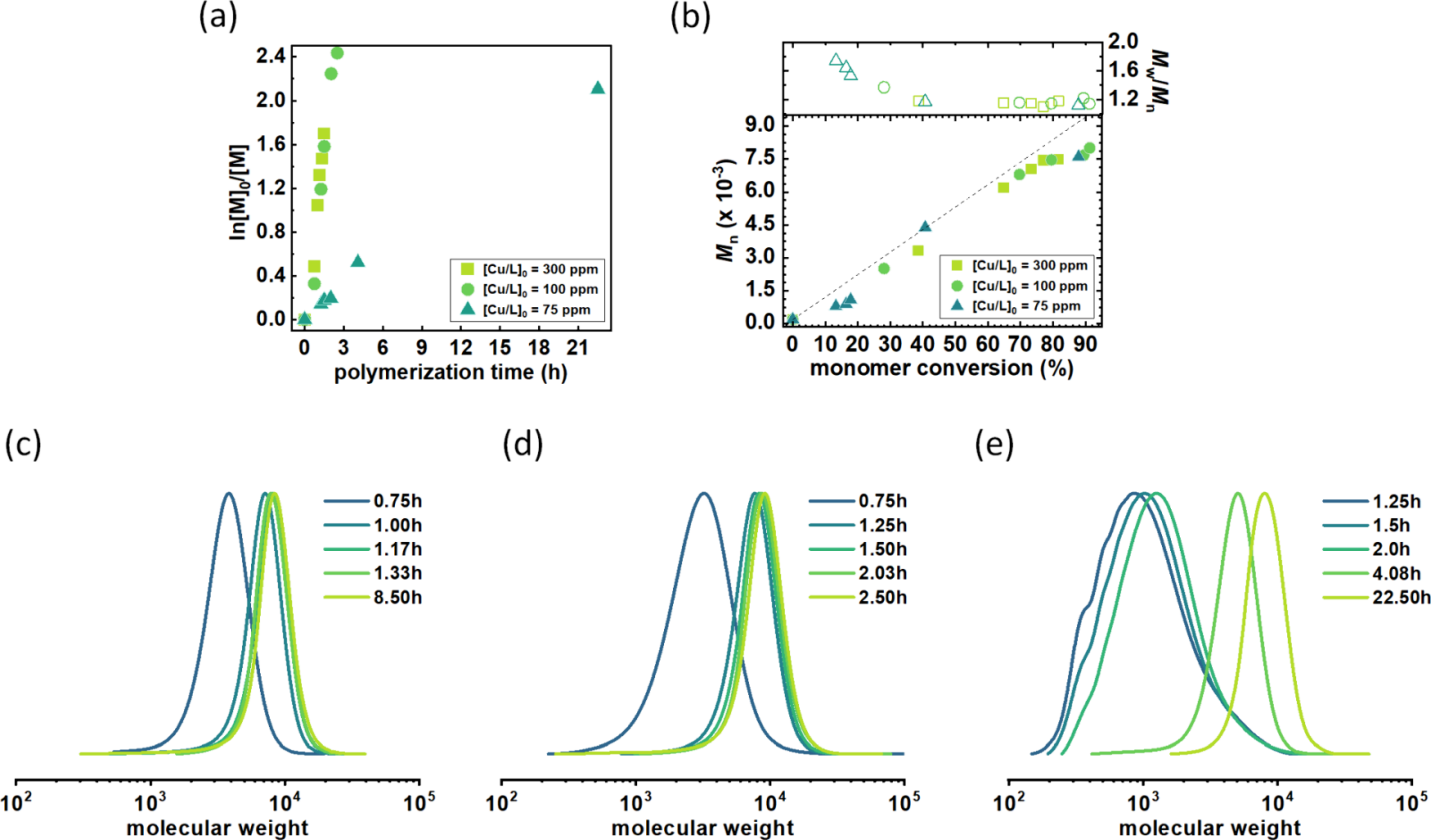
Effect of catalyst concentration on SARA ATRP of *n*BA in Cygnet 0.0. (a) First-order kinetics plots of monomer conversion vs polymerization time and (b) *M*_n_ and *M*_w_/*M*_n_ vs monomer conversion. (c) GPC traces of P*n*BA synthesized using (c) 300 ppm, (d) 100 ppm, and (e) 75 ppm catalyst (Table 3, entries 1, 3, 4, respectively).

Based on the optimized conditions for the polymerization of acrylates in Cyrene, we utilized Cygnet 0.0 as the reaction environment for the polymerization of *n*BA and *t*BA. The molar ratio of all reagents was set at [monomer]_0_/[EBiB]_0_/[Cu^II^Br_2_/TPMA]_0_ = 80/1/0.024 (Table 3, entries 1 and 2). During both syntheses, we observed a linear dependence of ln([M]_0/_[M]) on time, indicating that the polymerization rate followed a first-order reaction with respect to the monomer concentration (Figure 4a, Figure S18a). This was coupled with a linear increase in molecular weights as the monomer converted to polymer (Figure 4b, Figure S18b), and the resulting polymers exhibited monomodal and low dispersity (Figure 4c, Figure S18c). It is noteworthy that the polymerization of *n*BA in Cygnet 0.0 showed closer agreement between the targeted and experimental molecular weights compared to the synthesis in Cyrene under the same reaction conditions (Table 1, entry 4). In Cyrene, a higher concentration of chain transfer processes occurred, resulting in an initiation efficiency of approximately 150%, while in Cygnet 0.0, the initiation efficiency decreased to around 120% or even lower, approaching 100%. The cyclic voltammetry behavior of Cu^II^Br_2_/Me_6_TREN in Cygnet 0.0 showed a reversible peak couple, unlike Cyrene, where there was no anodic peak (Figure S13). This observation suggests that Cygnet 0.0 does not undergo side reactions with the catalyst, in contrast to Cyrene. Consequently, the polymerization process in Cygnet 0.0 is more efficient, resulting in an *I*_eff_ value approaching 100%.

**Table 3:** Polymerization of *n*BA and *t*BA in Cygnet 0.0 *via* SARA ATRP^a^

entry	monomer	[Cu/L]_0_ [ppm]	*t* [h]	conv^b^ [%]	*k*_p_^app^^b^	*M*_n,theo_^c^	*M*_n,app_^d^	*M*_w_**/***M*_n_^d^	*I*_eff_^e^ [%]
1	*n*BA	300	1.50	82	1.074	8,580	7,290	1.18	118
2	*t*BA	300	1.75	99	2.419	10,310	8,950	1.21	115
3	*n*BA	100	2.50	91	0.621	9,550	8,010	1.14	119
4	*n*BA	75	22.5	88	0.095	9,200	7,600	1.12	121
5	*n*BA	50	8.25	no conversion					

a General reaction conditions: *T* = 75 °C; *V*_tot_ = 4 mL; argon atmosphere; [monomer]_0_ = 50% v/v; [*n*BA]_0_ = 3.45 M for entries 1, 3–5, [*t*BA]_0_ = 3.38 M for entry 2, [monomer]_0_/[EBiB]_0_/[Cu^II^Br_2_/Me_6_TREN]_0_ = 80/1/0.024 for entries 1 and 2, [*n*BA]_0_/[EBiB]_0_/[Cu^II^Br_2_/Me_6_TREN]_0_ = 80/1/0.008 for entry 3, [*n*BA]_0_/[EBiB]_0_/ [Cu^II^Br_2_/Me_6_TREN]_0_ = 80/1/0.006 for entry 4, [*n*BA]_0_/[EBiB]_0_/ [Cu^II^Br_2_/Me_6_TREN]_0_ = 80/1/0.004 for entry 5. SARA ATRP with copper wire: *d* = 0.1 cm, *l* = 4 cm.

b Monomer conversion, apparent rate constant of propagation (*k*_p_^app^), and apparent theoretical degree of polymerization of monomer unit (DP_n,theo_) were determined by NMR.

c *M*_n,theo_ = ([monomer]_0_/[EBiB]_0_) × conversion × *M*_Monomer_ + *M*_EBiB_.

d Apparent *M*_n_ and *M*_w_/*M*_n_ were determined by GPC.

e Initiation efficiency, *I*_eff_ = (*M*_n,theo_/*M*_n,app_) × 100%.

Cyclic voltammetry measurements indicated that Cygnet does not form a complex with the catalyst. Therefore, it is possible to reduce the catalyst concentration even further than in Cyrene (Table 3). The polymerization at 100 ppm exhibited an approximately 2-fold lower polymerization rate compared with the polymerization with 300 ppm. The kinetics followed a pseudo-first-order behavior, and there was a linear increase in molecular weights with monomer conversion (Figure 4a,b). This synthesis yielded a final polymer with comparable dispersity (Figure 4d) and initiation efficiency. Even at 75 ppm, the polymerization still exhibited linear kinetics and a narrow molecular weight distribution of the final polymer (Figure 4e), and the apparent molecular weight approached the theoretical value. However, the polymerization rate was 11-fold lower than under the initial reaction conditions. The breaking point was reached at 50 ppm catalyst as the polymerization did not proceed.

### Expanding the Scope of SARA ATRP in Cygnet 0.0

We also explored the possibility of polymerizing a variety of monomers. In addition to *n*BA and *t*BA, we attempted the polymerization of HEA, another acrylate, in Cygnet 0.0 (Table S5, entry 2). The polymerization rate of HEA was 17.5 times higher than of *n*BA at the same catalyst concentration. The synthesis displayed linear kinetics (Figure S19), achieving 92% monomer conversion in just 14 min and ultimately yielding a low dispersity polymer (*M*_w_/*M*_n_ = 1.36, Figure S19c). The polymerizations of methacrylates, namely, MMA and HEMA (Table S5, entries 3 and 4, respectively), exhibited a relatively poor performance. The synthesis of PMMA reached only about 30% monomer conversion, resulting in a low-dispersity final polymer product (*M*_w_/*M*_n_ = 1.38, Figure S20c). On the other hand, the polymerization of HEMA ceased at just around 17% conversion.

### Polymer Architecture in Cygnet 0.0

**Figure 5:**
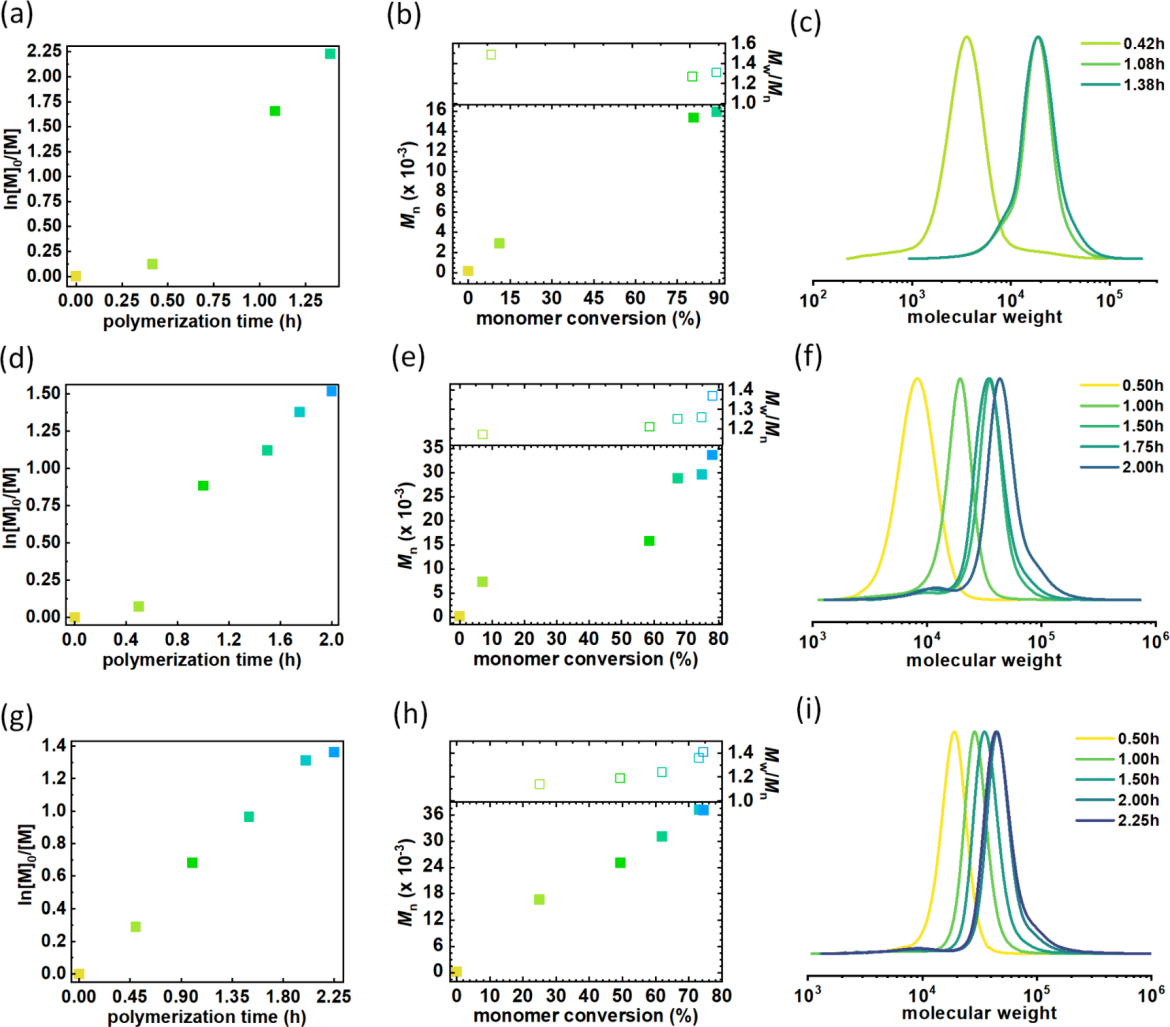
(a, d, g) First-order kinetics plots of monomer conversion vs polymerization time, (b, e, h) *M*_n_ and *M*_w_/*M*_n_ vs monomer conversion, and (c, f, i) GPC traces of branched polymers synthesized by polymerization of *n*BA *via* SARA ATRP in Cyrene from functionalized riboflavin (Table 4, entry 1), troxerutin (Table 4, entry 2), and β-cyclodextrin (Table 4, entry 3).

The synthesis of polymers with diverse architectures was also carried out by incorporating naturally derived cores into polymer structures, namely, riboflavin (vitamin B_2_), troxerutin, and β-cyclodextrin (Table 4, Scheme S2). Similar to the polymerization in Cyrene, these syntheses yielded polymers with 2, 10, or 15 side chains/arms, respectively. These synthesis procedures were well-controlled. Specifically, linear pseudo-first-order kinetics plots indicated a consistent radical flux within the system (Figure 5a,d,g). Additionally, the number-average molecular weight of the polymers exhibited a linear increase with monomer conversion (Figure 5b,e,h). Notably, the final polymer products demonstrated relatively low dispersities, ranging from 1.31 to 1.41.

**Table 4:** Syntheses of Branched P*n*BA in Cygnet 0.0 as a Green, Fully Biodegradable Solvent *via* the SARA ATRP Technique^a^

entry	initiator	*t* [h]	conv^b^ [%]	*k*_p_^app^^b^	*M*_n,theo_^c^	*M*_n,app_^d^	*M*_**w**_**/***M*_n_^d^
1	Rib-Br_2_	1.38	89	1.510	19,000	15,900	1.31
2	Trox-Br_10_	2.00	78	0.761	82,200	33,700	1.37
3	β-CD-Br_15_	2.25	74	0.634	118,700	37,100	1.41

a General reaction conditions: *T* = 75 °C; *V*_tot_ = 3 mL for entry 1, *V*_tot_ = 4 mL for entries 2 and 3; argon atmosphere; [*n*BA]_0_ = 50% v/v; entry 1: [*n*BA]_0_ = 3.48 M, [RF-Br_2_] = 0.0217 M calculated per 2 Br initiation sites, entry 2: [*n*BA]_0_ = 3.48 M, [Trox-Br_10_] = 0.0043 M calculated per 10 Br initiation sites, and entry 3: [*n*BA]_0_ = 3.47 M [β-CD-Br_15_] = 0.0029 M calculated per 15 Br initiation sites; [*n*BA]_0_/[initiator]_0_/[Cu^II^Br_2_/Me_6_TREN]_0_ = 80/1/0.008 for all entries. SARA ATRP with copper wire: *d* = 0.1 cm, *l* = 3 cm for entry 1, *d* = 0.1 cm, *l* = 4 cm for entries 2 and 3.

b Monomer conversion and apparent rate constant of propagation (*k*_p_^app^) were determined by NMR.

c *M*_n,theo_ = ([*n*BA]_0_/[initiator]_0_) × conversion × *M*_Monomer_ + *M*_Initiator_.

d Apparent *M*_n_ and *M*_w_/*M*_n_ were determined by GPC.

### Green Chemistry Metrics

The sustainability of a chemical process, as determined by its adherence to green chemistry principles, is evaluated using green chemistry metrics. These metrics help quantify the efficiency and environmental performance of chemical processes while facilitating the measurement of any changes in performance. One widely accepted measure of a chemical process environmental impact is the environmental factor (*E*-factor),^73,74^ which is defined as the mass ratio of waste generated to the desired product. *E*-factors do not account for recyclable factors such as reused solvents and catalysts, which enhances accuracy but overlooks the energy involved in the recovery process. A higher *E*-factor indicates more waste generation and, consequently, a greater negative environmental impact. The ideal *E*-factor is zero. The syntheses that we conducted demonstrate quite favorable *E*-factor values, ranging from approximately 1.5 to 2.1 (Table S6). These values highlight the relatively small amounts of waste generated in our chemical processes, especially when compared to other industry segments like pharmaceuticals and fine chemicals, where *E*-factors can reach levels as high as 5 to 100.

The remarkable advantage of employing eco-friendly solvents like Cyrene and Cygnet 0.0, as opposed to commonly used toxic solvents such as DMF, is best reflected in the effective mass yield (EMY). The EMY is a measure of the mass of the desired product relative to the mass of all nonenvironmentally friendly materials used in its production.^75^ A higher EMY value indicates a more environmentally favorable synthesis. When most reagents are environmentally friendly, the EMY can exceed 100%. Comparing the polymerization of *n*BA conducted in DMF to that in Cyrene or Cygnet 0.0 (Table 5, Figure S43), a significant difference in EMY becomes apparent. The synthesis in DMF exhibited an EMY of approximately 80%, while using biobased, nontoxic solvents as the reaction environment boosted this value by a remarkable 54-fold. It is noteworthy that the polymerization rate and the quality of the resulting polymer products remain comparable in both the hazardous polar aprotic solvent and its biobased alternatives. The similar quality of these syntheses and the EMY values achieved in Cyrene and Cygnet 0.0 underscore the immense potential of these solvents for creating processes in line with the principles of green chemistry.

**Table 5:** Polymerization of *n*BA in DMF, Cyrene, and Cygnet 0.0 *via* SARA ATRP^a^

									EMY^app^^g^ [%]	
entry	solvent	*t* [h]	conv^b^ [%]	*M*_n,theo_^c^	*M*_n,app_^d^	*M*_w_**/***M*_*n*_^d^	*I*_eff_^e^ [%]	EMY^theo^^f^ [%]	dialysis	precipitation
1	DMF	2.0	79	8,300	9,500	1.10	88	79	78	77
2	Cyrene	3.0	82	8,600	6,400	1.12	135	4,008	3,626	3,811
3	Cygnet 0.0	2.0	88	9,200	8,600	1.12	107	4,299	4,181	2,734

a General reaction conditions: *T* = 50 °C for entries 1 and 2 and *T* = 75 °C for entry 3; *V*_tot_ = 4 mL; argon atmosphere; ligand: TPMA for entry 1, and Me_6_TREN for entries 2 and 3; [*n*BA]_0_ = 50% v/v; [*n*BA]_0_ = 3.45 M; [Cu^II^/L]_0_ = 300 ppm; [*n*BA]_0_/[EBiB]_0_/[Cu^II^Br_2_/ligand]_0_ = 80/1/0.024. SARA ATRP with copper wire: *d* = 0.1 cm, *l* = 4 cm.

b Monomer conversion, apparent rate constant of propagation (*k*_p_^app^), and apparent theoretical degree of polymerization of monomer unit (DP_n,theo_) were determined by NMR.

c *M*_n,theo_ = ([*n*BA]_0_/[EBiB]_0_) × conversion × *M*_*n*BA_ + *M*_EBiB_.

d Apparent *M*_n_ and *M*_w_/*M*_n_ were determined by GPC.

e Initiation efficiency, *I*_eff_ = (*M*_n,theo_/*M*_n,app_) × 100%.

f EMY^theo^, theoretical effective mass yield, defined as the percentage of the mass of the desired product calculated based on monomer conversion calculated from NMR analysis relative to the mass of all nonbenign materials used in its synthesis, i.e., catalyst, ligand, unreacted monomer, and solvent in the case of the synthesis in DMF.

g EMY^app^, apparent effective mass yield, defined as the percentage of the actual mass of weighed polymer after dialysis or precipitation relative to the mass of all nonbenign materials used in its synthesis, i.e., catalyst, ligand, unreacted monomer, and solvent in the case of the synthesis in DMF.

### Catalyst Concentration in the Final Polymer Products

An important consideration for the practical use of the obtained polymers, particularly in fields such as medicine, pharmacy, or various industrial applications, where the purity of the final polymer products plays a crucial role in determining their suitability, is the concentration of transition metals in the product, specifically copper. It is noteworthy that only 100 ppm catalyst was used in the conducted syntheses. The copper content in the resulting polymers was analyzed by atomic absorption spectrometry (AAS) both in DMF and its nontoxic alternatives, Cyrene and Cygnet 0.0, after purification through dialysis or precipitation (Table S7). Upon the recovery of polymers from the DMF-containing reaction mixture, the final products exhibited the highest copper concentration, exceeding 400 ppm. However, when purifying polymers dissolved in Cyrene or Cygnet 0.0, the final products contained only 8 ppm copper. The purification process through dialysis consistently yielded end products with controlled metal concentrations in a green solvent. We attribute the variations in the copper concentration within products obtained through precipitation by appropriate solvent mixtures to differences in the physicochemical properties of the solvents constituting the reaction medium.

## Conclusions

We have gained extensive insights into polymerizations of various monomers using ATRP techniques controlled by both chemical reducing agents and an external stimulus, such as electric current, in Cyrene. The outcomes from kinetics studies and electrochemical characterization of the catalytic complex in this solvent indicated the potential for side reactions occurring in Cyrene, disrupting controlled polymerization. Therefore, the ligand selection is crucial, as Me_6_TREN ensured the most effective polymerization in Cyrene, possibly due to copper(II) bromide being consumed by the solvent, generating the corresponding α-bromoketones. Cyrene serves as an efficient alternative dipolar aprotic solvent for SARA ATRP of both hydrophobic and hydrophilic monomers, providing final products with low dispersity, even as low as 1.15. Additionally, it offers the potential for synthesizing polymer architectures with naturally derived cores like riboflavin, β-cyclodextrin, and troxerutin, significantly broadening the solvent’s application scope.

We have also showed the initial example of ATRP polymerization in Cygnet 0.0, a derivative of Cyrene. In comparison to Cyrene, the share of side reactions between Cygnet 0.0 and the catalyst was significantly minimized, allowing for a reduced concentration of the catalytic complex in the reaction mixture, down to 75 ppm. This enabled the synthesis of polymers with low dispersity at high conversions, even with complex architectures such as brush-like polymers.

The EMY values achieved in Cyrene and Cygnet 0.0 highlight a considerable advantage of these solvents over DMF in creating processes aligned with the principles of green chemistry. The copper residue in the final polymers is several hundred times lower than the permissible daily exposure to orally administered copper in pharmaceuticals. Therefore, the resulting polymeric materials, along with the concept of using benign biobased alternatives instead of harmful protic solvents, hold immense potential for diverse applications, including in the pharmaceutical industry.

## Supporting Information Available

Experimental section, polymerizations results, kinetics investigation, GPC traces, electrochemical characterization of copper-based catalytic in various solvents, ^1^H NMR analysis of synthesized polymers, *E*-factor and effective mass yield (EMY), and atomic absorption spectrometry (AAS) results (PDF)
