# Supplementary material for: Controlled Polymer Synthesis Toward Green Chemistry: Deep Insights into Atom Transfer Radical Polymerization in Biobased Substitutes for Polar Aprotic Solvents
Source: ACS Sustain Chem Eng. 2024 Feb 21;12(12):4933–45. doi: 10.1021/acssuschemeng.3c07993 (PMC10966734; doi:10.1021/acssuschemeng.3c07993)
Supplement: Supplementary file 1 — sc3c07993_si_001.pdf [file sc3c07993_si_001.pdf]

Supporting Information for

Controlled Polymer Synthesis Toward Green Chemistry: Deep Insight into ATRP in Bio-Based Substitutes for Polar Aprotic Solvents

Izabela Zaborniak,[†,‡] *Małgorzata Klamut,*[†,§] *Cicely M. Warne,*[±,ɫ] *Katarzyna Kisiel,*[†] *Martyna Niemiec,*[†] *Paweł Błoniarz,*[†] *Alessandro Pellis,*[*,δ] *Krzysztof Matyjaszewski*[*,‡] *and Paweł Chmielarz,*[*,†,‡]

†Department of Physical Chemistry, Faculty of Chemistry, Rzeszow University of Technology, al. Powstańców Warszawy 6, 35-959 Rzeszów, Poland

‡Department of Chemistry, Carnegie Mellon University, 4400 Fifth Ave., Pittsburgh, PA 15213, United States

§Doctoral School of the Rzeszów University of Technology, al. Powstańców Warszawy 8, 35-959 Rzeszów, Poland

[*] Corresponding author. E-mail: alessandro.pellis@unige.it

[*] Corresponding author. E-mail: km3b@andrew.cmu.edu

[*] Corresponding author. E-mail: p_chmiel@prz.edu.pl

±Institute for Environmental Biotechnology, Department for Agrobiotechnology, University of Natural Resources and Life Sciences, Konrad Lorenz Strasse 20, A-3430 Tulln an der Donau, Austria

[ǂ]ACIB GmbH, Konrad-Lorenz-Strasse 20, 3430 Tulln an der Donau, Austria

[§]Department of Chemistry and Industrial Chemistry, University of Genova, Via Dodecaneso 31, 16146, Genova, Italy.

Number of pages: S1-S7

Number of Figures: Figure S1-S44

Number of Schemes: Scheme S1-S2

Number of Tables: Table S1-S7

Content

S1. Experimental Section 4

S2. Polymerization of acrylates in *N*,*N*-dimethylformamide 17

S3. Polymerization of *n*BA in Cyrene™ using TPMA-containing catalytic complex.............20

S4. Polymerization of *n*BA by SARA ATRP in Cyrene™ using various catalytic complex ...26

S5. Electrochemical characterization of copper-based catalytic complex in Cyrene™ and Cygnet compared to *N*,*N*-dimethylformamide 30

S6. Polymerization of various monomers in Cyrene™ by SARA ATRP 32

S7. Chain extension of P*n*BA by SARA ATRP in Cyrene™ 36

S8. Polymerization of various acrylates and methacrylates *via* SARA ATRP in Cygnet 0.0...38

S9. Synthetic routes for the synthesis of polymers with centrally-located naturally derived substances in Cyrene™ and Cygnet 0.0 and their structures 42

S10. Spectroscopic characterization of the prepared polymers..44

S11. E-factor and effective mass yield (EMY) 60

S12. Determination of copper concentration in final polymer samples by atomic absorption spectrometry (AAS) 62

References 64

S1. Experimental Section

Materials. Cyrene™ was prepared as described in the literature,[1] Cygnet 0.0 was also synthesized in accordance with the literature.[2] Ethyl 2-bromoisobutyrate (EBiB, 98%) were purchased from Sigma Aldrich, ethyl 2-bromo-phenylacetate (EBPA, 97%) was purchased from Acros Organics. Nitric acid (puriss. p.a., reag. ISO, ≥65%) was purchased from Fluka (Honeywell), while hydrogen peroxide (pure p.a., 30%) from Chempur. ATRP initiators for the preparation of branched architectures were prepared according to the procedures described in the papers, namely, synthesis of brominated troxerutin (Trox-Br_{10}) was described in,[3] while brominated riboflavin (RF-Br_2) in,[4] while brominated β-cyclodextrin (β-CD-Br_{15}) in.[5] Copper (II) bromide ($Cu^{II}Br_2$, 99.9%), tris[2-(dimethylamino)ethyl]amine (Me_6TREN, 97%) and *N,N,N',N'',N''*-Pentamethyldiethylenetriamine (PMDETA, 98%) were purchased from Sigma Aldrich. Tris(2-pyridylmethyl) amine (TPMA) synthesis was carried out in adherence to the established published procedure,[6] while 1-(4-methoxy-3,5-dimethylpyridin-2-yl)-*N*-((4-methoxy-3,5-dimethylpyridin-2-yl)methyl)-*N*-(pyridin-2-ylmethyl)methanamine (TPMA^{*2}) in.[7] *n*-Butyl acrylate (*n*BA, 99%), *t*-butyl acrylate (*t*BA, 99%), 2-hydroxyethyl acrylate (HEA, 96%), oligoethylene glycol methyl ether acrylate ($OEGA_{480}$), methyl methacrylate (MMA, ≥99%), 2- hydroxyethyl methacrylate (HEMA, 97%), glycidyl methacrylate (GMA, >99%), 2-(dimethylamino)ethyl methacrylate (DMAEMA, >99%) were purchased from Sigma Aldrich. To eliminate the inhibitor, the material was filtered through a basic alumina column before use. Deuterated solvents like dimethylsulfoxide-d6 (DMSO, 99.8%, 0.03% v/v TMS) and chloroform-d ($CDCl_3$, 99.8%, 0.03% TMS, stabilized by 0.5% w/w Ag foil) were purchased from Deutero.

Analysis. Proton nuclear magnetic resonance (^{1}H NMR) was utilized to analyze the kinetics of polymerizations and investigate the structure of the resulting polymer products. Spectra were

carried in deuterated solvent – DMSO-d_6 and chloroform-*d* using Bruker Avance 500 MHz spectrometer (25°C). Gel permeation chromatography (GPC) was employed to determine both molecular weights (MWs) and molecular weight distributions (MWDs, M_w/M_n, $Đ$), using a Shimadzu (Kyoto, Japan) modular system equipped with a CBM-40 system controller, SIL-20AHT automatic injector, the RID-20A differential refractive-index detector and PSS GRAM combination columns made of stainless steel (V4A); for determination of MWs and MWDs of PHEA, PHEMA, POEGA, PGMA, PDMAEMA the columns set was composed of one precolumn, one 100 Å column and two 3,000 Å columns, the temperature of the columns was maintained at 35 °C using a CTO-20A oven, the eluent was *N*,*N*-dimethylformamide (HPLC grade, with 0.01 M LiCl) and the flow rate was kept at 1 mL/min using a LC-40 pump. While, for determination of MWs and MWDs of P*n*BA, P*t*BA and PMMA the columns set was composed of precolumn, and three Repro-Gel 5 µm columns (500 Å, 10,000 Å and 100,000 Å), the temperature of the columns was maintained at 35°C using a CTO-20A oven, the eluent was tetrahydrofuran (HPLC grade) and the flow rate was kept at 1 mL/min using a LC-40 pump. A molecular weight calibration curve was produced using commercial narrow molecular weight distribution polystyrene standards (PSS Polymer Standards Service, Mainz, Germany). Cyclic voltammetry (CV) measurements were performed using an Autolab model AUT84337 potentiostat running with GPES software in a five-neck electrochemical cell equipped with electrodes as follows: working electrode (WE) – Pt disk (A = 0.071 cm^2, carefully polished with 0.05 µm alumina suspension (Buehler) before every single measurement), and Pt mesh was used for preparative electrolysis (A = ~6 cm^2), reference electrode (RE) – saturated calomel electrode (SCE), equipped with a saturated salt bridge and a Vycor tip, immersed inside a Luggin capillary, counter electrode (CE) – an Al wire (l = 10 cm, d = 1 mm) immersed directly in the reaction mixture. During CV measurement at 75°C and preparative electrolysis at 50°C a condenser was connected to the reaction cell kit and the temperature was

maintained by thermostat (Labo Play ESM-3711-H). AAS analyses were carried out with the use of a Thermo Scientific iCE 3500 spectrometer equipped with a copper hollow cathode lamp as the radiation source and a deuterium lamp providing background correction. The measurements were conducted at the wavelength of 324.8 nm and 75% power of the lamp. The flame ionization technique was used (air-acetylene flame). Mineralization of the polymers was performed using the Milestone Start D Microwave Digestion System.

General procedure for the synthesis of P*n*BA in Cyrene™ as green solvent *via* SARA or ARGET ATRP

A series of SARA ATRP reactions were carried out with different target degrees of polymerization (DP_{target}) and catalyst complex concentration (Cu/L ppm). Stock solutions of catalyst were prepared by mixing $Cu^{II}Br_2$ with ligand (TPMA or PMDETA or TPMA^{*2} or Me_6TREN) in a molar ratio of catalyst to ligand 1:2 for TPMA or PMDETA or TPMA^{*2} and 1:6 for Me_6TREN and dissolving in DMF. The mixture of *n*BA (2.00 mL, 13.95 mmol), $Cu^{II}Br_2$/TPMA stock solution (84 µL or 167 µL of 0.05 M in DMF), EBiB (26.0 µL or 51 µL, 0.17 mmol) and Cyrene™ (1.92 mL or 1.83 mL) was added to a 10 mL Schlenk flask equipped with a magnetic stirrer bar. Next, Cu^0 wire (l = 4 cm or 9 cm, d = 1 mm) previously activated with HCl and washed with water, acetone or THF was placed in a rubber septum, which was then inserted into a Schlenk flask containing the reaction mixture. The flask was placed in an oil bath heated to 50°C and then the mixture was degassed for 10 – 15 min under an argon atmosphere. Cu^0 wire was added to the reaction mixture to initiate the polymerization. Samples were withdrawn periodically to follow monomer conversion using 1H NMR analysis and to check M_n and M_w/M_n of the polymers by GPC analysis. Before GPC analysis the polymer samples were dissolved in THF (1 mL) + toluene (10 µL) as an external standard in the mobile phase and passed through a neutral alumina column with a 0.22 µm syringe filter in

order to remove the catalyst. The polymerization was stopped by opening the flask and exposing the catalyst to air.

In ARGET ATRP the mixture of *n*BA (2.00 mL, 13.95 mmol), $Cu^{II}Br_2$/TPMA stock solution (84 µL of 0.05 M in DMF), EBiB (51 µL, 0.35 mmol) and CYRENE™ (1.85 mL) was added to a 10 mL Schlenk flask equipped with a magnetic stirrer bar. The flask was placed in an oil bath heated to 50°C and then the mixture was degassed for 10 – 15 min under argon atmosphere. Next, AsAc solution in DMF was injected by a syringe pump ($[AsAc]/[Cu^{II}Br_2]_0 = 1.5$) to start the reaction. Samples were withdrawn periodically to follow monomer conversion using ^{1}H NMR analysis and to check M_n and M_w/M_n of the polymers by GPC analysis. Before GPC analysis the polymer samples were dissolved in THF (1 mL) + toluene (10 µL) as an external standard in the mobile phase and passed through a neutral alumina column with a 0.22 µm syringe filter in order to remove the catalyst. The polymerization was stopped by opening the flask and exposing the reaction mixture to air.

General procedure for the synthesis of P*n*BA in Cyrene™ as green solvent *via se*ATRP/ *e*ATRP

TBAP (0.5471 g, 1.6 mmol), *n*BA (4 mL, 27.89 mmol), CYRENE™ (3.8 mL) and 167 µL $Cu^{II}Br_2$/TPMA stock solution (0.05 M in DMF) were added into a five-neck electrochemical cell. The flask was placed in an oil bath at 50°C under a slow Ar purge. Before the electrolysis, the CV was recorded with Pt disk working electrode (WE), SCE reference electrode (RE), and Al wire/Pt mesh counter electrode (CE) to determine the applied potential ($E_{app} = E_{pc}$-40 mV) used subsequently during *se*ATRP or *e*ATRP procedure. In *se*ATRP Al wire was used as CE directly immersed in the reaction solution, while in *e*ATRP Pt mesh CE was prepared using a glass frit and

a salt bridge made of Tylose gel saturated with $TBAPF_6$ to separate the cathodic and anodic compartments. Then, EBiB (51 µL, 0.35 mmol) was injected into the reaction solution and the CV was recorded, after that the WE was replaced to Pt mesh and immersed in reaction mixture. The mixture was degassed for 15 min and after that, the selected potential was applied using the constant potential electrolysis method. Samples were withdrawn periodically to follow monomer conversion using ^{1}H NMR analysis and to check M_n and M_w/M_n of the polymers by GPC analysis. Before GPC analysis the polymer samples were dissolved in THF (1 mL) + toluene (10 µL) as an external standard in the mobile phase and passed through a neutral alumina column with a 0.22 µm syringe filter in order to remove the catalyst.

General procedure for the synthesis of P*n*BA in CYRENE™ as green solvent *via* SARA ATRP.

A series of SARA ATRP reactions were carried out with different target degrees of polymerization (DP_{target}) and catalyst complex concentration (Cu/L ppm) and ligands. The mixture of *n*BA (2.00 mL, 13.95 mmol), Cu^{II}/Me_6TREN (84 µL or 83 µL or 56 µL or 28 µL of 0.05 M in DMF) for Table 2 entries 8-9 and 12-15, $Cu^{II}/TPMA^*2$ (84 µL of 0.05 M in DMF) for Table 2 entry 10, Cu^{II}/PMDETA (84 µL of 0.05 M in DMF) for Table 2 entry 11, EBiB (26.0 µL or 10.3 µL or 3.40 µL, 0.17 mmol or 0.07 mmol or 0.02 mmol) and CYRENE™ (1.92 mL or 1.83 mL) was added to a 10 mL Schlenk flask equipped with a magnetic stirrer bar. Next, Cu^0 wire (l = 4 cm or 8 cm, d = 1 mm) previously activated with HCl and washed with water, acetone or THF was placed in a rubber septum, which was then inserted into a Schlenk flask containing the reaction mixture. The flask was placed in an oil bath heated to 50 °C and then the mixture was degassed for 10 – 15 min under argon atmosphere. Cu^0 wire was added to the reaction mixture to initiate the polymerization. Samples were withdrawn periodically to follow monomer conversion using ^{1}H NMR analysis and

to check M_n and M_w/M_n of the polymers by GPC analysis. Before GPC analysis the polymer samples were dissolved in THF (1 mL) + toluene (10 μL) as an external standard in the mobile phase and passed through a neutral alumina column with a 0.22 μm syringe filter in order to remove the catalyst. The polymerization was stopped by opening the flask and exposing the catalyst to air.

General procedure for the synthesis of different acrylates in CYRENE™ as green solvent *via* SARA ATRP.

A series of SARA ATRP reactions were carried out with different types of acrylates. The mixture of *t*BA (2.00 mL, 13.74 mmol) or HEA (2.00 mL, 13.45 mmol) or $OEGA_{480}$ (1.2 mL 2.75 mmol) and $Cu^{II}Br_2/Me_6TREN$ stock solution (82 μL or 105 μL or 16 μL of 0.05 M in DMF), EBiB (25.0 μL or 32.0 μL, or 5.0 μL 0.17 mmol) and CYRENE™ (1.90 mL or 1.89 mL or 2.77 mL) was added to a 10 mL Schlenk flask equipped with a magnetic stirrer bar. Next, Cu^0 wire (l = 4 cm or 9 cm, d = 1 mm) previously activated with HCl and washed with water, acetone or THF was placed in a rubber septum, which was then inserted into a Schlenck flask containing the reaction mixture. The flask was placed in an oil bath heated to 50 °C and then the mixture was degassed for 10 – 15 min under argon atmosphere. Cu^0 wire was added to the reaction mixture to initiate the polymerization. Samples were withdrawn periodically to follow monomer conversion using ^1H NMR analysis and to check M_n and M_w/M_n of the polymers by GPC analysis. Before GPC analysis the polymer samples were dissolved in mobile phase containing THF (1 mL) + toluene (10 μL) (for entry 2) or DMF (1 mL + 10 mM LiCl) + toluene (10 μL) (for entries 3 – 4) as an external standard, and passed through a neutral alumina column with 0.22 μm syringe filter in order to remove the catalyst. The polymerization was stopped by opening the flask and exposing the catalyst to air.

General procedure for the synthesis of different methacrylates in CYRENE™ as green solvent *via* SARA ATRP.

A series of SARA ATRP reactions were carried out with various types of methacrylates. The solution of one of the following monomers, like MMA (2.00 mL, 18.76 mmol), HEMA (2.00 mL, 16.45 mmol), $OEGMA_{300}$ (1.20 mL, 4.21 mmol), PGMA (2.00 mL, 14.64 mmol), DMAEMA (2.00 mL, 11.85 mmol) or NIPAM (0.789g, 6.97 mmol) was mixed with $Cu^{II}Br_2/Me_6TREN$ stock solution (113 µL or 99 µL or 25 µL or 88 µL or 71 µL or 41 µL of 0.05 M in DMF), EBPA (41.0 µL 0.23 mmol or 36.0 µL 0.21 mmol or 9.0 µL 0.05 mmol or 32.0 µL 0.18 mmol or 26.0 µL 0.15 mmol or 12.8 µL 0.08 mmol) and CYRENE™ (1.88 mL or 1.91 mL or 1.93 mL or 3.17 mL) and then was added to a 10 mL Schlenk flask already fitted with a magnetic stirrer bar. Next, Cu^0 wire (l = 4 cm or 9 cm, d = 1 mm) previously activated with HCl and washed with water, acetone or THF was placed in a rubber septum, which was then inserted into a Schlenck flask containing the reaction mixture. The Schlenk flask, along with its contents, was positioned within a 50°C oil bath. Subsequently, the mixture underwent a degassing process for a duration of 10-15 minutes under argon atmosphere. Cu^0 wire was added to the reaction mixture to initiate the polymerization. Samples were withdrawn periodically to follow monomer conversion using ^1H NMR analysis and to check M_n and M_w/M_n of the polymers by GPC analysis. Before GPC analysis the polymer samples were dissolved in mobile phase containing THF (1 mL) + toluene (10 µL) (for entry 5) or DMF (1 mL + 10 mM LiCl) + toluene (10 µL) (for entries 6 – 10) as an external standard, and passed through a neutral alumina column with 0.22 µm syringe filter in order to remove the catalyst. The polymerization was stopped by opening the flask and exposing the catalyst to air.

General procedure for chain extension of P*n*BA by P*n*BA as a second block in CYRENE™ as green solvent *via* SARA ATRP.

tBA (0.9 mL, 6.00 mmol), $Cu^{II}Br_2$/Me_6TREN stock solution (36 µL or 85 of 0.05 M in DMF), and CYRENE™ (2.95 mL or 2.91 mL), were added to a 10 mL Schlenk flask equipped with a magnetic stirrer bar. Next, Cu^0 wire (l = 4 cm, d = 1 mm) previously activated with HCl and washed with water, acetone or THF was placed in a rubber septum, which was then inserted into a Schlenk flask containing the reaction mixture. The flask was placed in an oil bath heated to 50°C and then the mixture was degassed for 10 – 15 min under argon atmosphere. Cu^0 wire was added to the reaction mixture to initiate the polymerization. Samples were withdrawn periodically to follow monomer conversion using ^{1}H NMR analysis and to check M_n and M_w/M_n of the polymers by GPC analysis. Before GPC analysis the polymer samples were dissolved in THF (1 mL) + toluene (10 µL) as an external standard in the mobile phase and passed through a neutral alumina column with a 0.22 µm syringe filter in order to remove the catalyst. The polymerization was stopped by opening the flask and exposing the catalyst to air.

General procedure for the polymerization of *n*BA from Trox-Br_{10}, Rib-Br_2 and β-CD-Br_{15} in CYRENE™ as green solvent *via* SARA ATRP.

nBA (2mL, 13.97 mmol), initiator: Trox-Br_{10} (39.0 mg, 0.02 mmol) or Rib-Br_2 (59.0 mg, 0.09 mmol) or β-CD-Br_{15} (39.0 mg, 0.01 mmol), $Cu^{II}Br_2$/Me_6TREN stock solution (84 µL or 83 µL of 0.05 M in DMF), and CYRENE™ (1.91 mL or 1,93 mL), was added to a 10 mL Schlenk flask equipped with a magnetic stirrer bar. Next, Cu^0 wire (l = 4 cm or 9 cm, d = 1 mm) previously activated with HCl and washed with water, acetone or THF was placed in a rubber septum, which was then inserted into a Schlenk flask containing the reaction mixture. The flask was placed in an oil bath heated to 50°C and then the mixture was degassed for 10 – 15 min under argon atmosphere. Cu^0 wire was added to the reaction mixture to initiate the polymerization. Samples were withdrawn periodically to follow monomer conversion using ^{1}H NMR analysis and to check M_n and M_w/M_n

of the polymers by GPC analysis. Before GPC analysis the polymer samples were dissolved in THF (1 mL) + toluene (10 μL) as an external standard in the mobile phase and passed through a neutral alumina column with a 0.22 μm syringe filter in order to remove the catalyst. The polymerization was stopped by opening the flask and exposing the catalyst to air.

General procedure for the synthesis of P*n*BA and P*t*BA in CYGNET as green solvent *via* SARA ATRP

A series of SARA ATRP reactions were carried out with different target degrees of polymerization (DP_{target}) and catalytic complex concentration (Cu/L, ppm). The mixture of *n*BA (2.00 mL, 13.78 mmol) and *t*BA (2.00 mL, 13.53 mmol), Cu^{II}/Me_6TREN (83 μL or 28 μL or 20.7 μL or 13.8 μL or 2.8 μL of 0.05 M in DMF) for Table 6 entries 1 – 5 and Cu^{II}/Me_6TREN (81 μL of 0.05 M in DMF) for Table 6 entry 6, EBiB (25.0 μL 0.17 mmol), and CYGNET (1.92 mL or 1.97 mL or 1.98 mL or 1.99 mL or 2.00 mL or 1.91 mL) was added to a 10 mL Schlenck flask equipped with a magnetic stirrer bar. Next, Cu^0 wire (l = 4 cm, d = 1 mm) previously activated with HCl and washed with water, acetone or THF was placed in a rubber septum, which was then inserted into a Schlenck flask containing the reaction mixture. The flask was placed in an oil bath heated to 75°C and then the mixture was degassed for 10 – 15 min under argon atmosphere. Cu^0 wire was added to the reaction mixture to initiate the polymerization. Samples were withdrawn periodically to follow monomer conversion using ^1H NMR analysis and to check M_n and M_w/M_n of the polymers by GPC analysis. Before GPC analysis the polymer samples were dissolved in THF (1 mL) + toluene (10 μL) as an external standard in the mobile phase and passed through a neutral alumina column with a 0.22 μm syringe filter in order to remove the catalyst. The polymerization was stopped by opening the flask and exposing the catalyst to air.

General procedure for the synthesis of PHEA in CYGNET as green solvent *via* SARA ATRP

The mixture of HEA (2.00 mL, 17.23 mmol), Cu^{II}/Me_6TREN (34 µL of 0.05 M in DMF), EBiB (32.0 µL 0.22 mmol), and CYGNET (1.96 mL) was added to a 10 mL Schlenk flask equipped with a magnetic stirrer bar. Next, Cu^0 wire (l = 4 cm, d = 1 mm) previously activated with HCl and washed with water, acetone or THF was placed in a rubber septum, which was then inserted into a Schlenk flask containing the reaction mixture. The flask was placed in an oil bath heated to 75°C and then the mixture was degassed for 10 – 15 min under argon atmosphere. Cu^0 wire was added to the reaction mixture to initiate the polymerization. Samples were withdrawn periodically to follow monomer conversion using ^1H NMR analysis and to check M_n and M_w/M_n of the polymers by GPC analysis. Before GPC analysis the polymer samples were dissolved in THF (1 mL) + toluene (10 µL) as an external standard in the mobile phase and passed through a neutral alumina column with a 0.22 µm syringe filter in order to remove the catalyst. The polymerization was stopped by opening the flask and exposing the catalyst to air.

General procedure for the polymerization of methacrylates in CYGNET as green solvent *via* SARA ATRP.

A series of SARA ATRP reactions were carried out of various types of (meth)acrylates. MMA (2.90 mL, 27.48 mmol), or HEMA (2.00 mL, 16.41 mmol), or DMAEMA (2.00 mL, 11.69 mmol) or PGMA (1.00 mL, 7.18 mmol), was mixed with $Cu^{II}Br_2/Me_6TREN$ stock solution (55 µL or 33 µL or 23 µL or 14.4 µL of 0.05 M in DMF), EBPA (50 µL 0.34 mmol or 30 µL 0.21 mmol or 21 µL 0.15 mmol or 13.0 µL 0.09 mmol) and CYGNET (2.96 mL or 1.95 mL or 1.99 mL or 0.99 mL). The substances were added to the 10 mL Schlenk flask, already fitted with a magnetic stirrer bar. Next, Cu^0 wire (l = 4 cm or 2 cm, d = 1 mm) previously activated with HCl and washed with water,

acetone or THF was placed in a rubber septum, which was then inserted into a Schlenk flask containing the reaction mixture**.** The Schlenk flask, along with its contents, was positioned in oil bath heated to 75°C. Subsequently, the mixture underwent a degassing process for a duration of 10-15 minutes under an argon atmosphere. Cu^0 wire was added to the reaction mixture to initiate the polymerization. Samples were withdrawn periodically to follow monomer conversion using ^1H NMR analysis and to check M_n and M_w/M_n of the polymers by GPC analysis. Before GPC analysis the polymer samples were dissolved in THF (1 mL) + toluene (10 µL) (for entries 3, 6) or DMF (1 mL + 10 mM LiCl) + toluene (10 µL) (for entries 4-5) as an external standard mobile phase, and passed through a neutral alumina column with 0.22 µm syringe filter in order to remove the catalyst. The polymerization was stopped by opening the flask and exposing the catalyst to air.

General procedure for the polymerization of *n*BA from Rib-Br_2, Trox-Br_{10}, and β-CD-Br_{15} in CYGNET as green solvent *via* SARA ATRP.

*n*BA (1.50 mL, 10.44 mmol or 2.00 mL 13.89 mmol), initiator: Rib-Br_2 (44.0 mg, 0.07 mmol) or Trox-Br_{10} (38.80 mg, 0.02 mmol) or β-CD-Br_{15} (39.0 mg, 0.01 mmol), $Cu^{II}Br_2$/Me_6TREN stock solution (20.9 µL or 27.8 µL of 0.05 M in DMF), and CYGNET (1.48 mL or 1.98 mL), was added to a 10 mL Schlenck flask equipped with a magnetic stirrer bar. Next, Cu^0 wire (l = 3 cm or 4 cm, d = 1 mm) previously activated with HCl and washed with water, acetone or THF was placed in a rubber septum, which was then inserted into a Schlenk flask containing the reaction mixture. The flask was placed in an oil bath heated to 75 °C and then the mixture was degassed for 10 – 15 min under argon atmosphere. Cu^0 wire was added to the reaction mixture to initiate the polymerization. Samples were withdrawn periodically to follow monomer conversion using ^1H NMR analysis and to check M_n and M_w/M_n of the polymers by GPC analysis. Before GPC analysis the polymer samples were dissolved in THF (1 mL) + toluene (10 µL) as an external standard in the mobile

phase and passed through a neutral alumina column with a 0.22 μm syringe filter in order to remove the catalyst. The polymerization was stopped by opening the flask and exposing the catalyst to air.

Purification of the polymers for 1H NMR analysis

The final polymer products were purified by precipitation in an appropriate solvent or dialysis depending on the polymer type, namely, P*n*BA and PMMA were precipitated in a minimum of 5 folds of a mixture of methanol and water (v/v: 90/10), P*t*BA and P*n*BA-P*t*BA copolymer was dialyzed against methanol, PDMAEMA was obtained by precipitation into excess of hexane, while PGMA was precipitated from cold methanol, PHEA, POEGA and PHEA were dialyzed against water. Then the polymers after precipitation were decanted, and dried under vacuum for 24 h, while the polymer product after dialysis was additionally purified from the solvent by evaporation.

Determination of copper concentration if final polymer sample by atomic absorption spectrometry (AAS).

Mineralization. The strictly measured amounts of polymers (within the range of 100-160 mg) were mineralized in teflon vessels using microwave digestion system. To each sample 6 mL of fuming HNO_3 and 2 mL of 30% H_2O_2 were added. The mineralization process was conducted in 200 °C within 10 minutes. The resulting solutions were diluted to 20 mL using volumetric flasks.

AAS calibration curve. The AAS calibration curve was achieved with the use of $Cu^{II}Br2$ aqueous solutions acidified with HNO_3. The concentrations of the standards were precisely prepared within the range of 0.5-1.5 mg Cu/L. Every point on the curve was the average of three individual measurements completed for each sample.

AAS measurements. Each of the samples obtained after mineralization of the polymers was used directly for AAS analysis. Every result obtained was the average of five individual measurements performed for each sample. In the case of extensively high concentration of same samples necessary dilution was carried out (that the measured value was within the reference curve range) which was taken into account in the final result.

S2. Polymerization of acrylates in *N,N*-dimethylformamide

We conducted a series of model polymerizations using acrylates, specifically nBA and tBA, to compare the obtained results with polymerizations carried out in their bio-based alternatives, Cyrene™ and Cygnet 0.0. The polymerizations of *n*BA were performed at initial molar ratios of $[n\mathrm{BA}]_0/[\mathrm{EBiB}]_0/[\mathrm{Cu^{II}Br_2/TPMA}]_0$ = 80-120/1/0.036, varying in the targeted degree of polymerization. Linear pseudo-first-order kinetic plots, covering up to 80% of monomer conversion, indicated a consistent radical flux within the system (**Figure S1a**). Additionally, the number-average molecular weight of the polymers exhibited a linear increase with monomer conversion (**Figure S1b**), suggesting controlled polymerization. The final polymer displayed low dispersity (M_w/M_n = 1.10-1.11) (**Figure S3a, b**), and the theoretical molecular weight closely matched the apparent one, resulting in an initiation efficiency slightly exceeding 100%. The polymerization of *t*BA, conducted at a $[t\mathrm{BA}]_0/[\mathrm{EBiB}]_0/[\mathrm{Cu^{II}Br_2/TPMA}]_0$ = 80-120/1/0.036 molar ratio, exhibited similar kinetic behavior (**Figure S2**). The reaction nearly halted at around 80% of *t*BA conversion, ultimately yielding a low dispersity final polymer product (**Figure S3c**).

Table S1. Polymerization of *n*BA and *t*BA in *N,N*-dimethylformamide *via* SARA ATRP. [a]

Entry	Monomer	$[\mathrm{Cu/L}]_0$ [ppm]	$\mathrm{DP_{target}}$	t [h]	Conv[b] [%]	k_p^{app}[b]	$M_{n,the}$[c] [x10^{-3}]	$M_{n,app}$[d] [x10^{-3}]	M_w/M_n[d]	I_{eff}[e] [%]
1	*n*BA	300	120	2.00	80	1.041	12,3	11,2	1.11	112
2	*n*BA	300	80	2.50	82	0.941	8,6	7,9	1.10	109
3	*t*BA	300	80	3.00	80	0.713	8,4	7,0	1.14	120

[a]General reaction conditions: T = 50°C; V_{tot} = 4 mL; Argon atmosphere; $[\mathrm{Monomer}]_0$ = 50% v/v; entry 1: $[n\mathrm{BA}]_0$ = 3.46 M, $[n\mathrm{BA}]_0/[\mathrm{EBiB}]_0/[\mathrm{Cu^{II}Br_2/TPMA}]_0$ = 120/1/0.036; entry 2: $[n\mathrm{BA}]_0$ = 3.46 M, $[n\mathrm{BA}]_0/[\mathrm{EBiB}]_0/[\mathrm{Cu^{II}Br_2/TPMA}]_0$ = 80/1/0.024 and entry 3: $[t\mathrm{BA}]_0$ = 3.38 M, $[t\mathrm{BA}]_0/[\mathrm{EBiB}]_0/[\mathrm{Cu^{II}Br_2/TPMA}]_0$ = 80/1/0.024. SARA ATRP with copper wire: d = 0.1 cm, l = 4 cm;

[b]Monomer conversion, apparent rate constant of propagation (k_p^{app}) and apparent theoretical degree of polymerization of monomer unit ($DP_{n,theo}$) were determined by NMR;

[c]$M_{n,theo}$ = ([Monomer]$_0$/[EBiB]$_0$) × conversion × $M_{Monomer}$ + M_{EBiB};

[d]Apparent M_n and M_w/M_n were determined by GPC;

[e]Initiation efficiency, $I_{eff} = (M_{n,theo}/M_{n,app}) \cdot 100\%$.

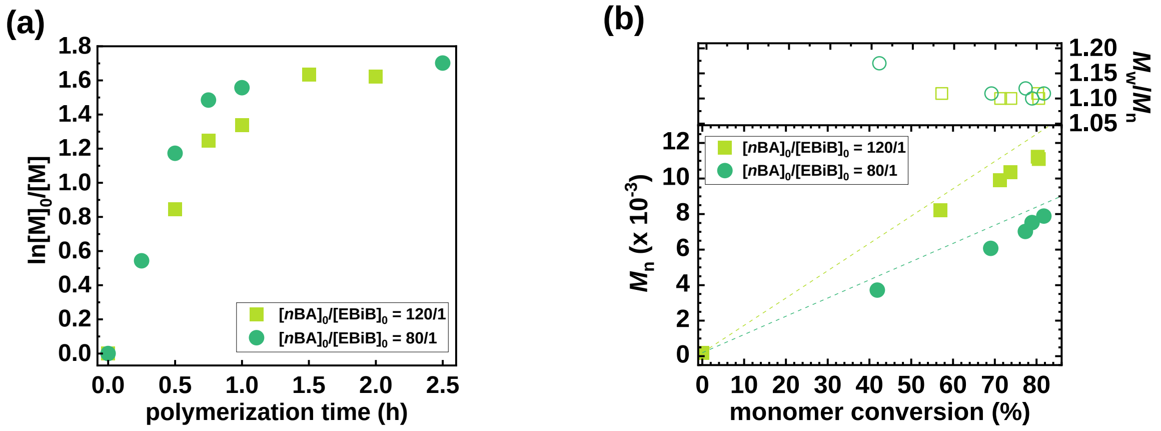

Figure S1. Influence of targeted degree of polymerization on SARA ATRP of *n*BA in DMF. (a) First-order kinetics plots of monomer conversion vs. polymerization time and (b) M_n and M_w/M_n vs. monomer conversion (**Table S1**, entries 1 and 2).

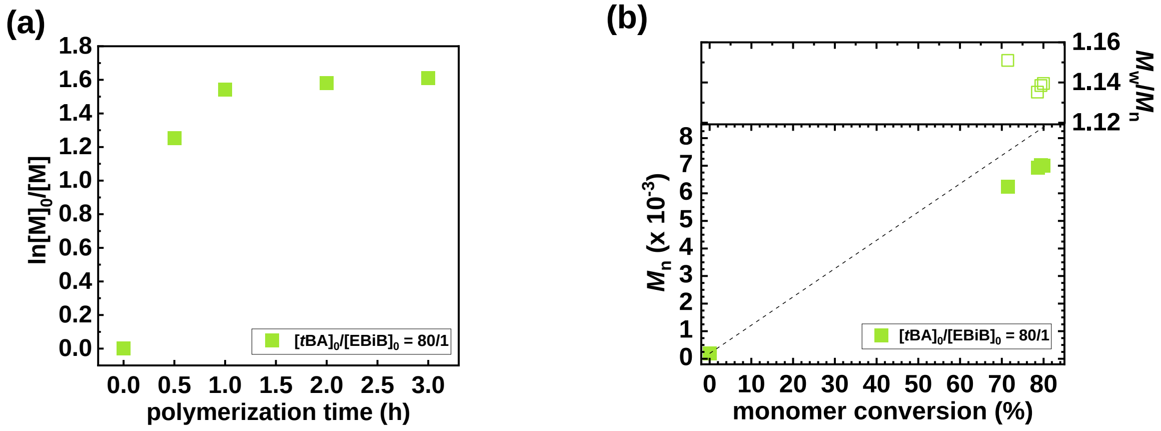

Figure S2. Polymerization of *t*BA by SARA ATRP in DMF. (a) First-order kinetics plots of monomer conversion vs. polymerization time and (b) M_n and M_w/M_n vs. monomer conversion (**Table S1**, entry 3).

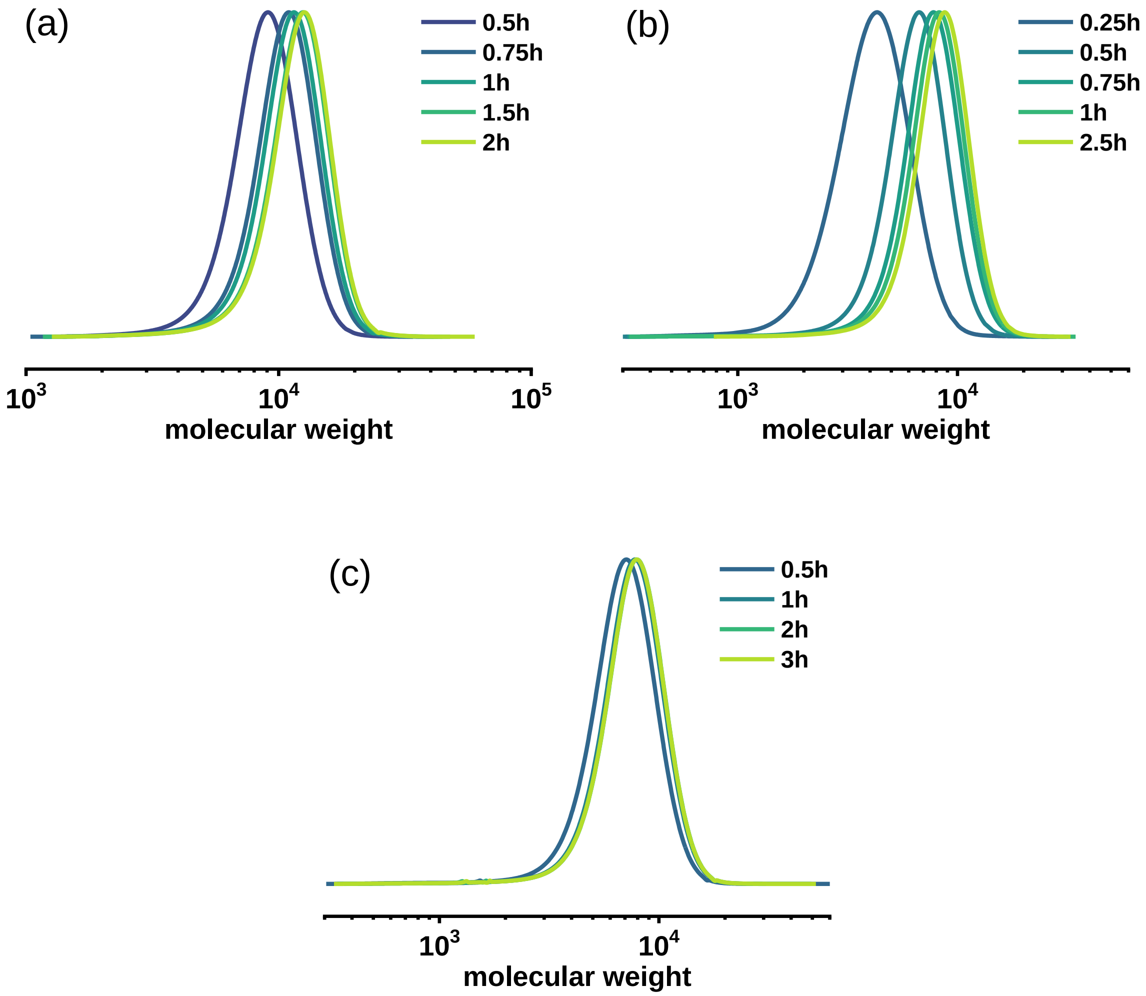

Figure S3. GPC traces of the polymers synthesized in *N,N*-dimethylformamide *via* SARA ATRP of (a) *n*BA, DP_{target} = 120 (b) *n*BA, DP_{target} = 80 and (c) *t*BA, DP_{target} = 80. Reactions conditions as in **Table S1**.

S3. Polymerization of *n*BA in Cyrene™ using TPMA-containing catalytic complex

Table S2. Polymerization of *n*BA in CYRENE™ *via* various low ppm ATRP techniques catalyzed by TPMA-containing catalytic complex. [a]

Entry	Reducing agent	DP_{theo}	[Cu/L] [ppm]	t [h]	Conv[f] [%]	k_p^{app} [f]	$M_{n,theo}$[g] [x10-³]	$M_{n,app}$[h] [x10-³]	M_w/M_n[h]	I_{eff}[j] [%]
**1**	Cu^0	80	300	97.20	84	0.022	8,8	5,1	1.28	174
**2**[b]	Cu^0	80	300	47.00	93	0.116	9,7	4,9	1.18	197
**3**	Cu^0	40	300	214.95	93	0.013	5,0	3,3	1.28	157
**4**	Cu^0	80	600	122.23	71	0.011	7,5	3,0	1.47	199
**5**[c]	AsAc	40	300	29.08	47	0.028	2,59	0,4	1.30	612
**6**[d]	electric current	80	300	22.50	84	0.087	8,9	4,1	1.35	214
**7**[e]	electric current	80	300	20.75	85	0.094	8,9	4,7	1.23	190

[a]General reaction conditions: T = 50°C; V_{tot} = 4 mL; Argon atmosphere; $[nBA]_0$ = 50% v/v; SARA ATRP with copper wire: d = 0.1 cm, l = 4 cm; S/V = 0.318 cm^{-1} for entries 1, 2 and 4, $[nBA]_0$ = 3.46 M; L = TPMA;

[b]SARA ATRP with copper wire: d = 0.1 cm, l = 8 cm; 4 copper wires 2 cm long were introduced into the reaction mixture at appropriate time intervals: 0h, 0.5h, 1h, 1.5h; S/V = 0.632 cm^{-1};

[c]ARGET ATRP with the use of ascorbic acid as a reducing agent;

[d]Constant potential *se*ATRP (WE = Pt mesh, CE = Al wire, RE = SCE), applied potential (E_{app}) was selected based on CV analysis of catalyst complex (E_{app}=E_{pc}− 40 mV vs SCE;

[e]Constant potential *e*ATRP (WE = Pt mesh, CE = Pt wire, RE = SCE), applied potential (E_{app}) was selected based on CV analysis of catalyst complex (E_{app}=E_{pc}− 40 mV vs SCE ;

[f]Monomer conversion, apparent rate constant of propagation (k_p^{app}) and apparent theoretical degree of polymerization of monomer unit ($DP_{n,theo}$) were determined by NMR;

[g]$M_{n,theo} = ([nBA]_0/[EBiB]_0) \times \text{conversion} \times M_{nBA} + M_{EBiB}$;

^{h}Apparent M_n and M_w/M_n were determined by GPC;

^{i}Initiation efficiency, $I_{eff} = (M_{n,theo}/M_{n,app}) \cdot 100\%$.

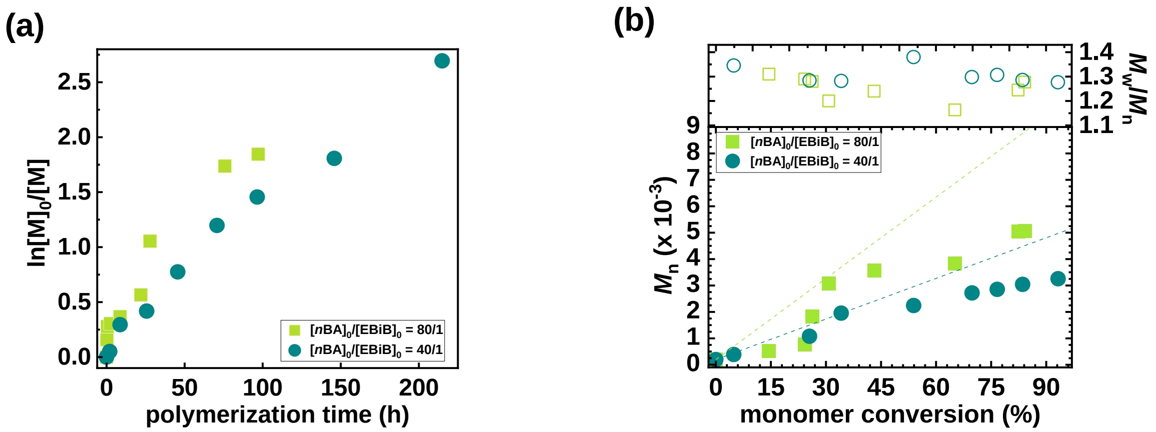

Figure S4. Influence of targeted degree of polymerization on SARA ATRP of *n*BA in CYRENE™. (a) First-order kinetics plots of monomer conversion vs. polymerization time and (b) M_n and M_w/M_n vs. monomer conversion (**Table S2**, entries 1 and 3).

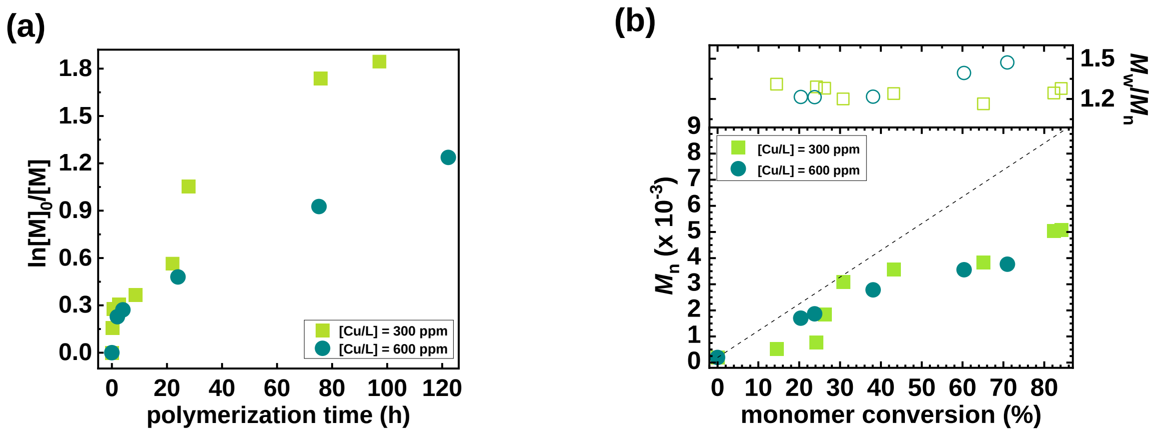

Figure S5. Effect of catalyst concentration on SARA ATRP of *n*BA in CYRENE™. (a) First-order kinetics plots of monomer conversion vs. polymerization time and (b) M_n and M_w/M_n vs. monomer conversion (**Table S2**, entries 1 and 3).

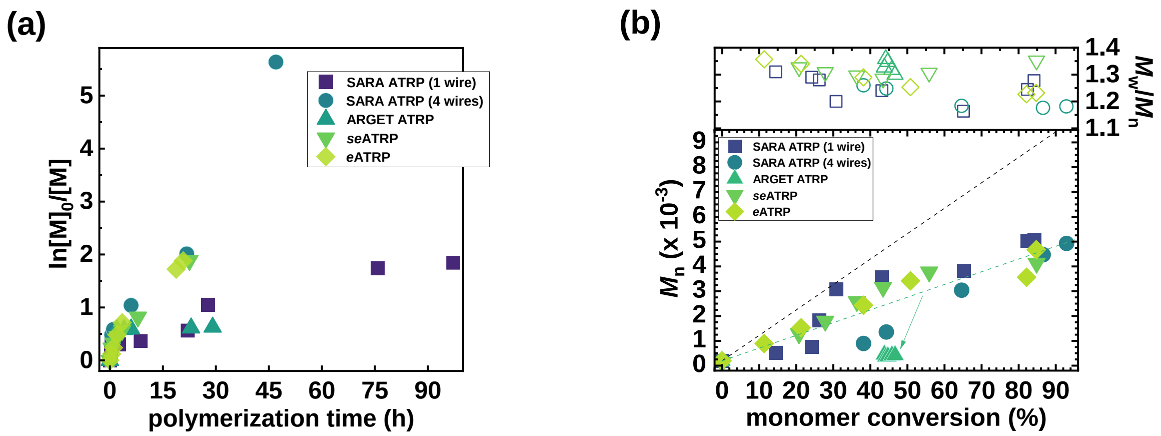

Figure S6. Effect of applied ATRP technique on *n*BA polymerization in CYRENE™ using TPMA as a ligand in catalytic complex. (a) First-order kinetics plots of monomer conversion vs. polymerization time and (b) M_n and M_w/M_n vs. monomer conversion (**Table S2**, entries 1, 4-7).

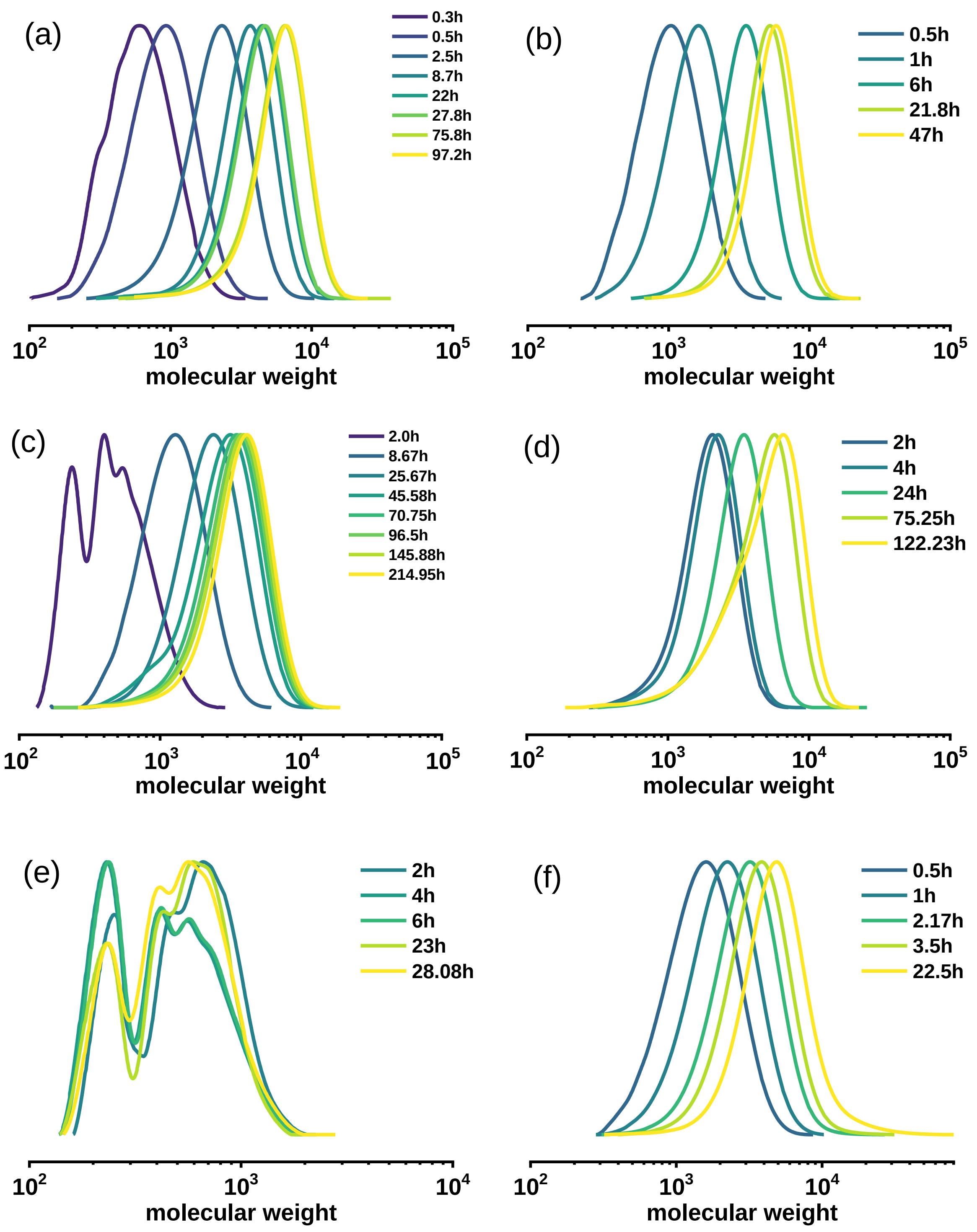
(a)
0.3h
0.5h
2.5h
8.7h
22h
27.8h
75.8h
97.2h
10^2 10^3 10^4 10^5
molecular weight
(b)
0.5h
1h
6h
21.8h
47h
10^2 10^3 10^4 10^5
molecular weight
(c)
2.0h
8.67h
25.67h
45.58h
70.75h
96.5h
145.88h
214.95h
10^2 10^3 10^4 10^5
molecular weight
(d)
2h
4h
24h
75.25h
122.23h
10^2 10^3 10^4 10^5
molecular weight
(e)
2h
4h
6h
23h
28.08h
10^2 10^3 10^4
molecular weight
(f)
0.5h
1h
2.17h
3.5h
22.5h
10^2 10^3 10^4
molecular weight

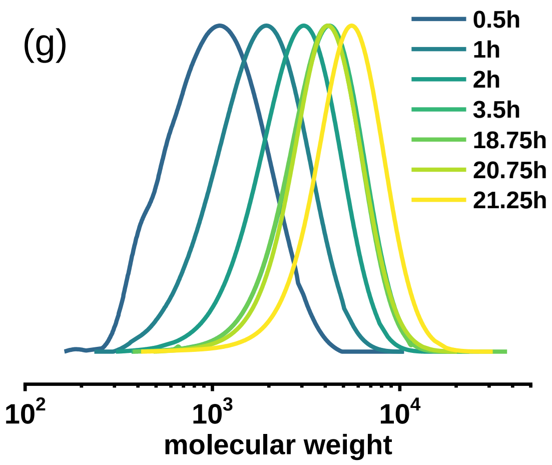

Figure S7. GPC traces of the P*n*BA synthesized in Cyrene™ using TPMA as a ligand in catalytic complex *via* SARA ATRP at conditions (a) DP_{target} = 80, $[Cu/L]_0$ = 300 ppm (**Table S2,** entry 1), (b) DP_{target} = 80, $[Cu/L]_0$ = 300 ppm, four separate 2 cm long copper wires were added to the reaction mixture at appropriate intervals (**Table S2,** entry 2), (c) DP_{target} = 40, $[Cu/L]_0$ = 300 ppm (**Table S2,** entry 3), (d) DP_{target} = 80, $[Cu/L]_0$ = 600 ppm (**Table S2,** entry 4); (e) *via* ARGET ATRP (**Table S2,** entry 5); via (f) constant potential *se*ATRP (**Table S2,** entry 6), and (g) constant potential *e*ATRP (**Table S2,** entry 7).

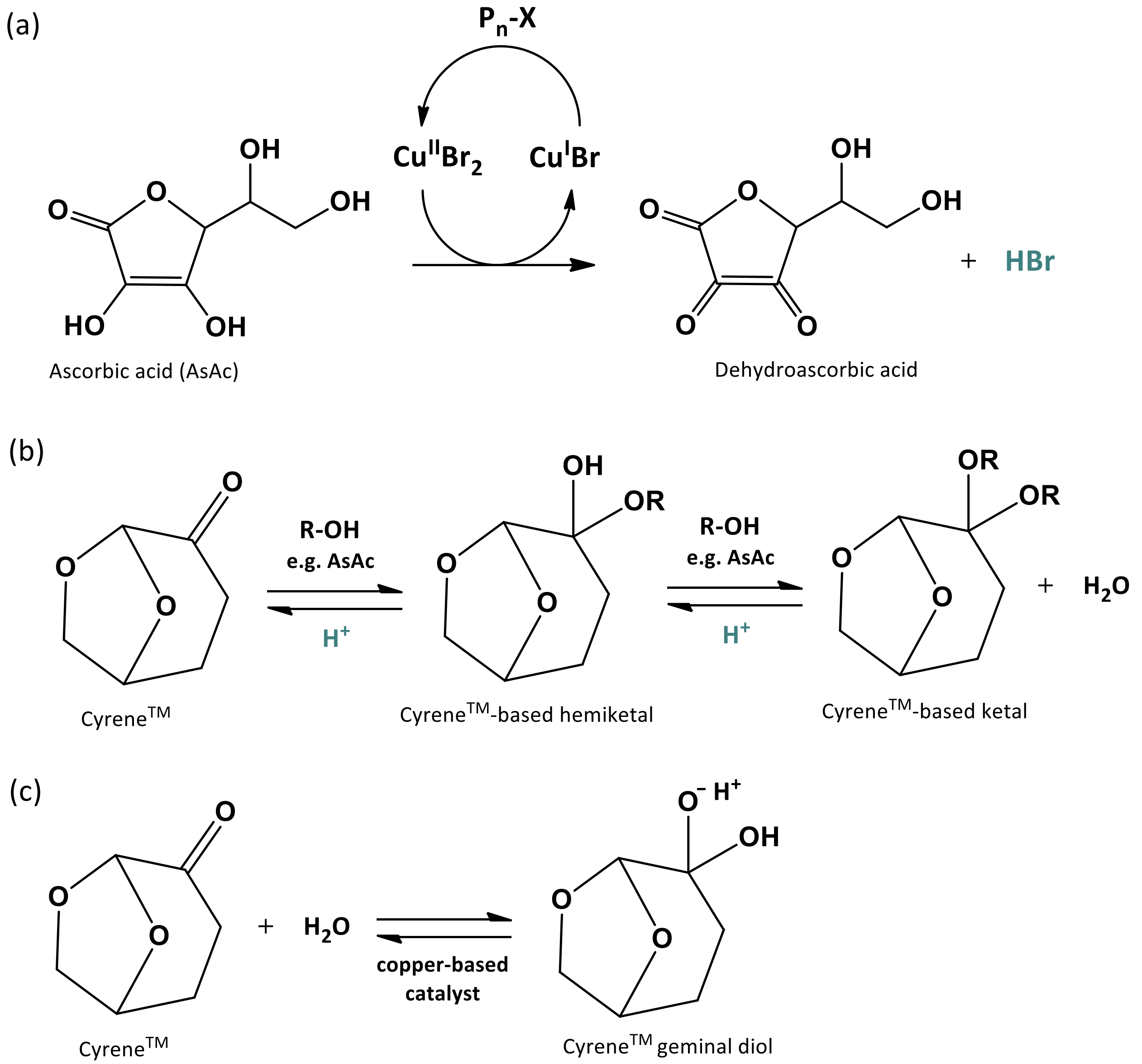

Scheme S1. Example side reactions of Cyeren™ in ATRP reaction mixture (a) formation of strong acid during the reduction of copper-based catalyst by ascorbic acid (reducing agent used in ARGET ATRP), followed by (b) formation of hemiketal/ketal form of Cyrene™ catalyzed by strong acid;[8] (c) formation of Cyrene™ containing geminal diol moiety.[9]

S4. Polymerization of *n*BA by SARA ATRP in Cyrene™ using various catalytic complex

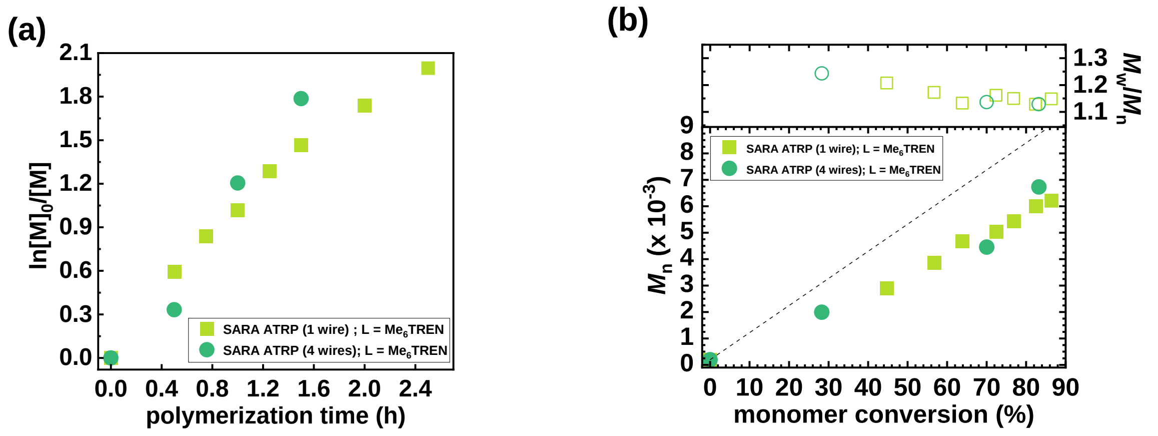

Figure S8. Effect of amount of zerovalent metal on *n*BA polymerization in Cyrene™. (a) First-order kinetics plots of monomer conversion vs. polymerization time and (b) M_n and M_w/M_n vs. monomer conversion (**Table 1**, entries 4 and 5).

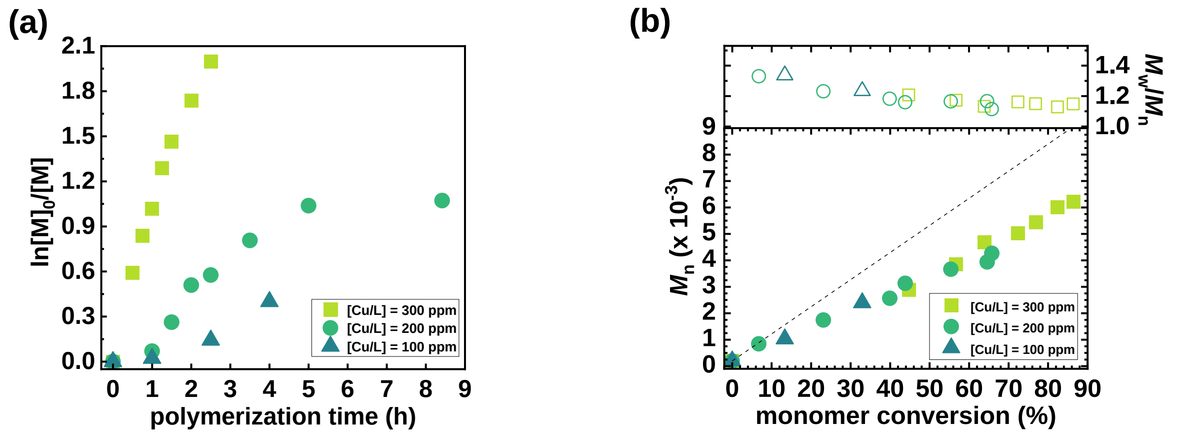

Figure S9. Effect of catalyst concentration on *n*BA polymerization in Cyrene™. (a) First-order kinetics plots of monomer conversion vs. polymerization time and (b) M_n and M_w/M_n vs. monomer conversion (**Table 1**, entries 4, 6 and 7).

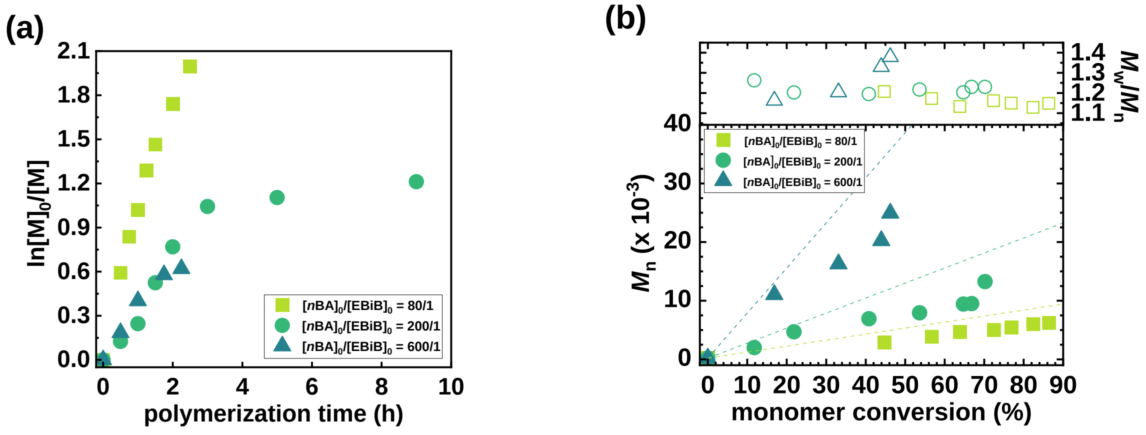

Figure S10. Influence of targeted degree of polymerization on SARA ATRP of *n*BA in Cyrene™. (a) First-order kinetics plots of monomer conversion vs. polymerization time and (b) M_n and M_w/M_n vs. monomer conversion (**Table 1**, entries 4, 8 and 9).

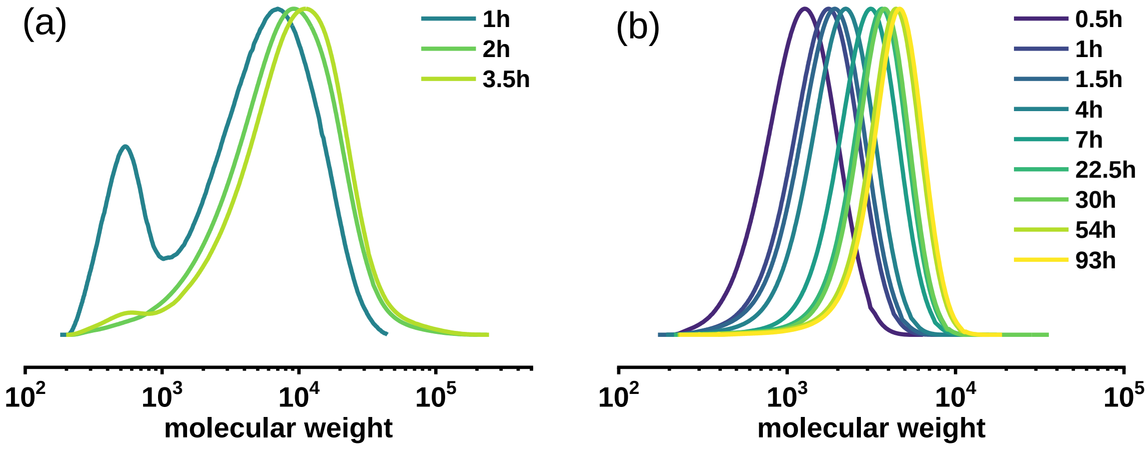

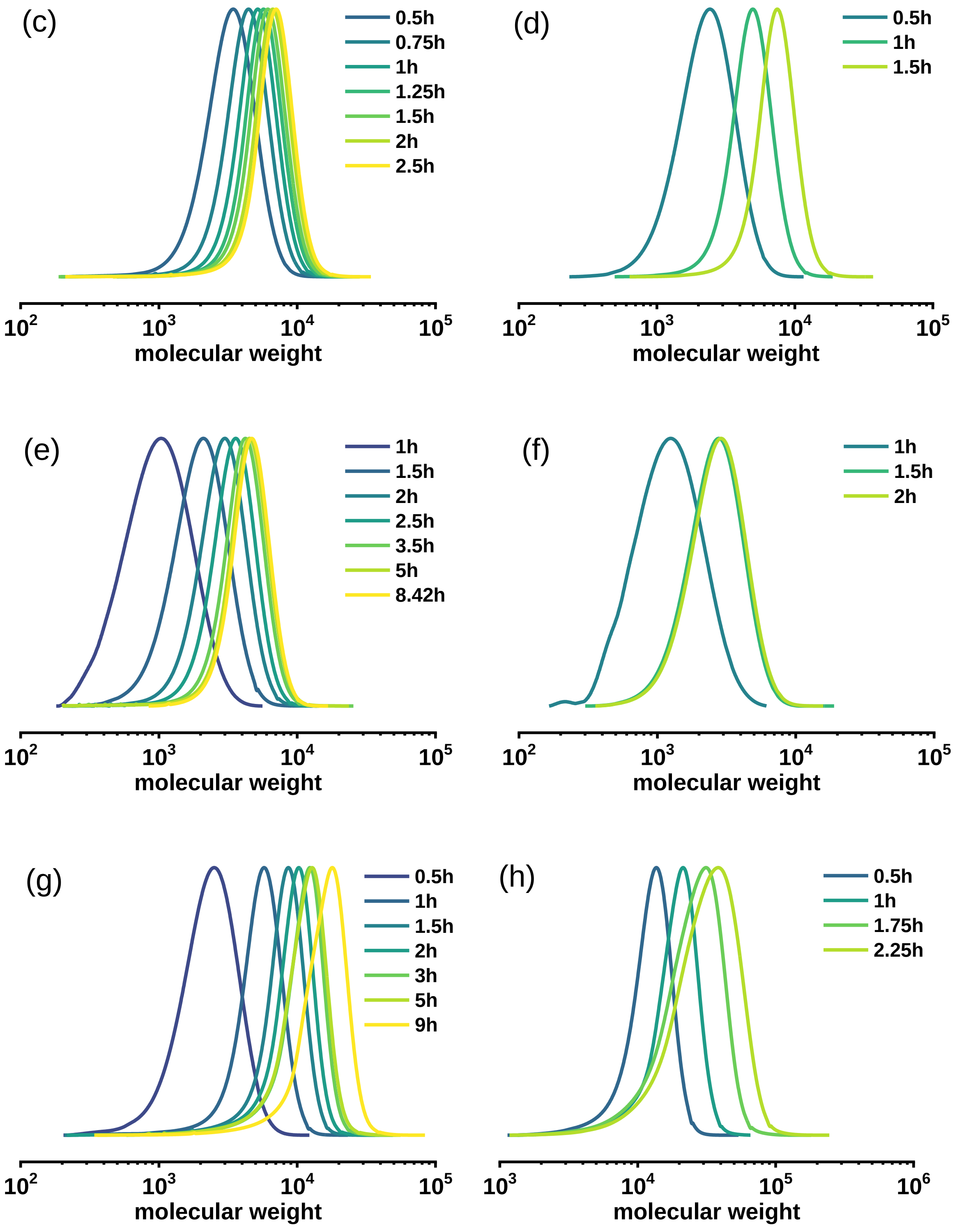

Figure S11. GPC traces of the P*n*BA synthesized in Cyrene™ *via* SARA ATRP using catalytic complex containing (a) PMDETA (**Table 1,** entry 1), (b) TPMA*2 (**Table 1,** entry 3), and

Me_6TREN at various conditions: (c) DP_{target} = 80, $[Cu/L]_0$ = 300 ppm (**Table 1,** entry 4), (d) DP_{target} = 80, $[Cu/L]_0$ = 300 ppm, Four separate 2 cm long copper wires were added to the reaction mixture at appropriate intervals (**Table 1,** entry 5), (e) DP_{target} = 80, $[Cu/L]_0$ = 200 ppm (**Table 1,** entry 6), (f) DP_{target} = 80, $[Cu/L]_0$ = 100 ppm (**Table 1,** entry 7), (g) DP_{target} = 200, $[Cu/L]_0$ = 300 ppm (**Table 1,** entry 8), (h) DP_{target} = 600, $[Cu/L]_0$ = 300 ppm (**Table 1,** entry 9).

S5. Electrochemical characterization of copper-based catalytic complex in Cyrene™ and Cygnet compared to *N,N*-dimethylformamide

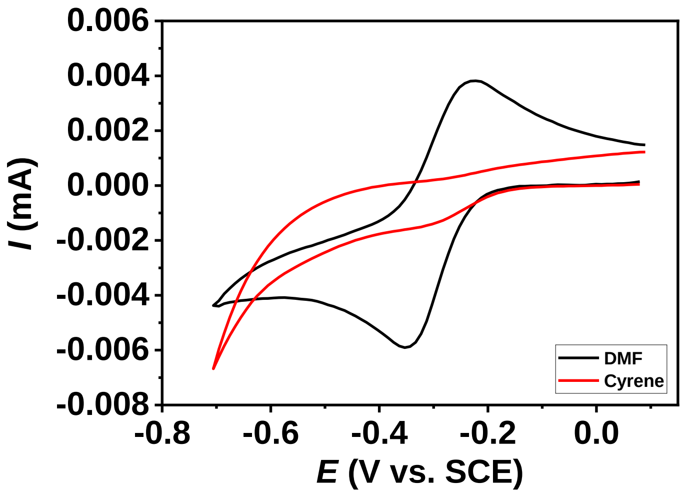

Figure S12. Cyclic voltammogram of 0.69 mM $Cu^{II}Br_2$/TPMA in DMF (black line) and in Cyrene™ (red line), containing 0.2 M TBAP and 3.44 M *n*BA, recorded at v = $0.1 V \cdot s^{-1}$, at room temperature.

Cyclic voltammetry of $Cu^{II}Br_2/Me_6TREN$ in Cygnet 0.0 was carried out using a mixture of Cygnet 0.0 and nBA (50/50% v/v) at 75°C (**Figure S13**). This solvent is not soluble in nBA, and at room temperature, it exists as a solid with a melting point of 71°C.[10] However, when the mixture of nBA and Cygnet 0.0 was heated above 71°C, the solvent became miscible with the monomer. To compare the behavior of the catalytic complex in Cyrene™ and Cygnet 0.0, CV measurements of the catalytic complex were also conducted in Cyrene™ at 75°C. It's important to note that an increase in temperature led to a rise in the cathodic peak current. This change can be attributed to the shift in the diffusion coefficient, affecting mass transport, and, in general, causing an increase in peak current when the temperature is elevated.[11] Elevated temperature also caused the cathodic current for the reduction of the catalytic complex to shift towards more positive potentials. This

shift suggests the presence of a dissociated deactivator form of the catalyst in the system being studied. Specifically, the Br-Cu^{II}/Me6TREN complex dissociates into Br- + Cu^{II}/Me6TREN.[12] The further discussion on the comparison the voltammetric behavior of the catalyst in various solvents is included in the *Results and discussion* part of the manuscript.

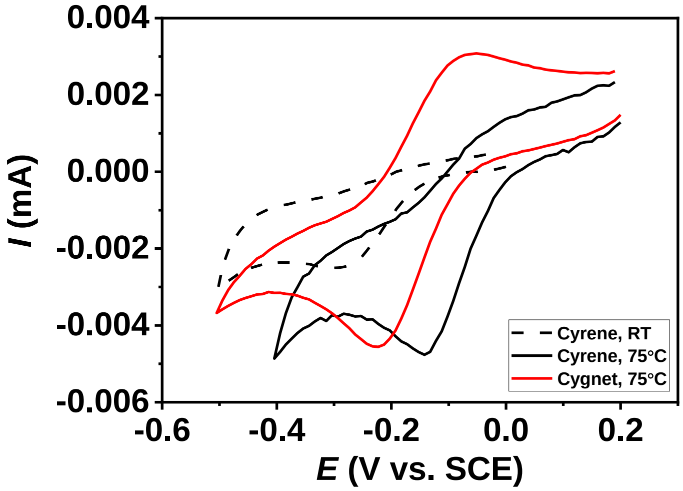

Figure S13. Cyclic voltammogram of 0.69 mM $Cu^{II}Br_2/Me_6TREN$ in Cygnet at room temperature (dashed black line) and 75°C (solid black line), and in Cygnet 0.0 at and 75°C (red line), containing 0.2 M TBAP and 3.44 M *n*BA, recorded at v = 0.1V·s^{-1}.

S6. Polymerization of various monomers in Cyrene™ by SARA ATRP

Table S3. Polymerization of different acrylates and methacrylates *via* SARA ATRP in CYRENE™ as a green solvent. [a]

Entry	Monomer	Initiator	t [h]	Conv[b] [%]	$k_p^{app b}$	$M_{n,theo}$[c] [x10-3]	$M_{n,app}$[d] [x10-3]	M_w/M_n[d]	I_{eff}[f] [%]
**1**[g]	*n*BA	EBiB	2.50	86	0.896	9,1	6,2	1.15	146
**2**	*t*BA	EBiB	4.80	94	0.598	9,8	7,4	1.15	133
**3**	HEA	EBiB	2.08	22	0.150	2,3	12,2	1.36	19
**4**	$OEGA_{480}$	EBiB	215.75	72	0.005	27,7	11,4	1.23	244
**5**	MMA	EBPA	48.00	79	0.034	6,6	1,7	1.33	382
**6**	HEMA	EBPA	50.50	75	0.025	8,0	9,9	1.47	81
**7**	GMA	EBPA	0.90	89	2.360	10,4	16,9	1.43	61.4
**8**	DMAEMA	EBPA	2.50	83	0.822	10,7	17,8	1.50	60.0

[a]General reaction conditions: T = 50°C; V_{tot} = 4 mL; Argon atmosphere; $[\text{Monomer}]_0$ = 50% v/v; $[\text{Monomer}]_0/[\text{initiator}]_0/[Cu^{II}Br_2/Me_6TREN]_0$ = 80/1/0.024; SARA ATRP with copper wire: d = 0.1 cm, l = 4 cm;

[b]Monomer conversion, apparent rate constant of propagation (k_p^{app}) and apparent theoretical degree of polymerization of monomer unit ($DP_{n,theo}$) were determined by NMR;

[c]$M_{n,theo} = ([\text{Monomer}]_0/[\text{initiator}]_0) \times \text{conversion} \times M_{\text{Monomer}} + M_{\text{initiator}}$;

[d]Apparent M_n and M_w/M_n were determined by GPC;

[e]$DP_{n,app} = M_{n,app}/M_{\text{Monomer}}$;

[f]Initiation efficiency, $I_{eff} = (M_{n,theo}/M_{n,app}) \cdot 100\%$;

[g]Reaction results presented also in **Table 1**, entry 4.

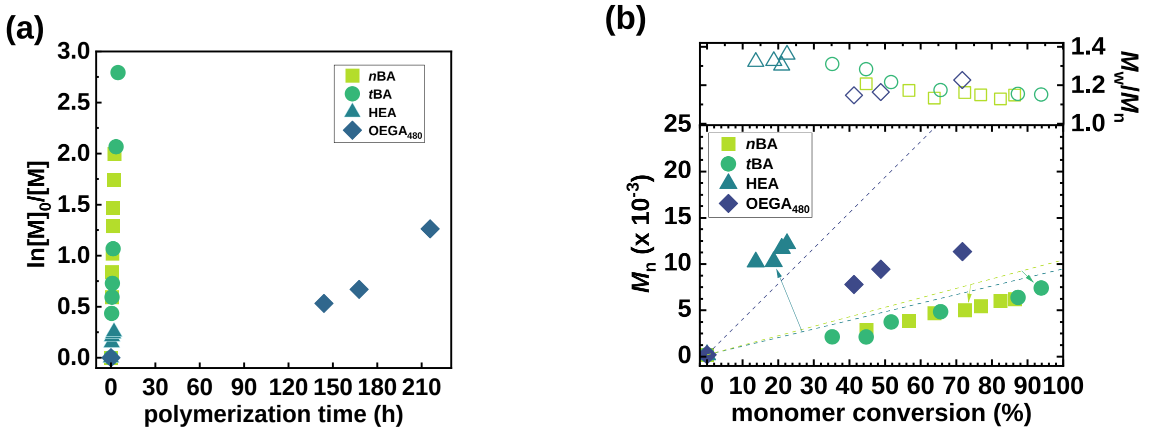

Figure S14. Polymerization of various acrylates in CYRENE™. (a) First-order kinetics plots of monomer conversion vs. polymerization time and (b) M_n and M_w/M_n vs. monomer conversion (**Table S3**, entries 1-4).

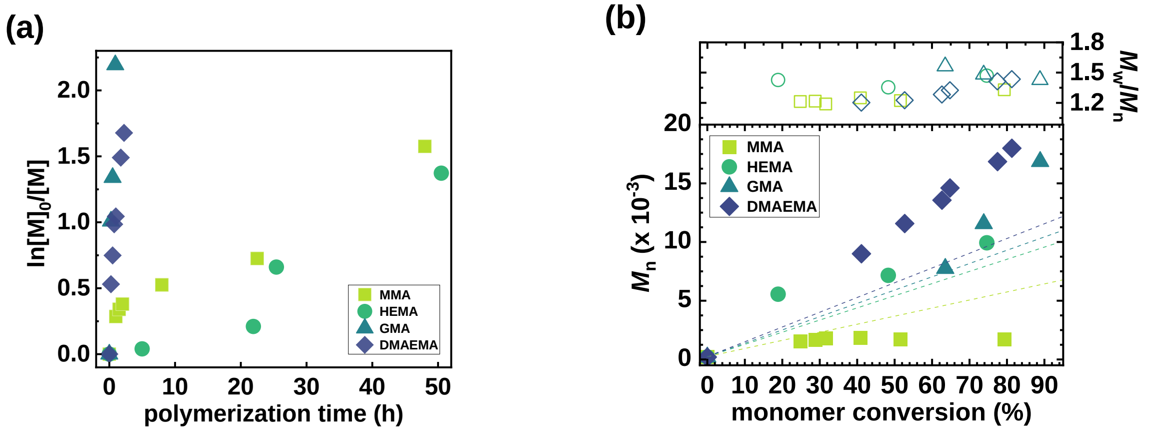

Figure S15. Polymerization of various methacrylates in CYRENE™. (a) First-order kinetics plots of monomer conversion vs. polymerization time and (b) M_n and M_w/M_n vs. monomer conversion (**Table S3**, entries 5-8).

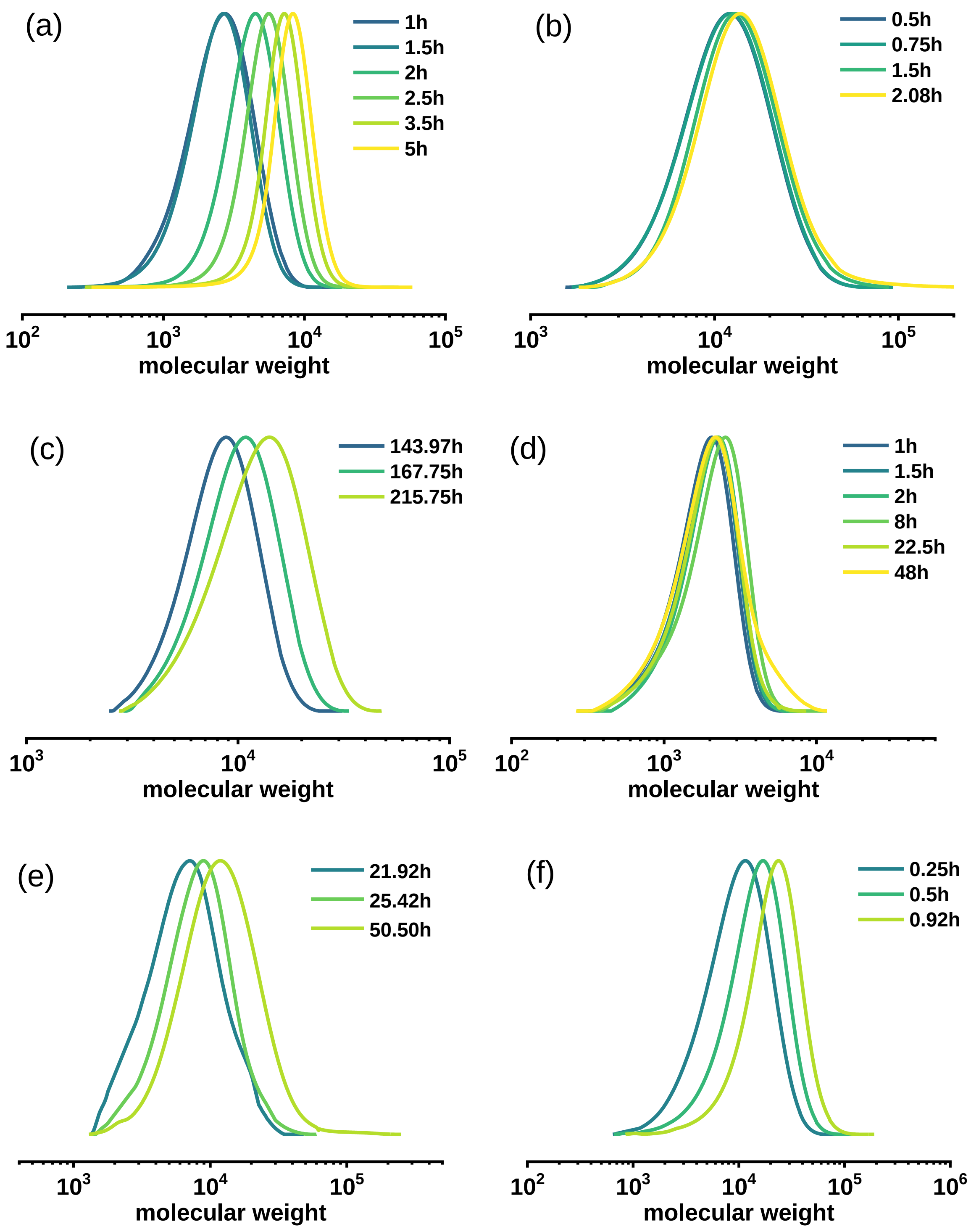
(a)
1h
1.5h
2h
2.5h
3.5h
5h
10^2 10^3 10^4 10^5
molecular weight
(b)
0.5h
0.75h
1.5h
2.08h
10^3 10^4 10^5
molecular weight
(c)
143.97h
167.75h
215.75h
10^3 10^4 10^5
molecular weight
(d)
1h
1.5h
2h
8h
22.5h
48h
10^2 10^3 10^4
molecular weight
(e)
21.92h
25.42h
50.50h
10^3 10^4 10^5
molecular weight
(f)
0.25h
0.5h
0.92h
10^2 10^3 10^4 10^5 10^6
molecular weight

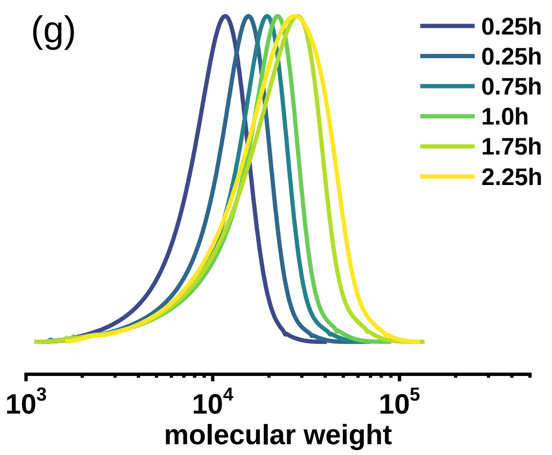

Figure S16. GPC traces of (a) P*t*BA (**Table S3**, entry 2), (b) PHEA (**Table S3**, entry 3), (c) POEGA (**Table S3**, entry 4), (d) PMMA (**Table S3**, entry 5), (e) PHEAM (**Table S3**, entry 6), (f) PGMA (**Table S3**, entry 7) and (g) PDMAEMA (**Table S3**, entry 8) synthesized in Cyrene™ *via* SARA ATRP.

S7. Chain extension of P*n*BA by SARA ATRP in Cyrene™

Table S4. Chain extension of P*n*BA by P*t*BA as a second block in CYRENE™. [a]

Entry	$[Cu/L]_0$ [ppm]	t [h]	Conv[b] [%]	k_p^{app}[b]	$M_{n,theo}$[c] [x10-3]	$M_{n,app}$[d] [x10-3]	M_w/M_n[d]	I_{eff}[e] [%]
**2**	300	23	37	0.021	20,3	8,9	1.11	230
**3**	700	25	46	0.034	15,8	11,9	1.61	133

[a]General reaction conditions: SARA ATRP of *t*BA; $[tBA]_0 = 1.51$ M; $[Cu^{II}Br_2/Me_6TREN]_0 = 0.09$ M for entry 2 and $Cu^{II}Br_2/Me_6TREN]_0 = 0.21$ M for entry 3; T = 50°C; $V_{tot} = 4$ mL; Argon atmosphere; *N*,*N*-dimethylformamide as a solvent; SARA ATRP with copper wire: d = 0.1 cm, l = 4 cm, the final polymer product from reaction presented in **Table 1**, entry 4 was used as a macroinitiator (P*n*BA, $M_n = 6{,}213$ g/mol, $M_w/M_n = 1.15$);

[b]Monomer conversion, apparent rate constant of propagation (k_p^{app}) and apparent theoretical degree of polymerization of monomer unit ($DP_{n,theo}$) were determined by NMR;

[c]$M_{n,theo} = ([Monomer]_0/[EBiB]_0) \times \text{conversion} \times M_{Monomer} + M_{EBiB}$;

[d]Apparent M_n and M_w/M_n were determined by GPC;

[e]Initiation efficiency, $I_{eff} = (M_{n,theo}/M_{n,app}) \cdot 100\%$.

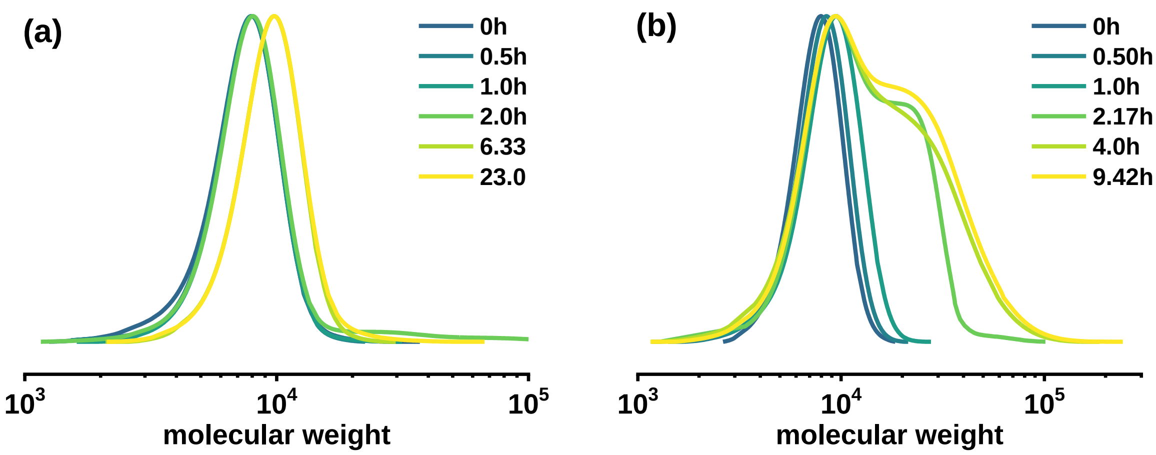

Figure S17. GPC traces of P*n*BA-*b*-P*t*BA synthesized using (a) 300 ppm and (b) 700 ppm of catalyst (**Table S4**).

S8. Polymerization of various acrylates and methacrylates *via* SARA ATRP in Cygnet 0.0

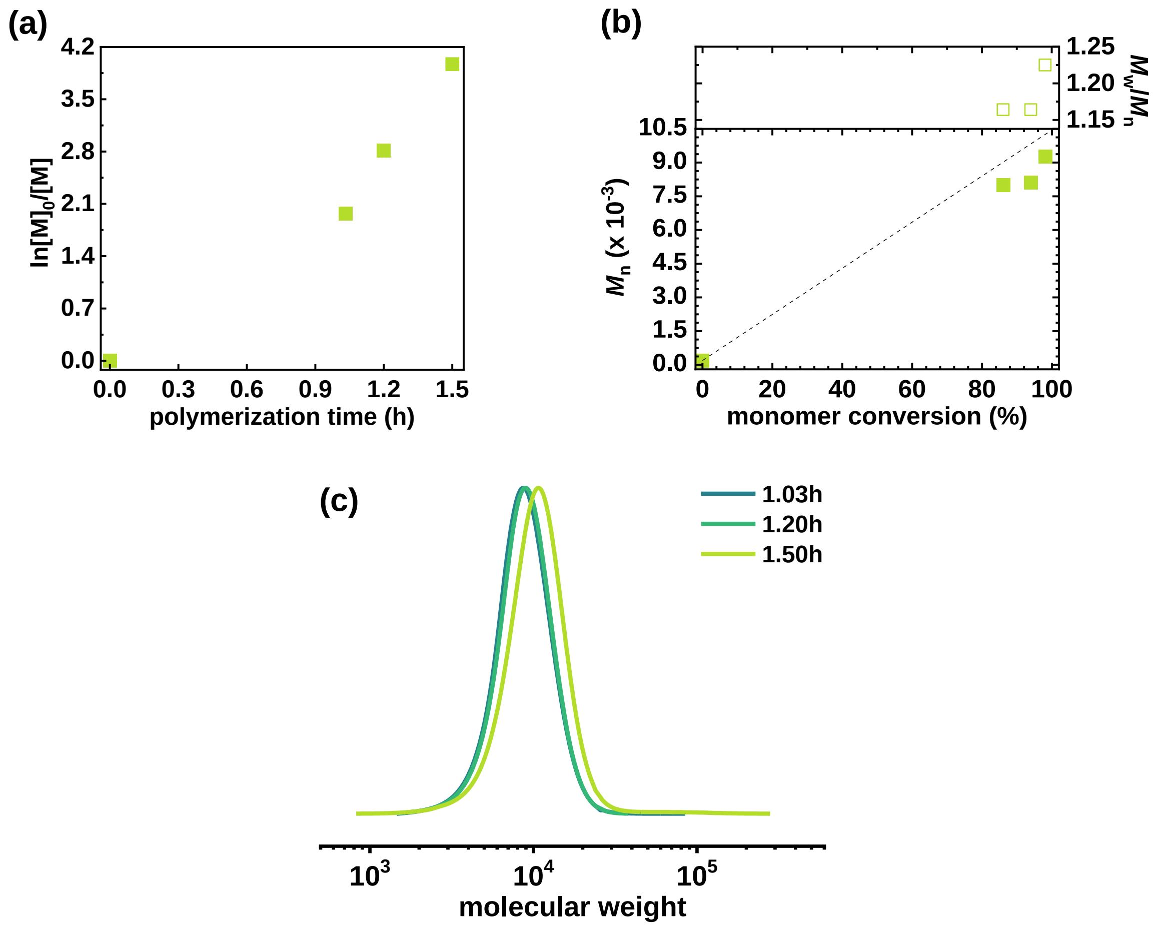

Figure S18. Polymerization of *t*BA via SARA ATRP in Cygnet 0.0. (a) First-order kinetics plots of monomer conversion vs. polymerization time, (b) M_n and M_w/M_n vs. monomer conversion, and (c) GPC traces of P*t*BA (**Table 3**, entry 2).

Table S5. Polymerization of different acrylates and methacrylates *via* SARA ATRP in Cygnet 0.0 as a green solvent. [a]

Entry	Monomer	Initiator	t [h]	Conv[b] [%]	k_p^{app} [b]	$M_{n,the}$ [c] [x10^{-3}]	$M_{n,app}$ [d] [x10^{-3}]	M_w/M_n [d]	I_{eff} [e] [%]
**1**[f]	*n*BA	EBiB	2.50	91	0,6213	9,55	8,01	1.14	119
**2**	HEA	EBiB	0.23	92	10,865	9,63	22,4	1.36	43
**3**	MMA	EBPA	25	26	0,0151	2,28	0,98	1.38	234
**4**	HEMA	EBPA	25.42	17	0.0084	2,0	15,3	1.87	13

[a]General reaction conditions: T = 75°C; V_{tot} = 4 mL for entries 1 – 2 and 4, and V_{tot} = 5 mL for entry 3; Argon atmosphere; $[Monomer]_0$ = 50% v/v; $[Monomer]_0/[Initiator]_0/[Cu^{II}Br_2/Me_6TREN]_0$ = 80/1/0.008; SARA ATRP with copper wire: d = 0.1 cm, l = 4 cm for entries 1-4 and d = 0.1 cm;

[b]Monomer conversion, apparent rate constant of propagation (k_p^{app}) and apparent theoretical degree of polymerization of monomer unit ($DP_{n,theo}$) were determined by NMR;

[c]$M_{n,theo}$ = ($[Monomer]_0/[initiator]_0$) × conversion × $M_{Monomer}$ + $M_{initiator}$;

[d]apparent M_n and M_w/M_n were determined by GPC;

[e]Initiation efficiency, $I_{eff} = (M_{n,theo}/M_{n,app}) \cdot 100\%$;

[g]Reaction results presented also in **Table 3**, entry 3.

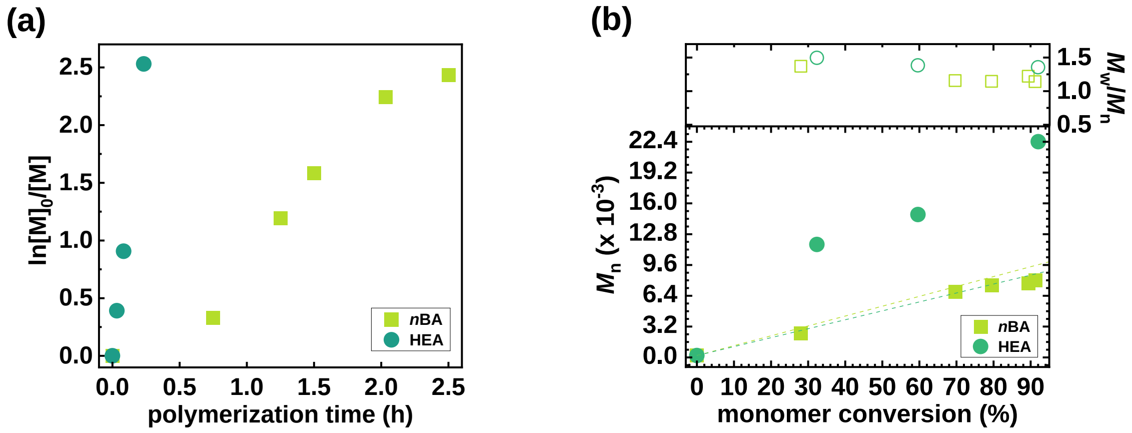

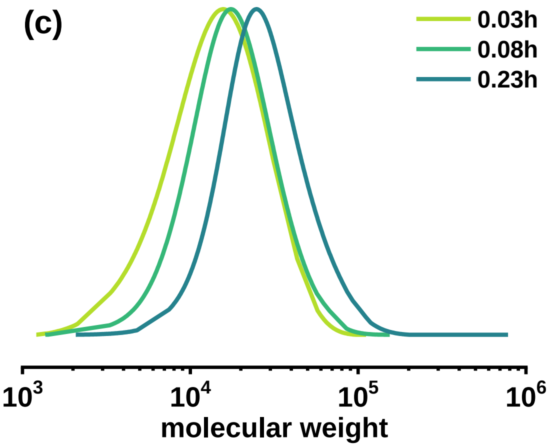

Figure S19. Polymerization of various acrylates in Cygnet 0.0. (a) First-order kinetics plots of monomer conversion vs. polymerization time, (b) M_n and M_w/M_n vs. monomer conversion, and (c) GPC traces of PHEA (**Table S5**, entries 1 and 2).

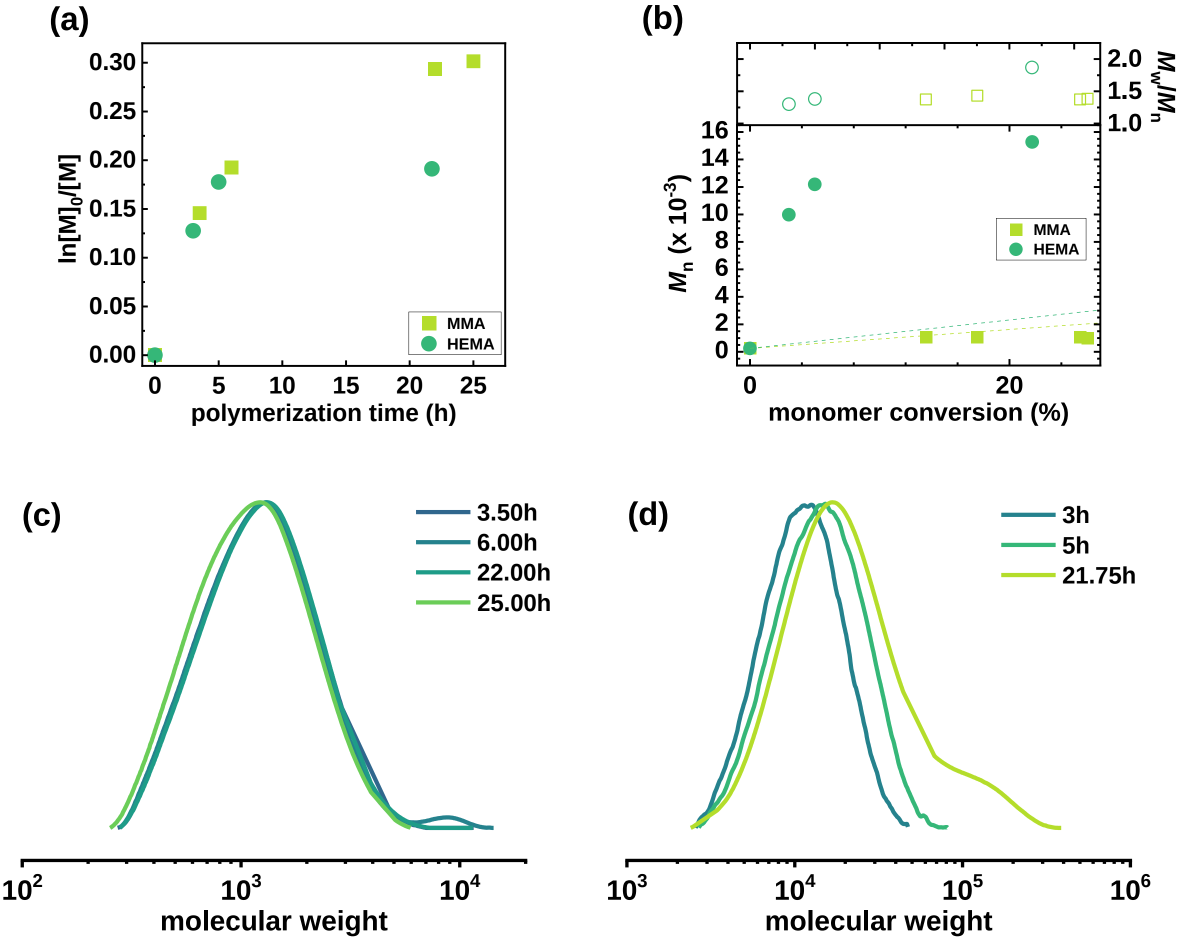

Figure S20. Polymerization of various methacrylates in Cygnet 0.0. (a) First-order kinetics plots of monomer conversion vs. polymerization time, (b) M_n and M_w/M_n vs. monomer conversion, and GPC traces of (c) PMMA and (d) PHEMA (**Table S5**, entries 3 and 4).

S9. Synthetic routes for the synthesis of polymers with centrally-located naturally derived substances in Cyrene™ and Cygnet 0.0 and their structures

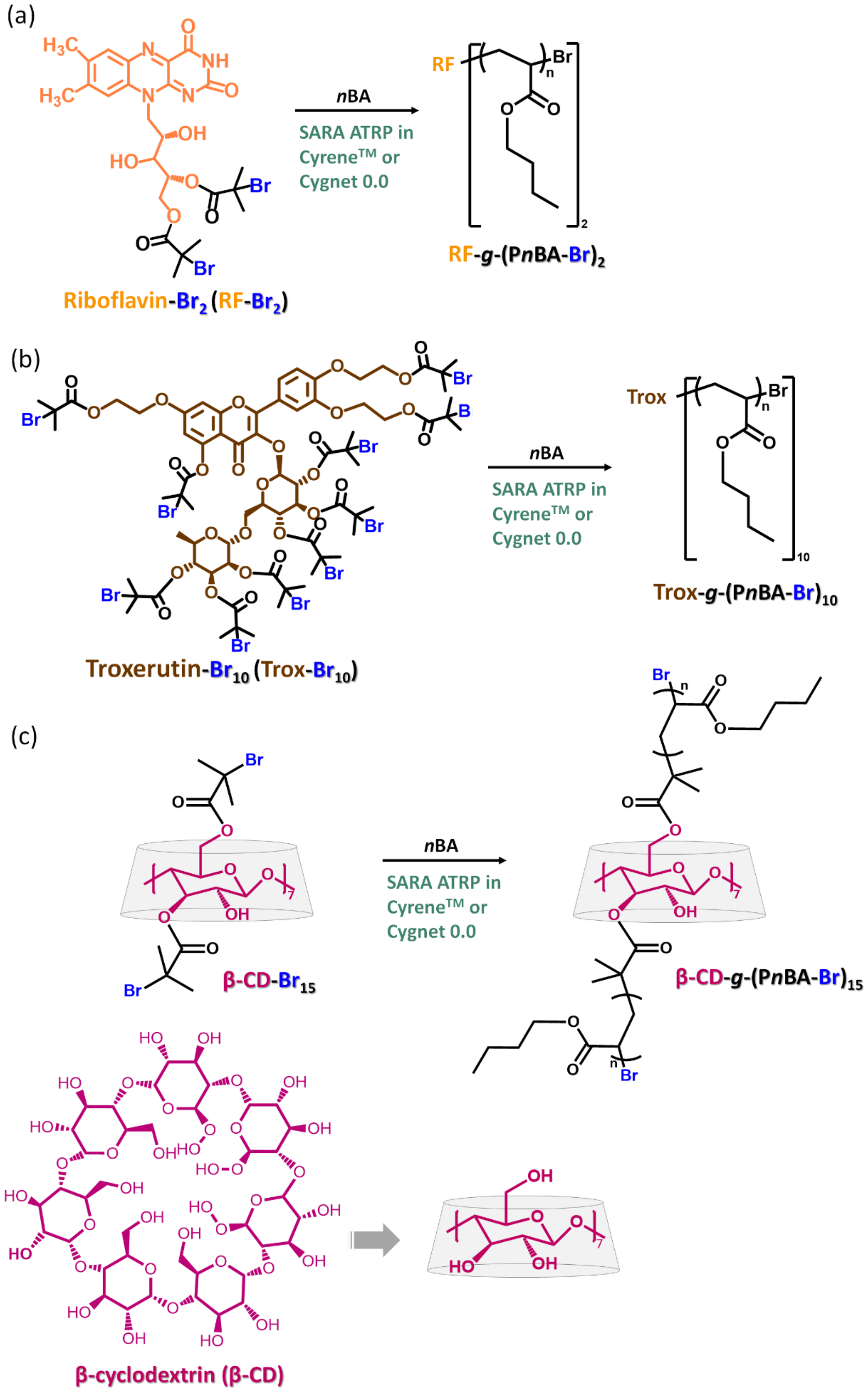

Scheme S2. Synthetic routes for the synthesis of polymers from (a) riboflavin, (b) troxerutin and (c) β-cyclodextrin core by SARA ATRP in Cyrene™ and Cygnet 0.0.

S10. Spectroscopic characterization of the prepared polymers

The structure of P*n*BA formed in DMF was confirmed by ^{1}H NMR analysis (**Figure S21**). The chemical shifts characteristic for **P*n*BA** were assigned: δ (ppm) = 0.78–1.00 (3H, CH_3–, d), 1.31–2.03 (6H, CH_2–, β+b+c), 2.19–2.45 (1H, –C*H*–, α), 3.85–4.23 (2H, CH_2–, a).[4]

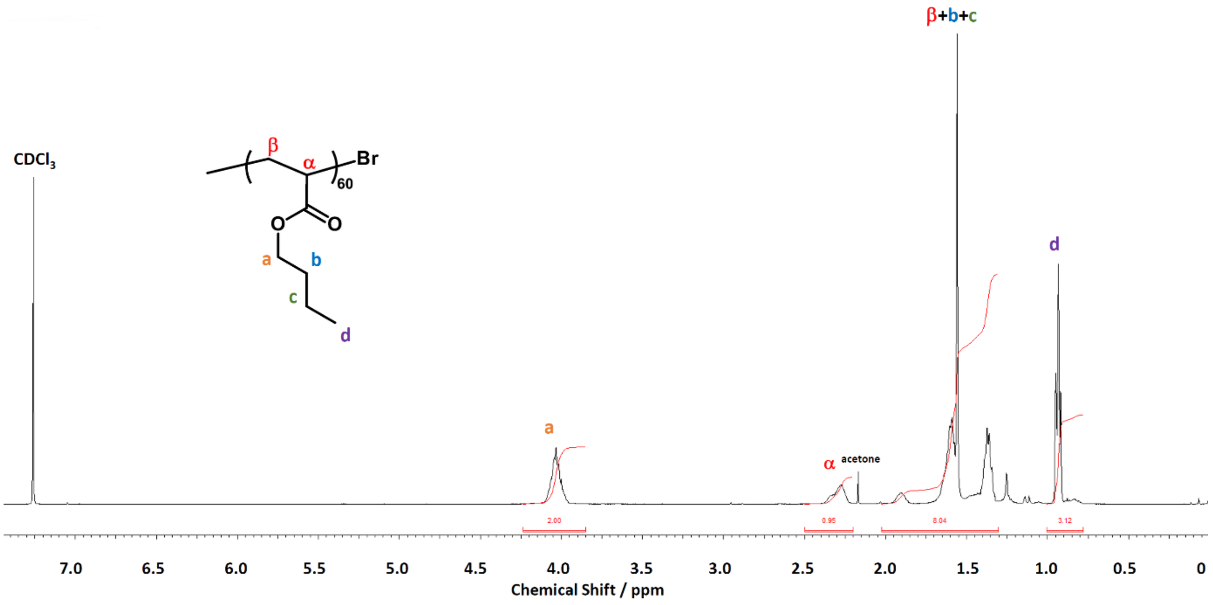

Figure S21. ^{1}H NMR spectrum of P*n*BA (after purification) in $CDCl_3$ (M_n = 7900 g/mol, M_w/M_n = 1.10, **Table S1, entry 2**).

The chemical shifts characteristic for **P*t*BA** were assigned: δ (ppm) = 1.27–1.90 (11H, CH_3–, –CH_2–, a + β, respectively) and 2.17–2.32 (1H, –CH–, α) (**Figure S22**).[4]

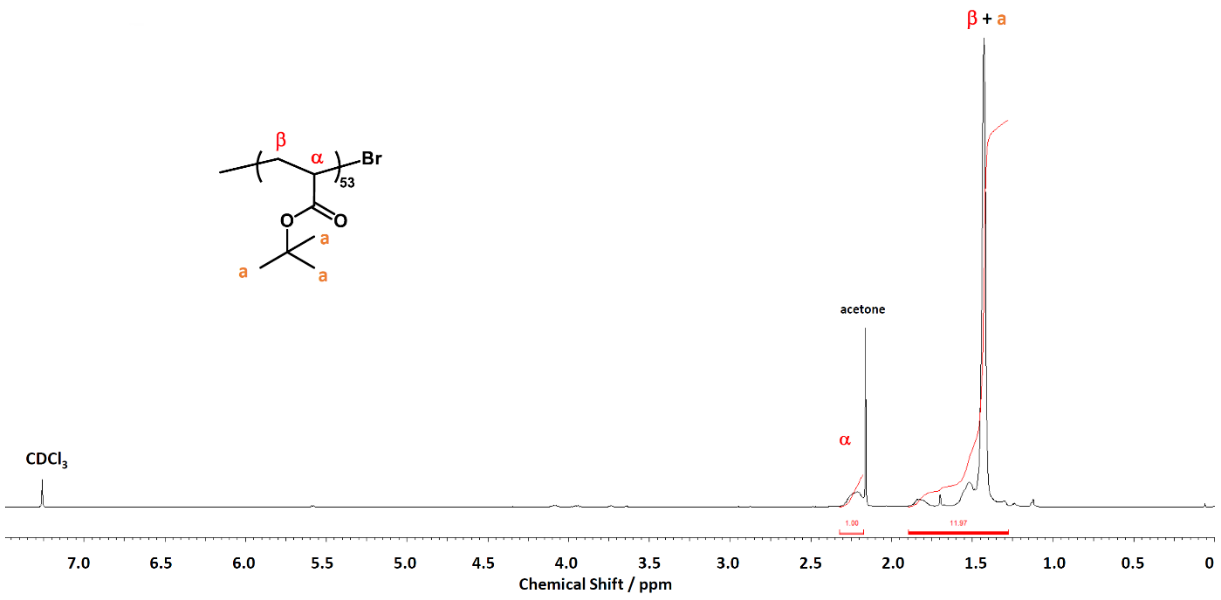

Figure S22. ^{1}H NMR spectrum of P*t*BA (after purification) in $CDCl_3$ (M_n = 7000 g/mol, M_w/M_n = 1.14, **Table S1, entry 3**).

The structures of polymers formed in Cyrene™ were also confirmed by ^{1}H NMR analysis. The chemical shifts characteristic for **P*n*BA** were assigned: δ (ppm) = 0.80–1.11 (3H, CH_3–, d), 1.33–2.11 (6H, CH_2–, β+b+c), 2.14–2.55 (1H, –CH–, α), 3.85–4.29 (2H, CH_2–, a) (**Figure S23**).[4]

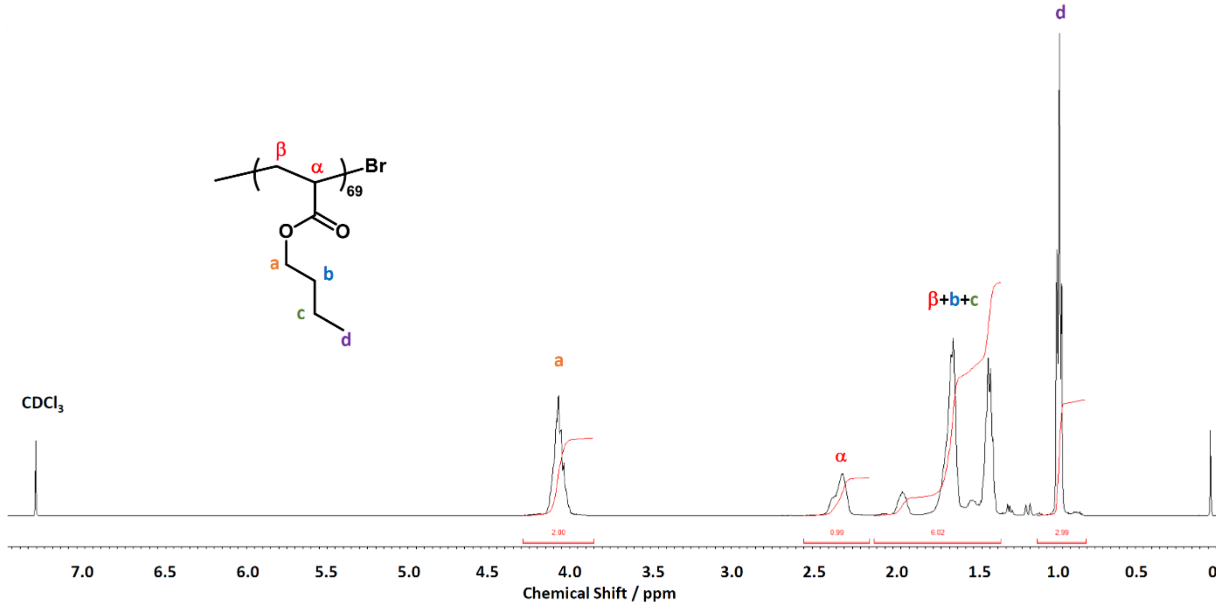

Figure S23. ^{1}H NMR spectrum of P*n*BA (after purification) in $CDCl_3$ (M_n = 6200 g/mol, M_w/M_n = 1.15, **Table 1, entry 4**).

The chemical shifts characteristic for **P*t*BA** were assigned: δ (ppm) = 1.25–1.87 (11H, CH_3–, –CH_2–, a + β, respectively) and 2.15–2.32 (1H, –CH–, α) (**Figure S24**).[4]

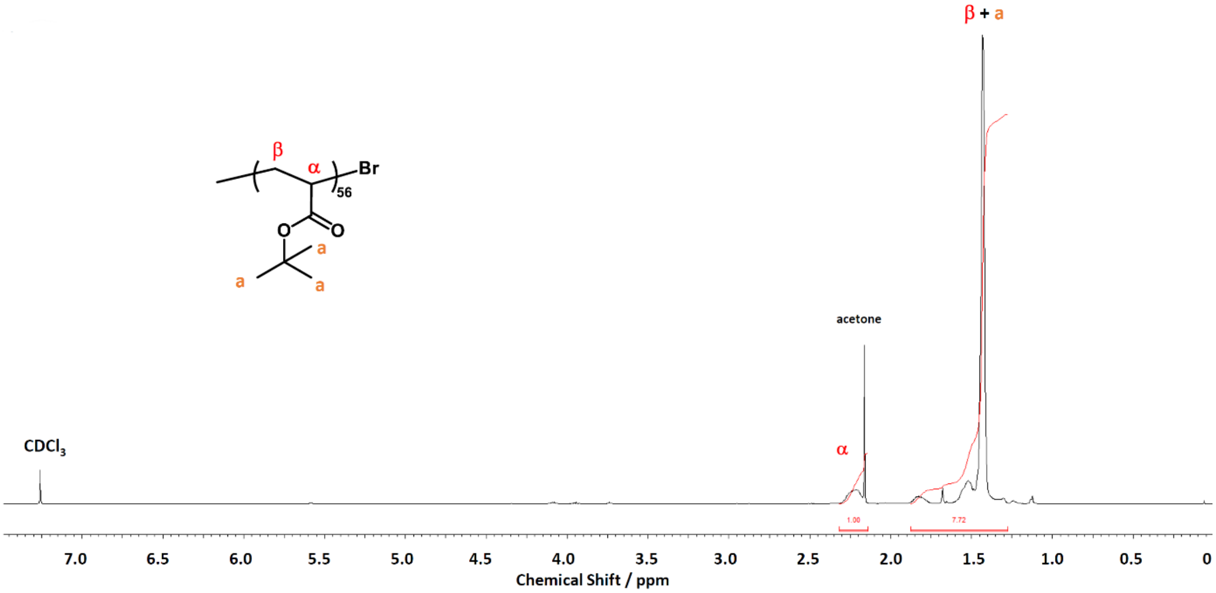

Figure S24. ^{1}H NMR spectrum of P*t*BA (after purification) in $CDCl_3$ (M_n = 7400 g/mol, M_w/M_n = 1.15, **Table S3, entry 2**).

The chemical shifts characteristic for **PHEA** structure were assigned: δ (ppm) = 1.34–1.97 (2H, –C*H*2–, β), 2.12–2.48 (1H, –C*H*–, α), 3.49–3.66 (2H, –C*H*2–,a), 3.92–4.17 (2H, –C*H*2–,b) and 4.61–4.90 (1H, –OH, c) (**Figure S25**).[13]

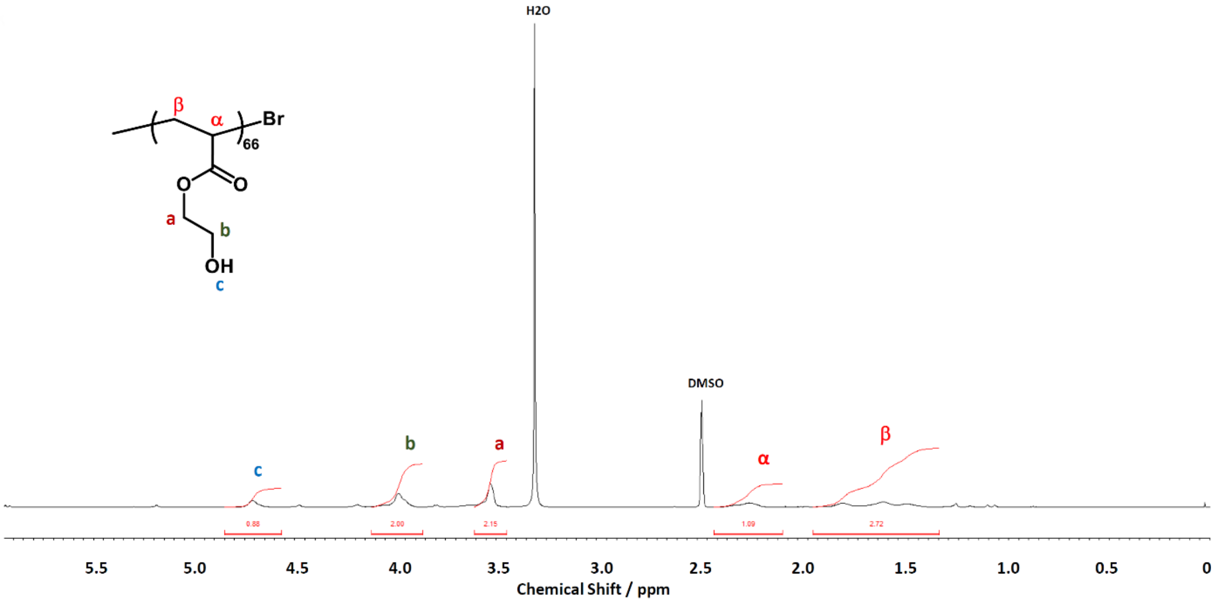

Figure S25. ^{1}H NMR spectrum of PHEA (after purification) in DMSO-d_6 (M_n = 12200 g/mol, M_w/M_n = 1.36, **Table S3, entry 3**).

The chemical shifts characteristic for **POEGA** were assigned: δ (ppm) = 1.34–2.09 (2H, –CH_2–, β), 2.19–2.48 (1H, –C*H*–, α), 3.30–3.38 (3H, –CH_3, c), 3.47–3.77 (2H, –CH_2–, a + b) and 4.01–4.36 (2H, –CH_2–, a in the first segment) (**Figure S26**).[14]

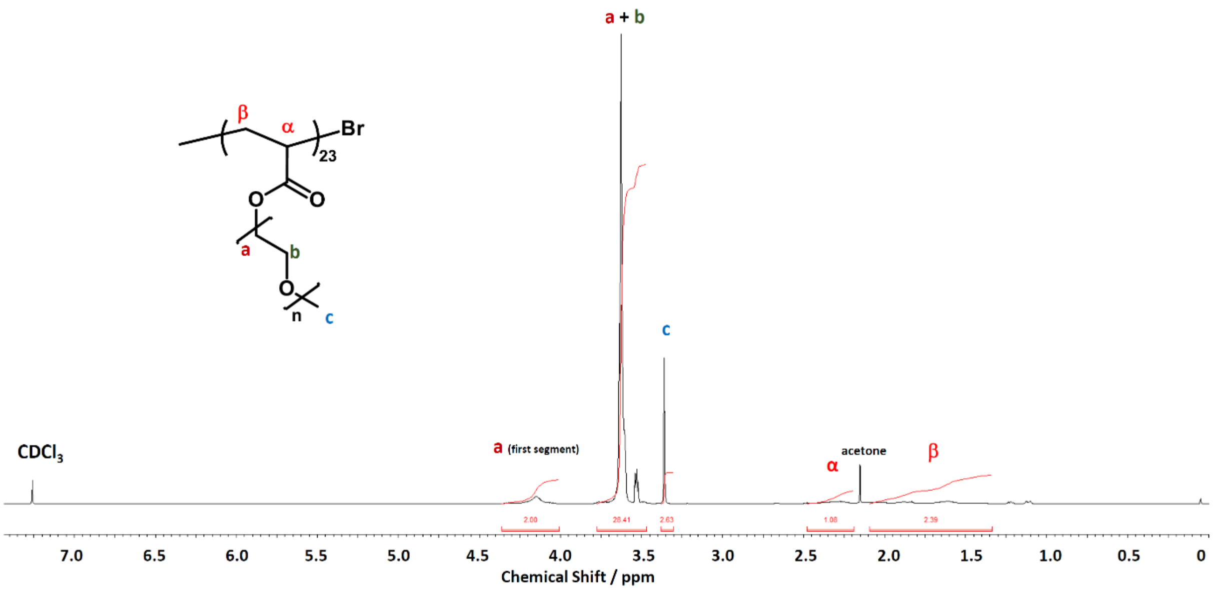

Figure S26. ^{1}H NMR spectrum of POEGA (after purification) in $CDCl_3$ (M_n = 11400 g/mol, M_w/M_n = 1.23, **Table S3, entry 4**).

The chemical shifts characteristic for **PMMA** were assigned: δ (ppm) = 0.76–1.11 (3H, CH_3–, α), 1.74–2.13 (2H, –CH_2–, β) and 3.39–3.87 (3H, CH_3–, a) (**Figure S27**).[15]

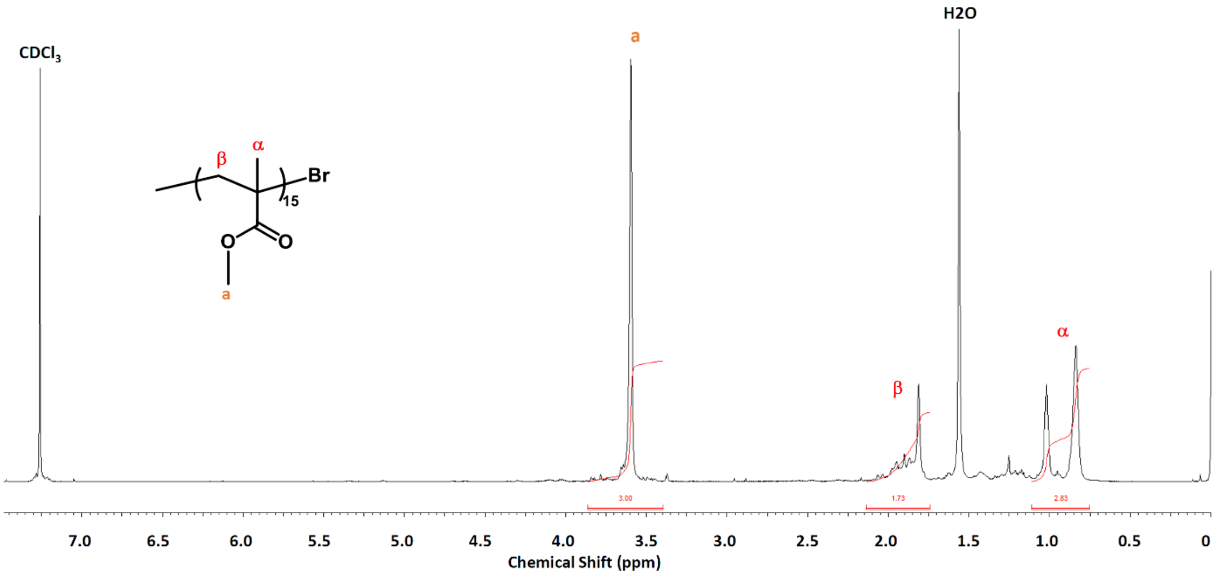

Figure S27. ^{1}H NMR spectrum of PMMA (after purification) in $CDCl_3$ (M_n = 1700 g/mol, M_w/M_n = 1.33, **Table S3, entry 5**).

The chemical shifts characteristic for **PHEMA** structure were assigned: δ (ppm) = 0.50–1.23 (2H, –CH_2–, β), 1.58–2.02 (3H, –CH_3, α), 3.51–3.65 (2H, –CH_2–,a), 3.71–4.00 (2H, –CH_2–,b), 4.72–4.89 (1H, –OH, c) (**Figure S28**).[16]

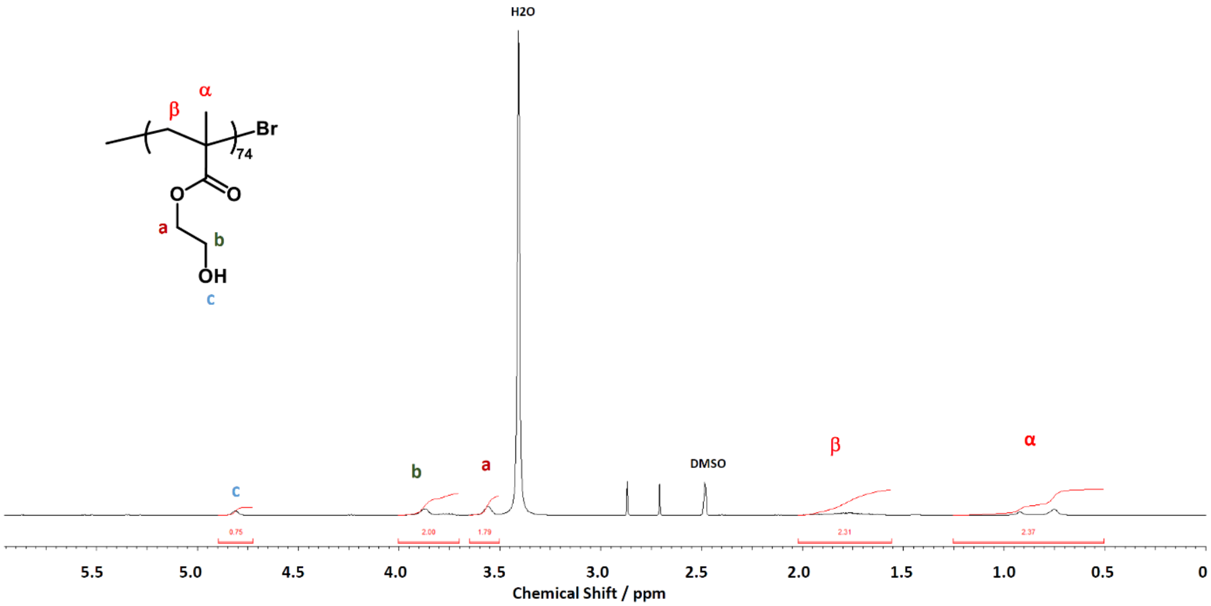

Figure S28. ^{1}H NMR spectrum of PHEMA (after purification) in DMSO-d_6 (M_n = 9900 g/mol, M_w/M_n = 1.47, **Table S3, entry 6**).

The chemical shifts characteristic for **PGMA** structure were assigned: δ (ppm) = 0.73–1.17 (2H, –CH_2–, β), 1.61–2.20 (3H, –CH_3, α), 2.59-2.95 (2H, –CH_2–,c), 3.18–3.33 (1H, –CH–,), 3.62–4.22 (2H, –CH_2–,a), 4.25–4.46 (2H, –CH_2–, a) (**Figure S29**).[17]

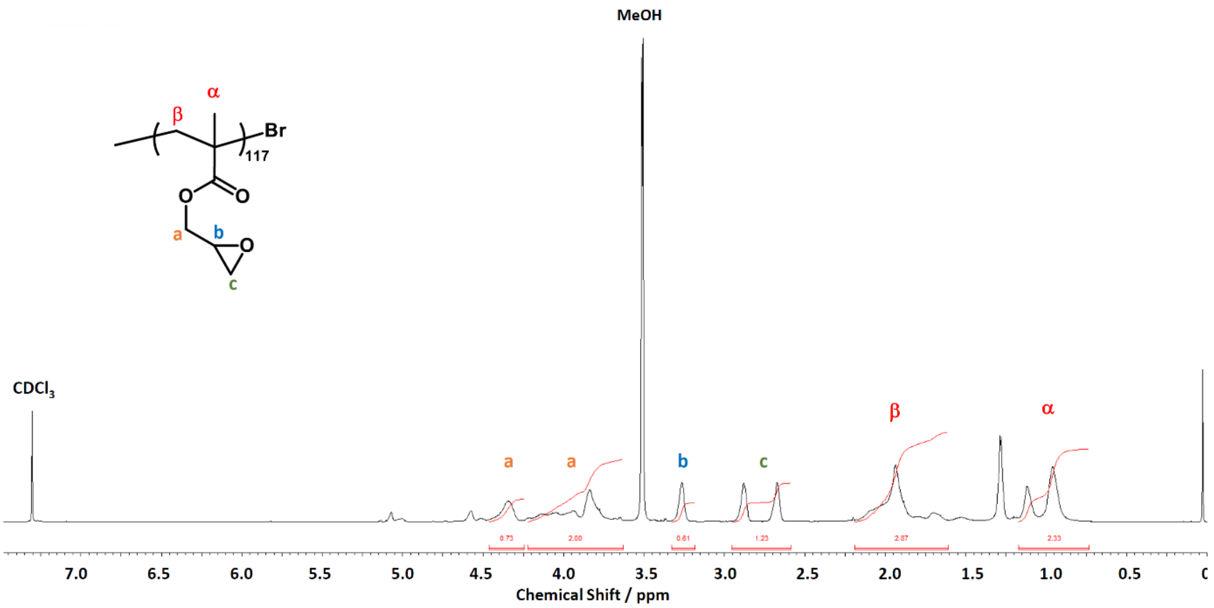

Figure S29. ^{1}H NMR spectrum of PGMA (after purification) in $CDCl_3$ (M_n = 16900 g/mol, M_w/M_n = 1.43, **Table S3, entry 7**).

The chemical shifts identified in the ^{1}H NMR spectrum of **PDMAEMA:** δ (ppm) = 0.80–1.20 (3H, CH_3–, α), 1.75–2.17 (2H, –CH_2–, β), 2.29–2.57 (6H, $N(CH_3)_2$, c), 2.58–3.02 (2H, –CH_2–, b) and 4.04–4.45 (2H, –CH_2–, a) (**Figure S30**).[18]

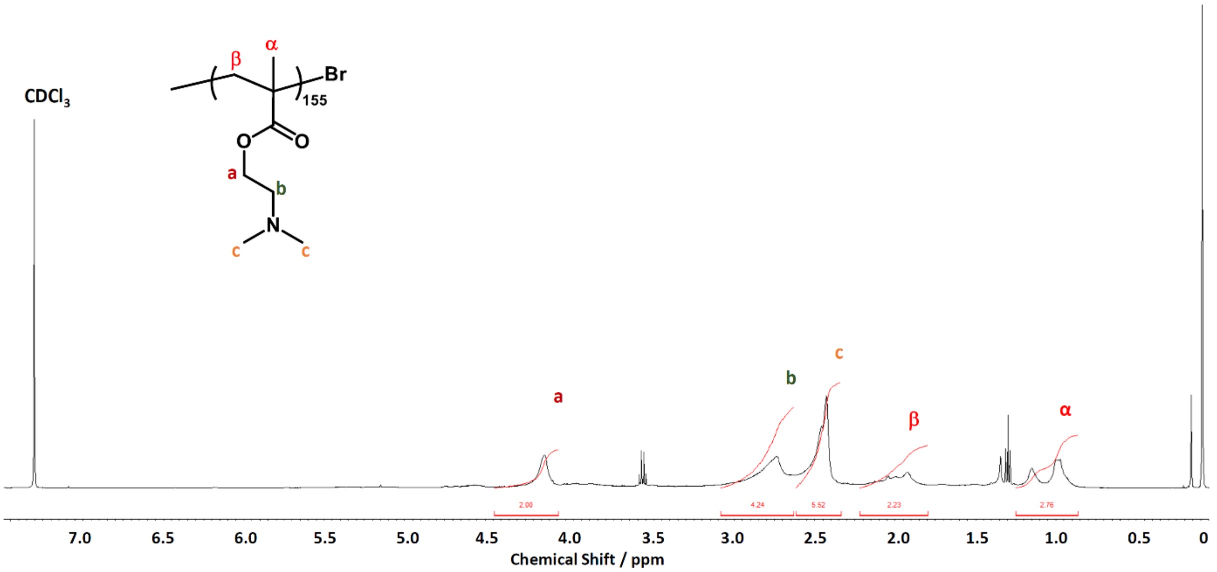

Figure S30. ^{1}H NMR spectrum of PDMAEMA (after purification) in $CDCl_3$ (M_n = 17800 g/mol, M_w/M_n = 1.50, **Table S3, entry 8**).

Figure S31 shows ^{1}H NMR spectrum for P*n*BA-*b*-P*t*BA block copolymer formed in Cyrene™. The chemical shifts for *n*BA and *t*BA units were assigned: δ (ppm) = 0.78–0.99 (3H, CH_3–, d), 1.19–1.97 (15H, –CH_2–CH_2–, –CH_2–, β+b+c+e), 2.19–2.43 (1H, –CH–, α) and 3.77–4.31 (2H, –OCH_2–, a).[4, 19]

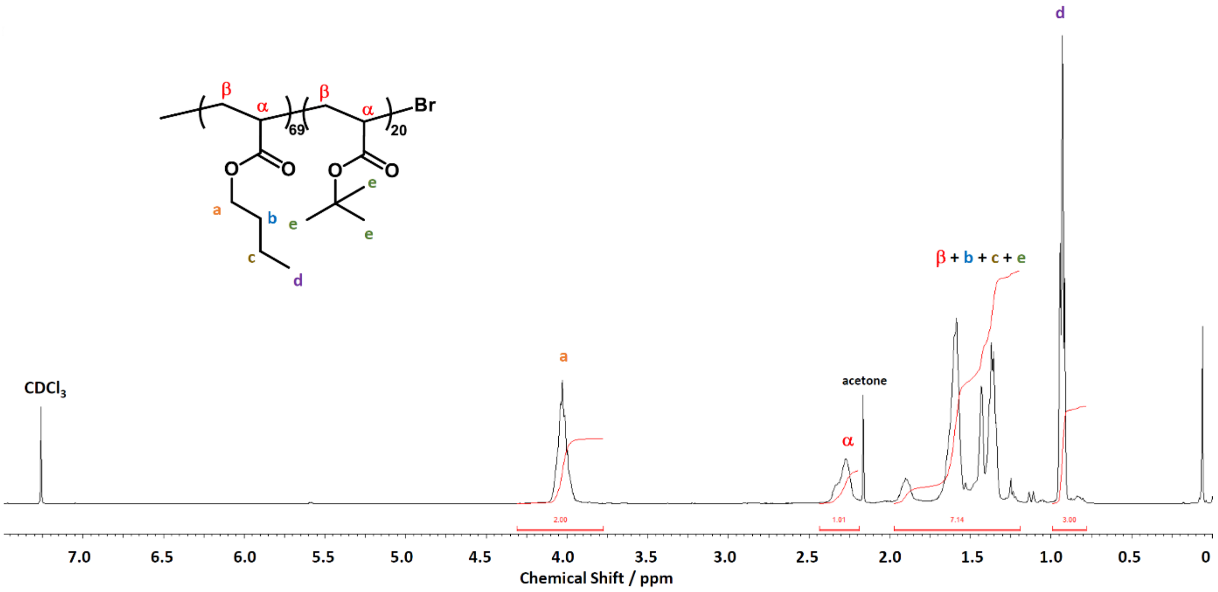

Figure S31. ^{1}H NMR spectrum of P*n*BA-*b*-P*t*BA (after purification) in $CDCl_3$ (M_n = 8900 g/mol, M_w/M_n = 1.11, **Table S4, entry 1**).

The structures of polymers with branched architecture formed in Cyrene™ were also confirmed by ^{1}H NMR analysis. The chemical shifts characteristic for **P*n*BA** forming side chains of RF-*g*-(P*n*BA-Br)$_2$, Trox-*g*-(P*n*BA-Br)$_{10}$, and β-CD-*g*-(P*n*BA-Br)$_{15}$, respectively, were assigned as follows: δ (ppm) = 0.72–1.11 (3H, CH_3–, d), 1.18–1.98 (6H, CH_2–, β+b+c), 2.14–2.48 (1H, –C*H*–, α), 3.74–4.25 (2H, CH_2–, a) (**Figure S32**);[4] δ (ppm) = 0.78–0.85 (3H, CH_3–, d), 1.18–2.07 (6H, CH_2–, β+b+c), 2.08–2.55 (1H, –C*H*–, α), 3.79–4.28 (2H, CH_2–, a) (**Figure S33**); δ (ppm) =

0.75–1.09 (3H, CH_3–, d), 1.20–1.99 (6H, CH_2–, β+b+c), 2.10–2.70 (1H, –CH–, α), 3.72–4.31 (2H, CH_2–, a) (**Figure S34**).

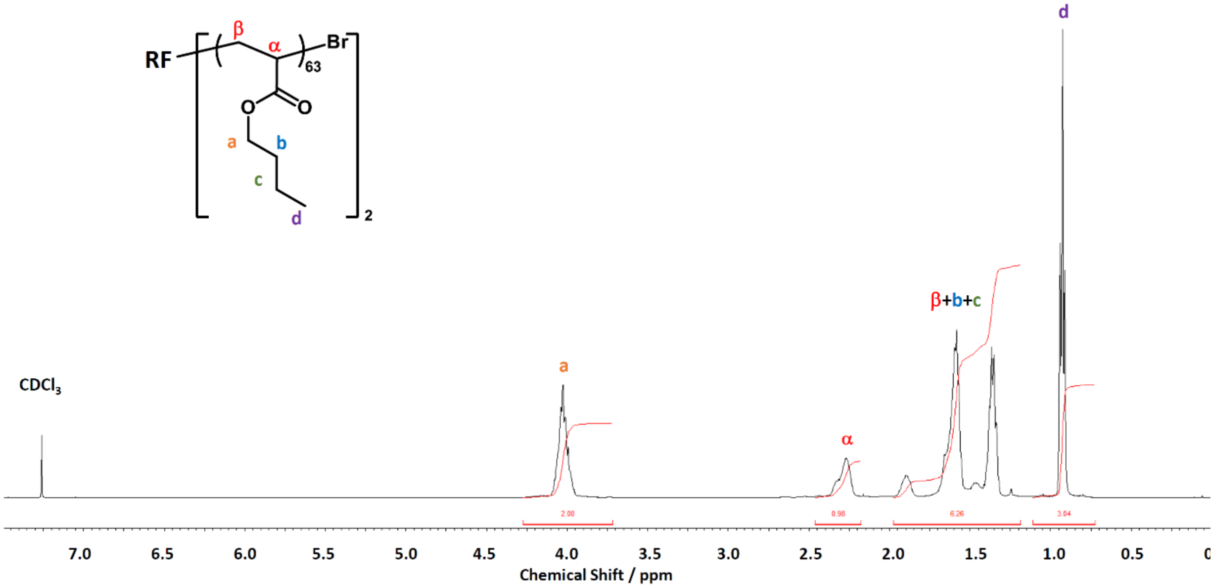

Figure S32. ^{1}H NMR spectrum of RF-*g*-(P*n*BA-Br)$_2$ (after purification) in $CDCl_3$ (M_n = 13800 g/mol, M_w/M_n = 1.24, **Table 2, entry 1**).

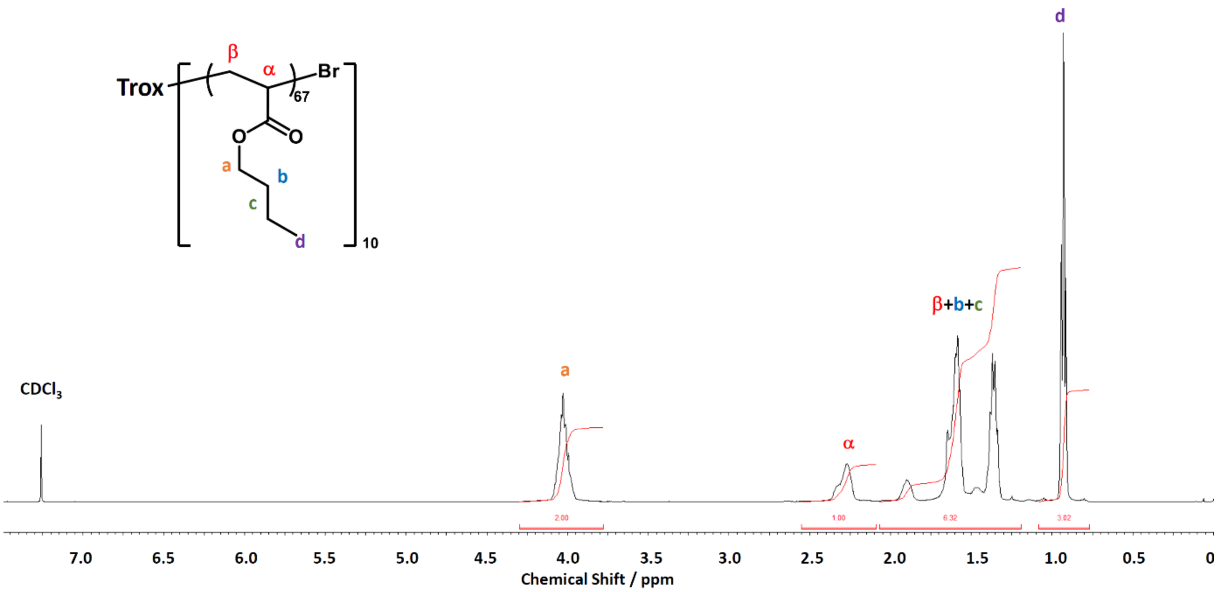

Figure S33. ^{1}H NMR spectrum of Trox-*g*-(P*n*BA-Br)$_{10}$ (after purification) in $CDCl_3$ (M_n = 35800 g/mol, M_w/M_n = 1.37, **Table 2, entry 2**).

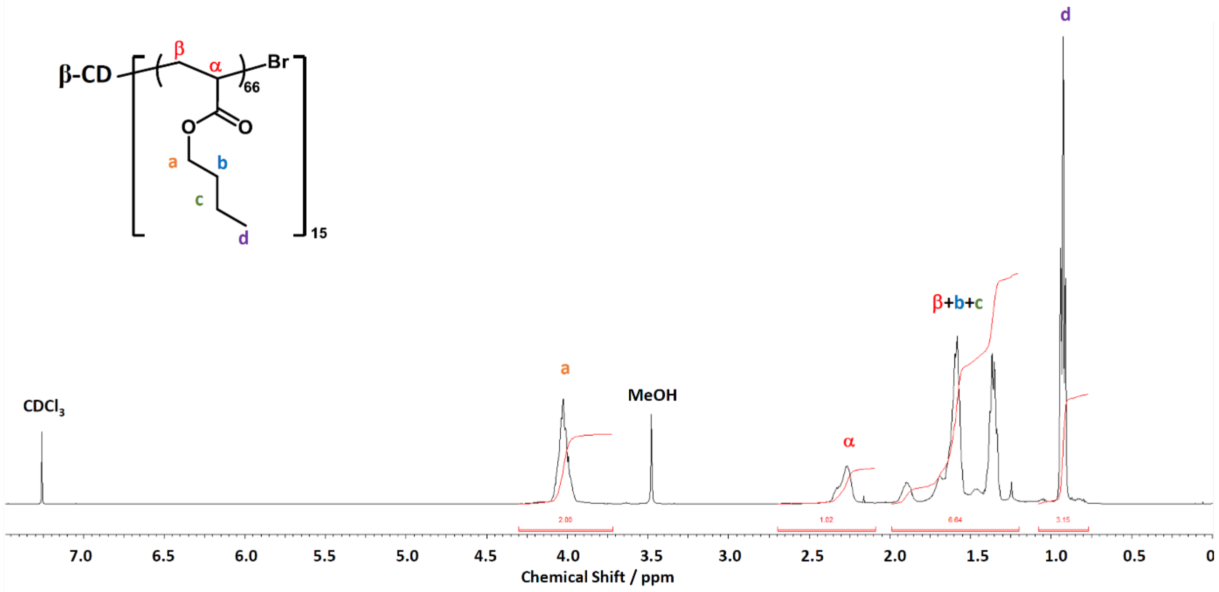

Figure S34. ^{1}H NMR spectrum of β-CD-*g*-(P*n*BA-Br)$_{15}$ (after purification) in $CDCl_3$ (M_n = 29300 g/mol, M_w/M_n = 1.54, **Table 2, entry 3**).

Spectroscopic analysis was also applied to confirm the structures of the polymers synthesized in Cygnet 0.0. The chemical shifts characteristic for **P*n*BA** structure were assigned as follows: δ (ppm) = 0.77–1.07 (3H, CH_3–, d), 1.29–1.96 (6H, CH_2–, β+b+c), 2.19–2.62 (1H, –C*H*–, α), 3.79–4.30 (2H, CH_2–, a) (**Figure S35**). [4]

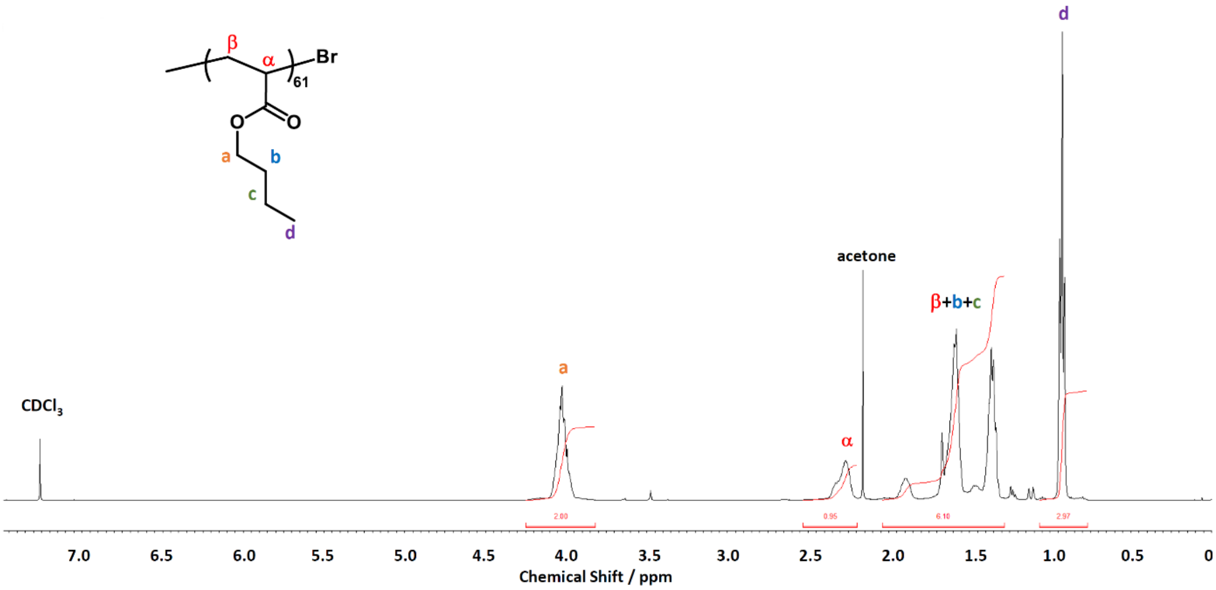

Figure S35. ^{1}H NMR spectrum of P*n*BA (after purification) in $CDCl_3$ (M_n = 8010 g/mol, M_w/M_n = 1.14, **Table 3, entry 3**).

The chemical shifts characteristic for **P*t*BA** structure were assigned as follows: δ (ppm) = 1.24–1.91 (11H, CH_3–, –CH_2–, a + β, respectively) and 2.12–2.32 (1H, –CH–, α) (**Figure 36**).[4]

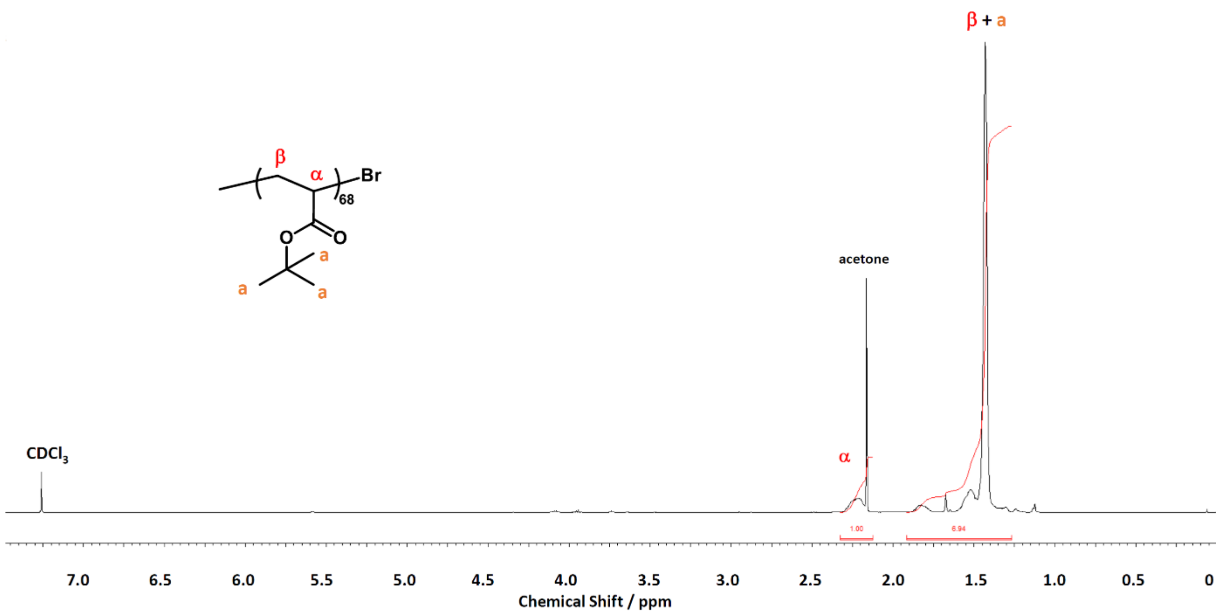

Figure S36. ^{1}H NMR spectrum of P*t*BA (after purification) in $CDCl_3$ (M_n = 8900 g/mol, M_w/M_n = 1.21, **Table 3, entry 2**).

The chemical shifts characteristic for **PHEA** structure were assigned: δ (ppm) = 1.30–2.03 (2H, –CH_2–, β), 2.15–2.44 (1H, –C*H*–, α), 3.13–3.58 (2H, –CH_2–,a), 3.74–4.32 (2H, –CH_2–,b) and 4.60-4.87 (1H, –OH, c) (**Figure S37**).[13]

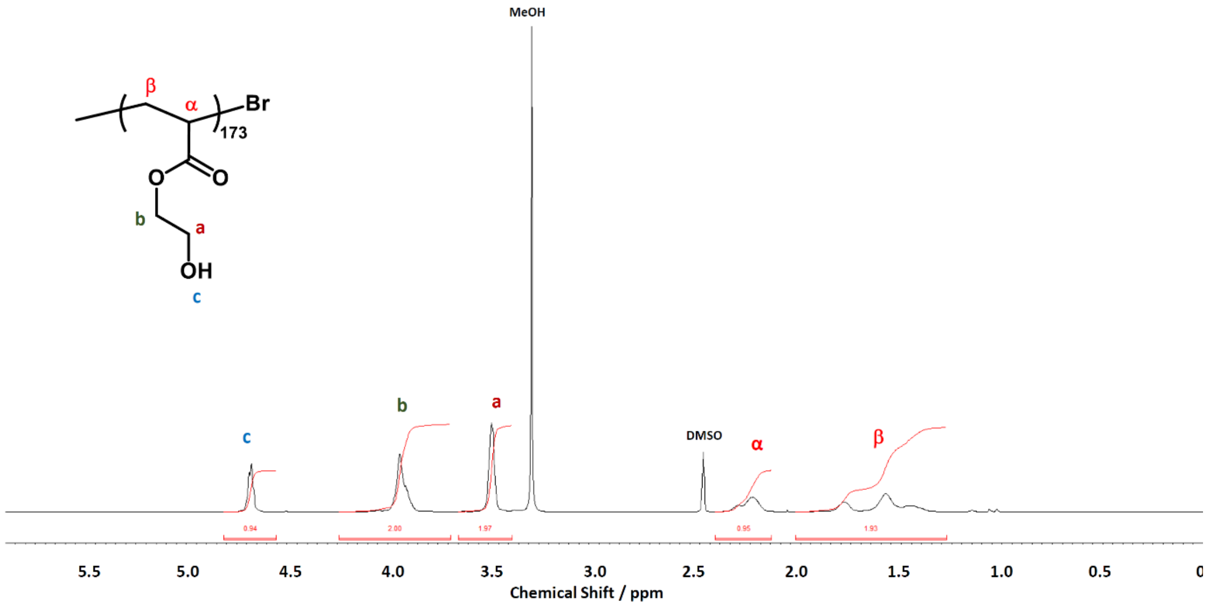

Figure S37. ^{1}H NMR spectrum of PHEA (after purification) in DMSO-d_6 (M_n = 22400 g/mol, M_w/M_n = 1.36, **Table S5, entry 2**).

The chemical shifts characteristic for **PMMA** were assigned: δ (ppm) = 0.75–1.11 (3H, CH_3–, α), 1.62–2.13 (2H, –CH_2–, β) and 3.45–3.76 (3H, CH_3–, a) (**Figure S38**).[15]

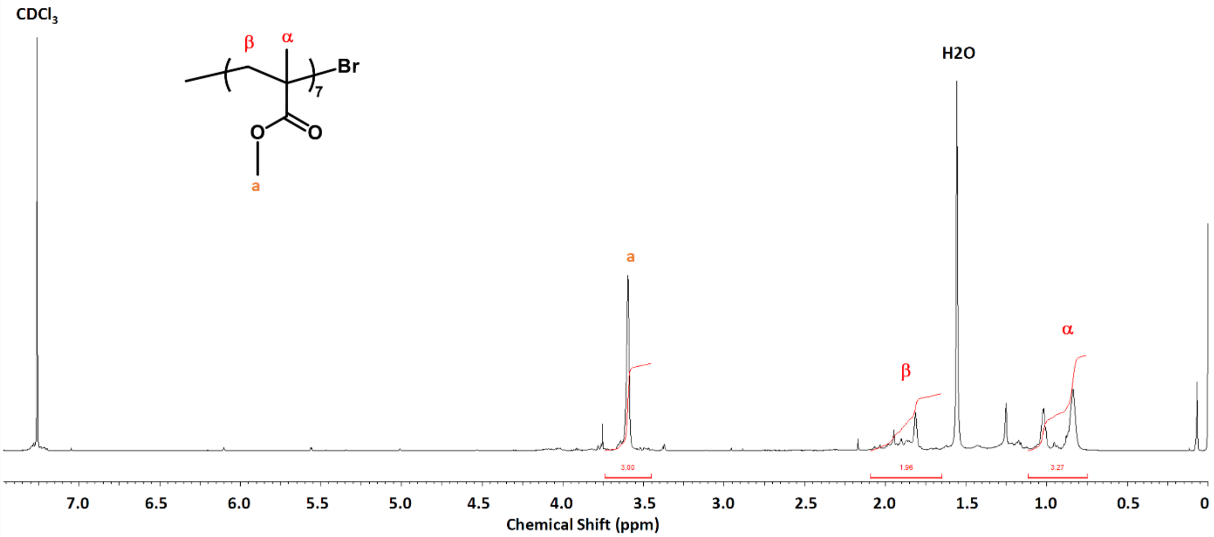

Figure S38. ^{1}H NMR spectrum of PMMA (after purification) in $CDCl_3$ (M_n = 980 g/mol, M_w/M_n = 1.38, **Table S5, entry 3**).

The chemical shifts characteristic for **PHEMA** structure were assigned: δ (ppm) = 0.62–1.15 (2H, –CH_2–, β), 1.63–2.11 (3H, –CH_3, α), 3.50–3.70 (2H, –CH_2–,a), 3.76–4.12 (2H, –CH_2–,b), 4.77–4.89 (1H, –OH, c) (**Figure S39**).[16]

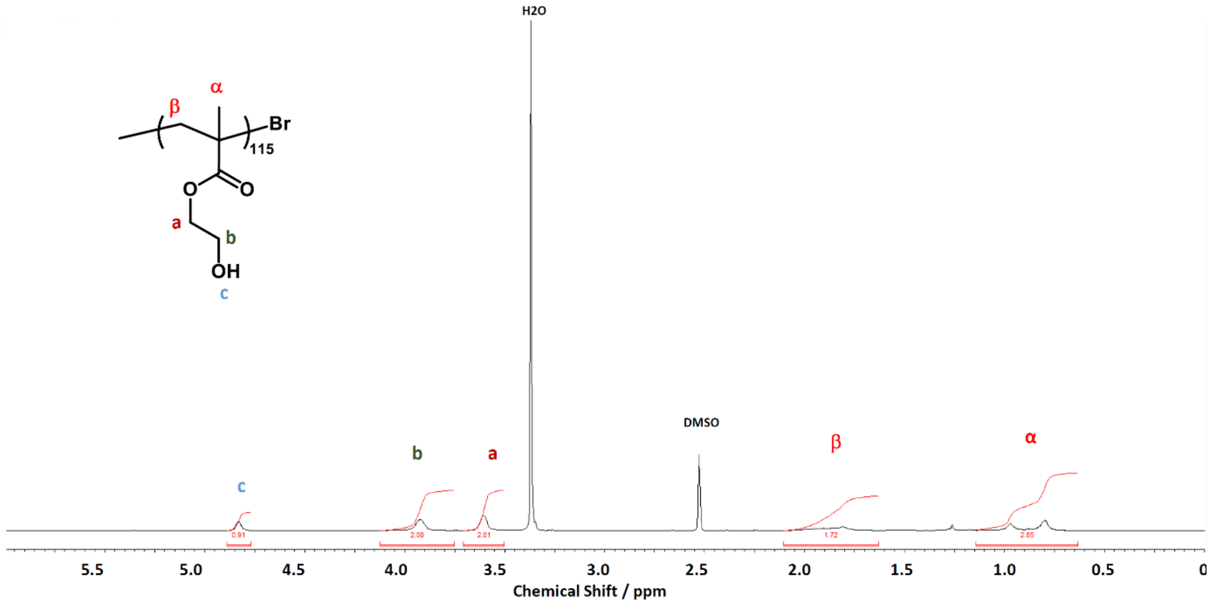

Figure S39. ^{1}H NMR spectrum of PHEMA (after purification) in DMSO-d_6 (M_n = 15300 g/mol, M_w/M_n = 1,87, **Table S5, entry 4**).

The structures of polymers with branched architectures formed in Cyrene™ were further confirmed through ^{1}H NMR analysis. The chemical shifts corresponding to the **P*n*BA** forming the side chains of RF-*g*-(P*n*BA-Br)$_2$, Trox-*g*-(P*n*BA-Br)$_{10}$, and β-CD-*g*-(P*n*BA-Br)$_{15}$ were assigned as follows: δ (ppm) = 0.79–1.16 (3H, CH_3–, d), 1.19–2.12 (6H, CH_2–, β+b+c), 2.15–2.65 (1H, –C*H*–, α), 3.80–4.34 (2H, CH_2–, a) (**Figure S40**);[4] δ (ppm) = 0.76–1.08 (3H, CH_3–, d), 1.31–1.98 (6H, CH_2–, β+b+c), 2.16–2.49 (1H, –C*H*–, α), 3.80–4.29 (2H, CH_2–, a) (**Figure S41**);[4] δ (ppm) = 0.78–1.13 (3H, CH_3–, d), 1.31–2.08 (6H, CH_2–, β+b+c), 2.19–2.59 (1H, –C*H*–, α), 3.77–4.35 (2H, CH_2–, a) (**Figure S42**).[4]

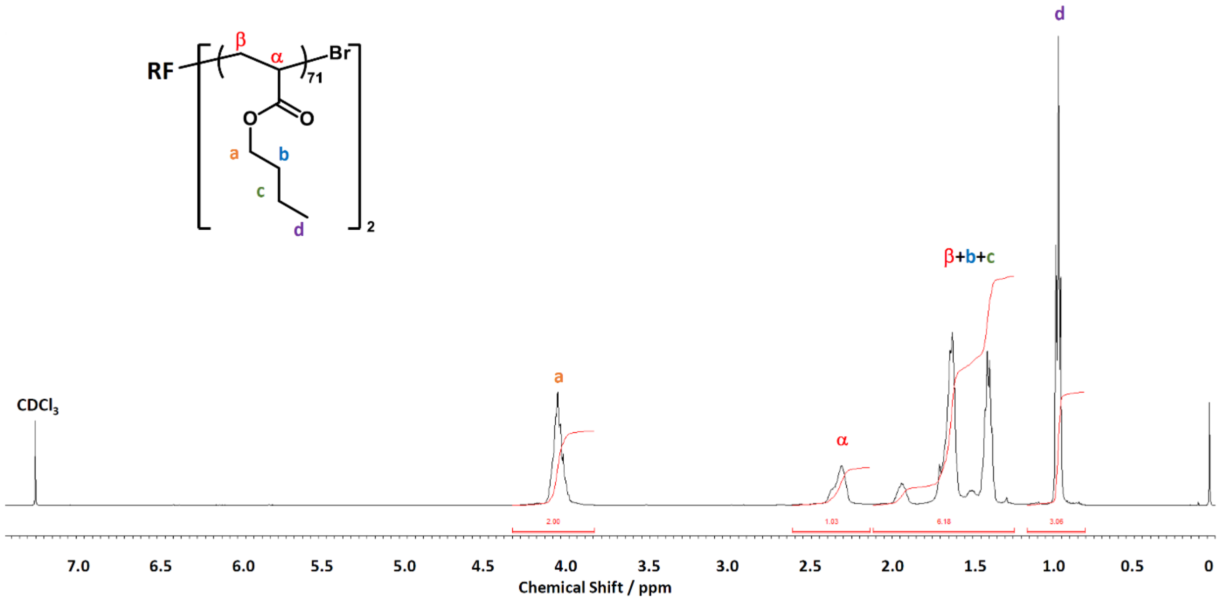

Figure S40. ^{1}H NMR spectrum of RF-*g*-(P*n*BA-Br)$_2$ (after purification) in $CDCl_3$ (M_n = 15900 g/mol, M_w/M_n = 1.31, **Table 4, entry 1**).

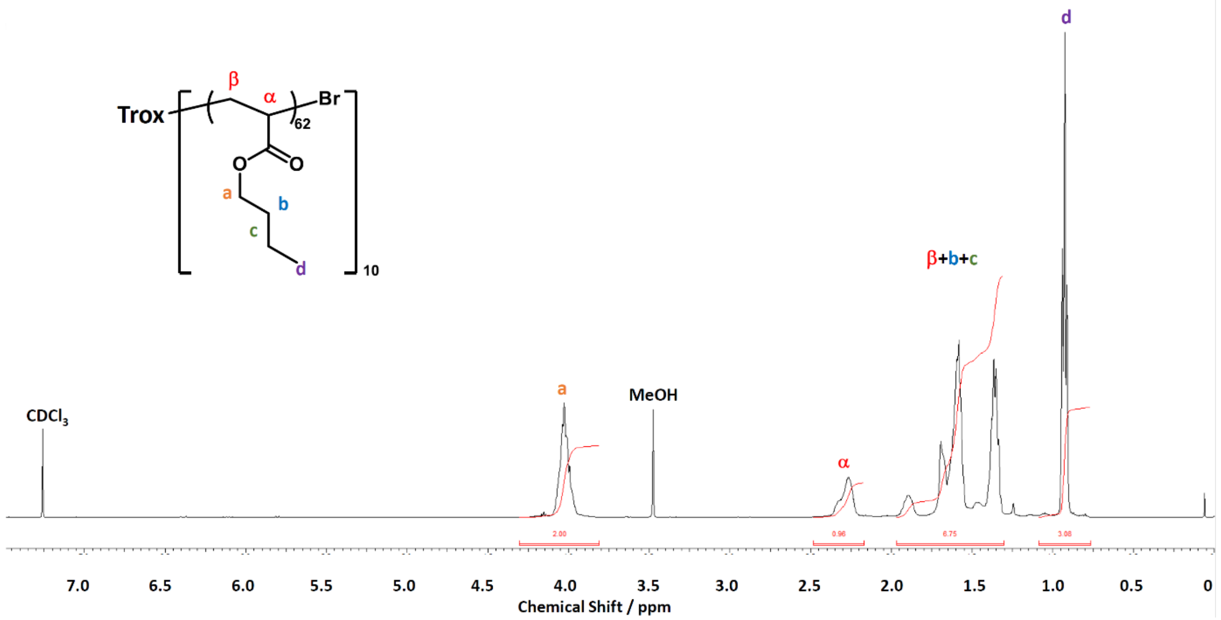

Figure S41. ^{1}H NMR spectrum of Trox-*g*-(P*n*BA-Br)$_{10}$ (after purification) in $CDCl_3$ (M_n = 33700 g/mol, M_w/M_n = 1.37, **Table 4, entry 2**).

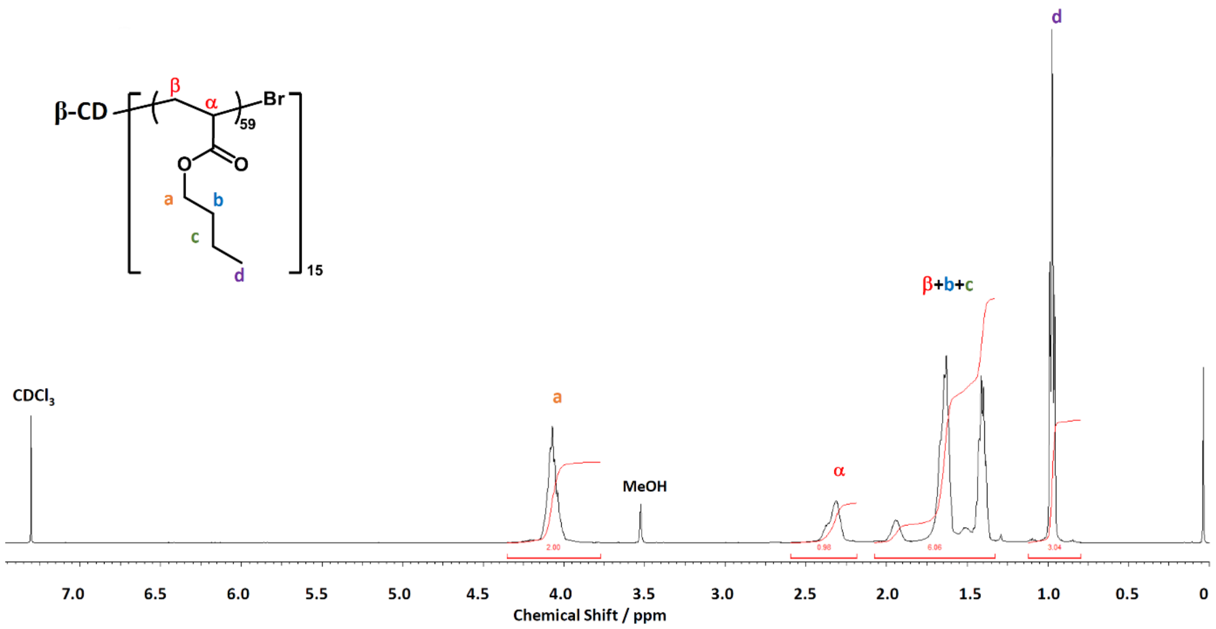

Figure S42. ^{1}H NMR spectrum of β-CD-*g*-(P*n*BA-Br)$_{15}$ (after purification) in $CDCl_3$ (M_n = 37100 g/mol, M_w/M_n = 1.41, **Table 4, entry 3**).

S11. E-factor and effective mass yield (EMY)

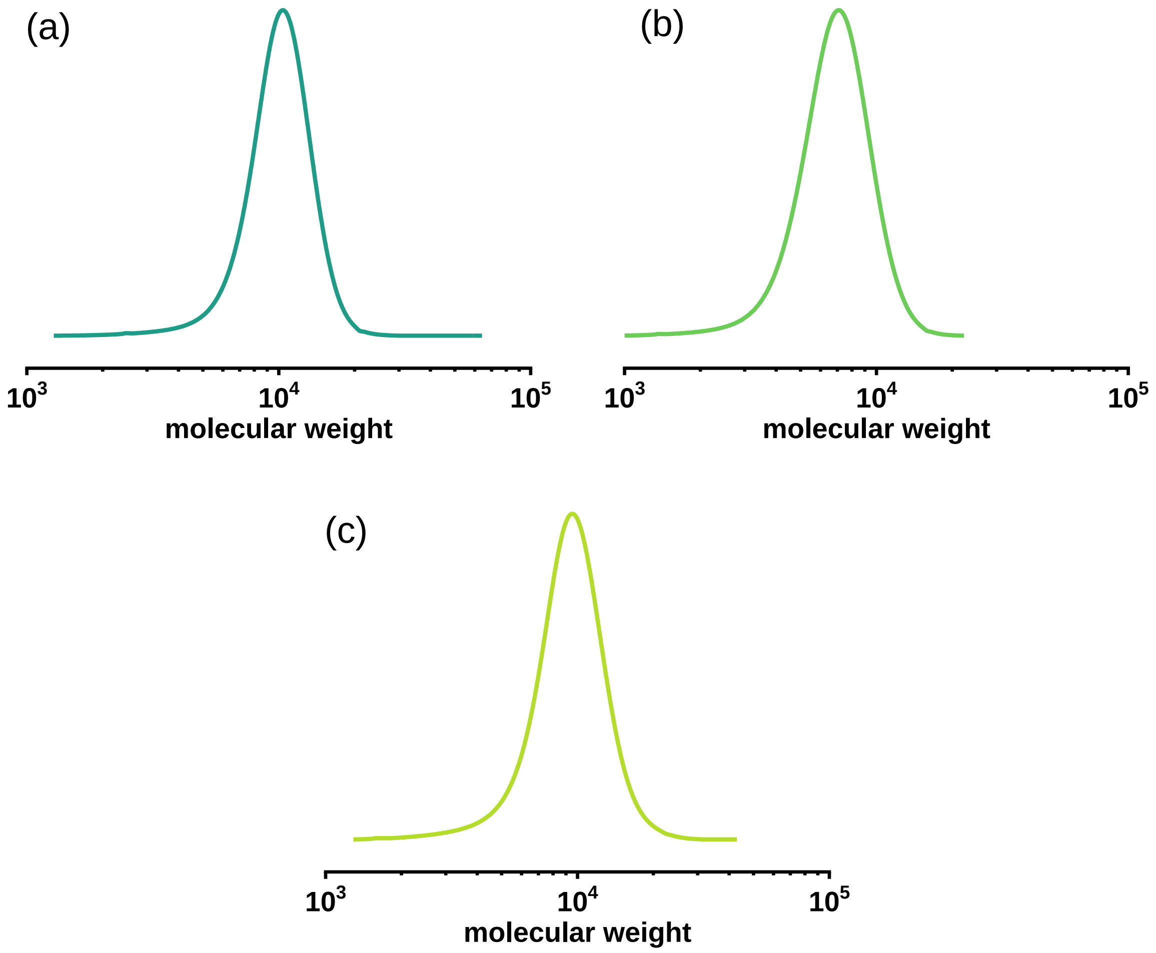

Figure S43. GPC traces of P*n*BA synthesized in (a) DMF, (b) Cyrene™, and (c) Cygnet 0.0 (**Table 5**).

Table S6 . Polymerization of *n*BA in DMF, Cyrene™ and CYGNET 0.0 *via* SARA ATRP.

Entry (according to Table 5)	E-factor$^{theo\ a}$ [-]	E-factor$^{app\ b}$ [-]	
		dialysis	precipitation
**1**	1.50	1.52	1.53
**2**	1.82	2.01	1.92
**3**	1.33	1.37	2.10

[a]E-factor^{theo}, theoretical environmental factor calculated as a ratio of the mass of waste per mass of product, where the mass of waste is a sum of the masses of catalyst, ligand, unreacted monomer, and solvent, and the mass of the product was calculated based on monomer conversion calculated from NMR analysis.

[b]E-factor^{app}, apparent environmental factor calculated as a ratio of the mass of waste per mass of product, where the mass of waste is a sum of the masses of catalyst, ligand, unreacted monomer, and solvent (in the case of column "with solvent"), and the mass of the product was actual mass of weighed polymer after dialysis or precipitation.

S12. Determination of copper concentration in final polymer samples by atomic absorption spectrometry (AAS)

The AAS technique is a commonly employed method for quantifying metal residues in the food and pharmaceutical industries. In this context, it was utilized to assess the copper concentration remaining in the polymer after purification by dialysis or precipitation from an appropriate solvent mixture. The isolated polymer, synthesized as per Table 5, underwent decomposition in a drying oven and subsequent mineralization within a microwave digestion system. This process left behind only the copper content, which was atomized in a flame during the measurement. The concentration was determined through the use of the equation illustrated in **Figure S44**:

$$c_{Cu} = \frac{A}{0{,}05278}$$

where A – absorbance [-], c_{Cu} – copper concentration [mg/L].

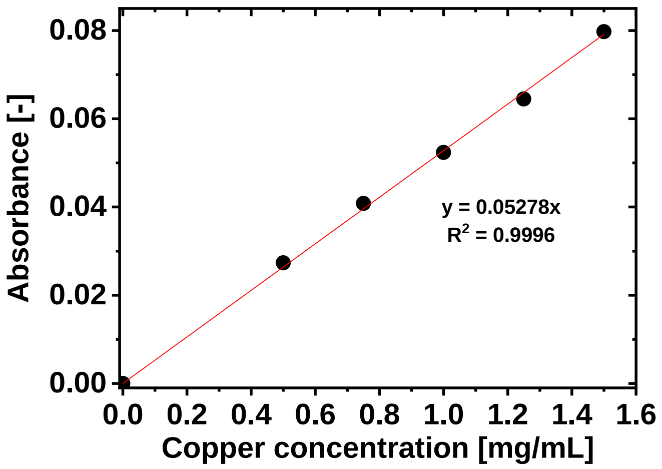

Figure S44. The calibration curve established by analyzing $Cu^{II}Br_2$ reference solutions in distilled water by AAS with a copper hollow cathode lamp, measurements were taken at a wavelength of 324.8 nm, and a flame atomizer.

The copper concentration in the final polymer samples after purification determined by AAS based on the calibration curve was summarized in **Table S7**.

Table S7 . Copper concentration in the final polymer sample determined by AAS.

**Entry (according to Table 5)**	**Copper concentration [ppm]**	
	**dialysis**	**precipitation**
**1**	119	439
**2**	43	8
**3**	46	209

The contamination of final polymer products with metal is a critical concern, especially when considering the applications of these materials in medicine. In terms of the compatibility of the resultant polymer materials with orally administered pharmaceutical preparations, it is essential to consider the guidelines established by The International Council for Harmonization of Technical Requirements for Pharmaceuticals for Human Use (ICH).[20] According to these guidelines, the permissible daily exposure to copper when administered orally is 3400 μg/day, calculated for an individual weighing 50 kg. In the context of 1g of polymer, this indicated dose equates to 3400 ppm. Consequently, the resulting polymeric materials hold significant potential for applications in the field of pharmacy.

References

(1) Sherwood, J.; De bruyn, M.; Constantinou, A.; Moity, L.; McElroy, C. R.; Farmer, T. J.; Duncan, T.; Raverty, W.; Hunt, A. J.; Clark, J. H. Dihydrolevoglucosenone (Cyrene) as a bio-based alternative for dipolar aprotic solvents. *Chem. Comm.* **2014**, *50* (68), 9650-9652, 10.1039/C4CC04133J. DOI: https://doi.org/10.1039/C4CC04133J.

(2) Warne, C. M.; Fadlallah, S.; Whitwood, A. C.; Sherwood, J.; Mouterde, L. M. M.; Allais, F.; Guebitz, G. M.; McElroy, C. R.; Pellis, A. Levoglucosenone-derived synthesis of bio-based solvents and polyesters. *Green Chem. Lett. Rev.* **2023**, *16* (1), 2154573. DOI: https://doi.org/10.1080/17518253.2022.2154573.

(3) Zaborniak, I.; Chmielarz, P. Miniemulsion switchable electrolysis under constant current conditions. *Polym. Adv. Technol.* **2020**, *31* (11), 2806-2815. DOI: https://doi.org/10.1002/pat.5007.

(4) Zaborniak, I.; Chmielarz, P.; Matyjaszewski, K. Synthesis of riboflavin-based macromolecules through low ppm ATRP in aqueous media. *Macromol. Chem. Phys.* **2020**, *221* (4), 1900496. DOI: https://doi.org/10.1002/macp.201900496.

(5) Chmielarz, P.; Park, S.; Sobkowiak, A.; Matyjaszewski, K. Synthesis of β-cyclodextrin-based star polymers via a simplified electrochemically mediated ATRP. *Polymer* **2016**, *88*, 36-42. DOI: https://doi.org/10.1016/j.polymer.2016.02.021.

(6) Cohen, N. A.; Tillman, E. S.; Thakur, S.; Smith, J. R.; Eckenhoff, W. T.; Pintauer, T. Effect of the ligand in atom transfer radical polymerization reactions Initiated by photodimers of 9-bromoanthracene. *Macromol. Chem. Phys.* **2009**, *210* (3-4), 263-268. DOI: https://doi.org/10.1002/macp.200800481.

(7) Kaur, A.; Ribelli, T. G.; Schröder, K.; Matyjaszewski, K.; Pintauer, T. Properties and ATRP activity of copper complexes with substituted tris(2-pyridylmethyl)amine-based ligands. *Inorg. Chem.* **2015**, *54* (4), 1474-1486. DOI: https://doi.org/10.1021/ic502484s.

(8) You, L.; Anslyn, E. V. Secondary alcohol hemiacetal formation: An in situ carbonyl activation strategy. *Org. Lett.* **2009**, *11* (22), 5126-5129. DOI: https://doi.org/10.1021/ol9020207.

(9) De bruyn, M.; Sener, C.; Petrolini, D. D.; McClelland, D. J.; He, J.; Ball, M. R.; Liu, Y.; Martins, L.; Dumesic, J. A.; Huber, G. W.; et al. Catalytic hydrogenation of dihydrolevoglucosenone to levoglucosanol with a hydrotalcite/mixed oxide copper catalyst. *Green Chemistry* **2019**, *21* (18), 5000-5007, https://doi.org/10.1039/C9GC00564A. DOI: 10.1039/C9GC00564A.

(10) Milescu, R. A.; Zhenova, A.; Vastano, M.; Gammons, R.; Lin, S.; Lau, C. H.; Clark, J. H.; McElroy, C. R.; Pellis, A. Polymer chemistry applications of Cyrene and its derivative Cygnet 0.0 as safer replacements for polar aprotic solvents. *ChemSusChem* **2021**, *14* (16), 3367-3381. DOI: https://doi.org/10.1002/cssc.202101125.

(11) dos Santos, V. B.; Fava, E. L.; de Miranda Curi, N. S.; Faria, R. C.; Fatibello-Filho, O. A thermostated electrochemical flow cell with a coupled bismuth film electrode for square-wave anodic stripping voltammetric determination of cadmium(II) and lead(II) in natural, wastewater and tap water samples. *Talanta* **2014**, *126*, 82-90. DOI: https://doi.org/10.1016/j.talanta.2014.03.015.

(12) Pavan, P.; Lorandi, F.; De Bon, F.; Gennaro, A.; Isse, A. A. Enhancement of the rate of atom tansfer radical polymerization in organic solvents by addition of water: An electrochemical study. *ChemElectroChem* **2021**, *8* (13), 2450-2458. DOI: https://doi.org/10.1002/celc.202100430.

(13) Appukuttan, V. K.; Dupont, A.; Denis-Quanquin, S.; Andraud, C.; Monnereau, C. Mild and efficient bromination of poly(hydroxyethyl acrylate) and its use towards ionic-liquid containing polymers. *Polym. Chem.* **2012**, *3* (10), 2723-2726, 10.1039/C2PY20462B. DOI: 10.1039/C2PY20462B.

(14) Zaborniak, I.; Pieńkowska, N.; Chmielarz, P.; Bartosz, G.; Dziedzic, A.; Sadowska-Bartosz, I. Nitroxide-containing amphiphilic polymers prepared by simplified electrochemically mediated ATRP as candidates for therapeutic antioxidants. *Polymer* **2023**, *273*, 125885. DOI: https://doi.org/10.1016/j.polymer.2023.125885.

(15) Xu, J.; Shanmugam, S.; Duong, H. T.; Boyer, C. Organo-photocatalysts for photoinduced electron transfer-reversible addition–fragmentation chain transfer (PET-RAFT) polymerization. *Polym. Chem.* **2015**, *6* (31), 5615-5624, 10.1039/C4PY01317D. DOI: 10.1039/C4PY01317D.

(16) Hou, C.; Lin, S.; Liu, F.; Hu, J.; Zhang, G.; Liu, G.; Tu, Y.; Zou, H.; Luo, H. Synthesis of poly(2-hydroxyethyl methacrylate) end-capped with asymmetric functional groups via atom transfer radical polymerization. *New J. Chem.* **2014**, *38* (6), 2538-2547, 10.1039/C3NJ01398G. DOI: 10.1039/C3NJ01398G.

(17) Pranantyo, D.; Xu, L. Q.; Neoh, K.-G.; Kang, E.-T.; Yang, W.; Lay-Ming Teo, S. Photoinduced anchoring and micropatterning of macroinitiators on polyurethane surfaces for graft polymerization of antifouling brush coatings. *J. Mater. Chem. B* **2014**, *2* (4), 398-408, 10.1039/C3TB21201G. DOI: 10.1039/C3TB21201G.

(18) Zaborniak, I.; Macior, A.; Chmielarz, P. Smart, naturally-derived macromolecules for controlled drug release. *Molecules* **2021**, *26* (7), 1918. DOI: https://doi.org/10.3390/molecules26071918.

(19) Xie, Y.; Moreno, N.; Calo, V. M.; Cheng, H.; Hong, P.-Y.; Sougrat, R.; Behzad, A. R.; Tayouo, R.; Nunes, S. P. Synthesis of highly porous poly(*tert*-butyl acrylate)-*b*-polysulfone-*b*-poly(*tert*-butyl acrylate) asymmetric membranes. *Polym. Chem.* **2016**, *7* (18), 3076-3089, 10.1039/C6PY00215C. DOI: https://doi.org/10.1039/C6PY00215C.

(20) *International Council for Harmonisation of Technical Requirements for Pharmaceuticals for Human Use. ICH guideline Q3D (R2) on elemental impurities, Step 5*; European Medicines Agency.
